# Strained Porphyrin Tape–Cycloparaphenylene Hybrid Nanorings

**DOI:** 10.1021/acs.orglett.2c04089

**Published:** 2023-01-10

**Authors:** Wojciech Stawski, Jeff M. Van Raden, Connor W. Patrick, Peter N. Horton, Simon J. Coles, Harry L. Anderson

**Affiliations:** † Department of Chemistry, Chemistry Research Laboratory, University of Oxford, Oxford OX1 3TA, U.K.; ‡ UK National Crystallographic Service, Chemistry, University of Southampton, Highfield, Southampton SO17 1BJ, U.K.

## Abstract

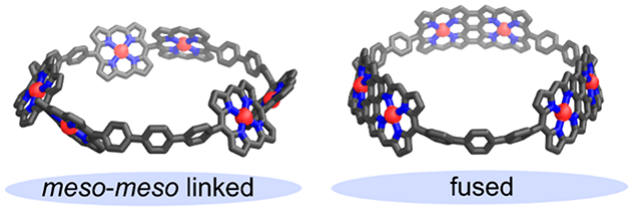

V-Shaped porphyrin dimers, with masked *p-*phenylene bridges, undergo efficient oxidative coupling to form *meso*–*meso* linked cyclic porphyrin oligomers. Reductive aromatization unmasks the *p*-phenylenes, increasing the strain. Oxidation then fuses the porphyrin dimers, providing a nanoring with curved walls. The strain in this macrocycle bends the *p*-phenylene and fused porphyrin dimer units (radii of curvature of 11.4 and 19.0 Å, respectively), but it does not significantly alter the electronic structure of the fused porphyrins.

π-Conjugated macrocycles^2^ exhibit fascinating behavior, such as exciton delocalization,^3,4^ global aromaticity,^5^ amplified two-photon absorption,^6^ Möbius topology,^7^ host–guest chemistry,^8,9^ enhanced semiconductivity,^10^ and strain-induced reactivity.^11^ Ring strain can favor orbital overlap by preventing neighboring subunits from twisting out of conjugation,^12^ while bending can also reduce the π–π* energy gap.^13^ Porphyrins are some of the most versatile components for constructing π-extended frameworks.^14^ Porphyrin nanorings mimic the ultrafast energy migration in photosynthetic light-harvesting chlorophyll arrays.^4,15,16^ Oxidized, reduced, and photochemically excited porphyrin nanorings are also the largest macrocycles yet to exhibit global (anti)aromatic ring currents.^5^ Here we present the synthesis of a strained nanoring, with three edge-fused porphyrin dimer units, via oxidative porphyrin–porphyrin coupling (Scheme 1).^1^

**Scheme 1:**
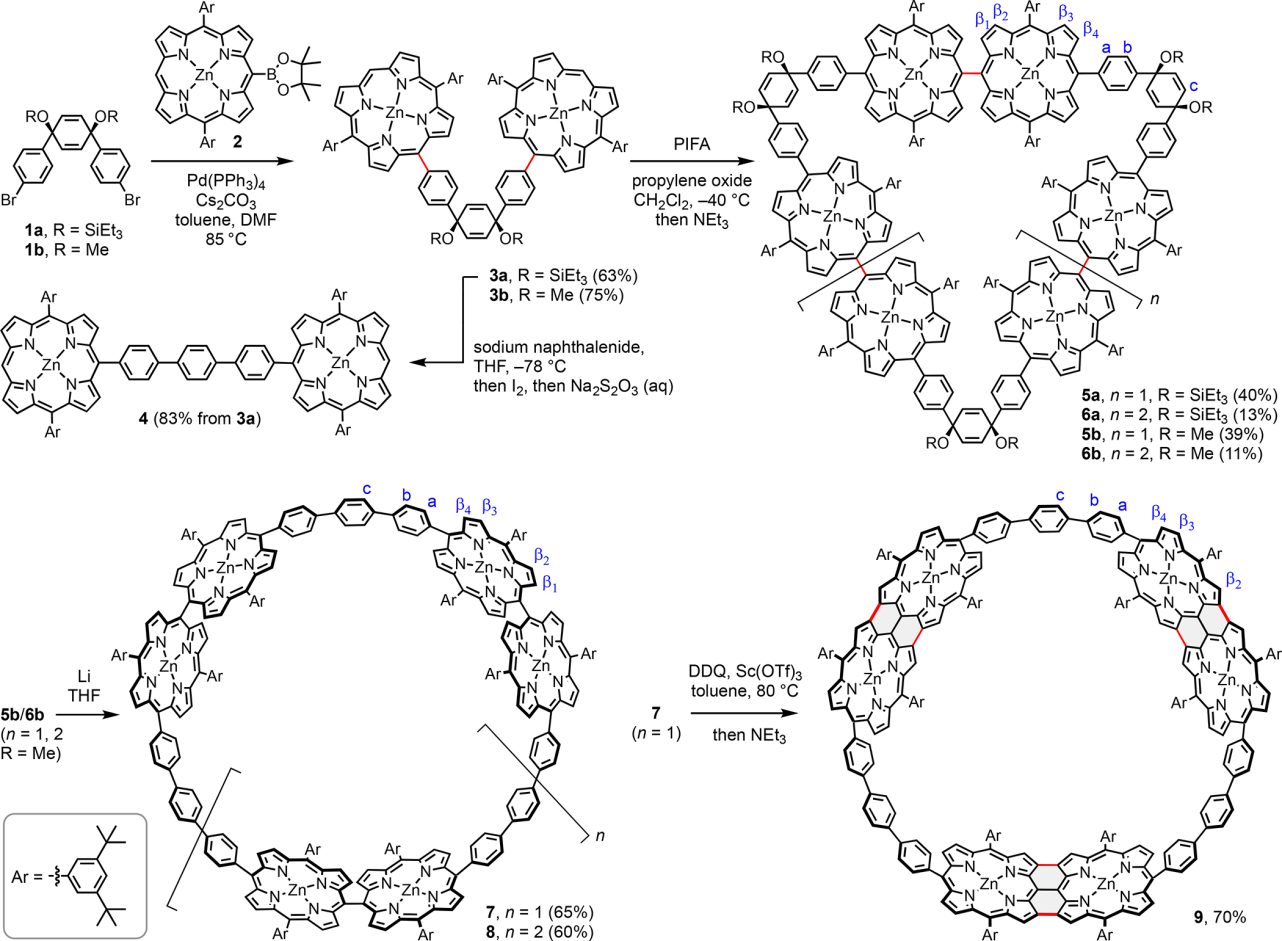
Synthetic Pathway The blue atom labels are used to assign the ^1^H NMR resonances in Figure 2.

Electron-rich aromatic compounds, such as porphyrins, can often be oxidatively oligomerized.^17^ Porphyrins with unsubstituted *meso* positions readily undergo oxidative coupling to form *meso–meso* linked oligomers,^18^ and further oxidation stitches together the β positions to generate *β,meso,β* edge-fused porphyrin tapes.^19^ Here, we apply oxidative coupling of a V-shaped porphyrin dimer **3a,b**, followed by reductive aromatization of the *p*-phenylene linker, to yield the strained *p*-phenylene linked porphyrin nanorings **7** and **8**. Intramolecular oxidative fusion of the porphyrin units in **7** gives nanoring **9**, which features three curved edge-fused porphyrin dimer tape motifs.

There have been a few reports of macrocyclization via oxidative porphyrin–porphyrin coupling.^20^ Several macrocycles containing a *meso*–*meso* linked porphyrin dimer motif have also been prepared by other routes,^16,21^ but the incorporation of a triply fused porphyrin tape into a nanoring has scarcely been explored.^22^ We are interested in synthesizing strained nanorings incorporating porphyrin tapes, to test how strain affects the electronic properties of the edge-fused porphyrin oligomers.

The cyclohexadienyl motif in the V-shaped porphyrin dimer **3a,b** serves as a masked *p-*phenyl bridge. These units can be aromatized using a variety of methods, allowing strain to be added to the nanoring at a late stage in the synthesis.^23−25^ Previously, von Delius and co-workers used this approach to prepare cyclic porphyrin dimers and trimers,^9^ while Osuka and co-workers have prepared *p*-phenylene-linked porphyrin rings using platinum chemistry.^26^

V-Shaped porphyrin dimer **3a** was synthesized in 63% yield by Suzuki–Miyaura coupling of triethylsilyl-protected bromide **1a** and borylated porphyrin **2** (Scheme 1; see the Supporting Information for details). Porphyrins with 3,5-di-*tert-*butylphenyl substituents were used to provide solubility and crystallinity. The V-shaped geometry of **3a**, and the *syn* arrangement around cyclohexadiene, were confirmed by a single-crystal X-ray diffraction analysis (Figure 1). Slow evaporation of a solution of **3a** in a mixture of chloroform and methanol containing pyridine gave monoclinic crystals (space group *I*2/*a*) with one whole molecule per asymmetric unit. One of the zinc(II) ions is coordinated to methanol, and the other is coordinated to pyridine. The angle between the mean planes of the two porphyrins is 80.24(3)°.

**Figure 1:**
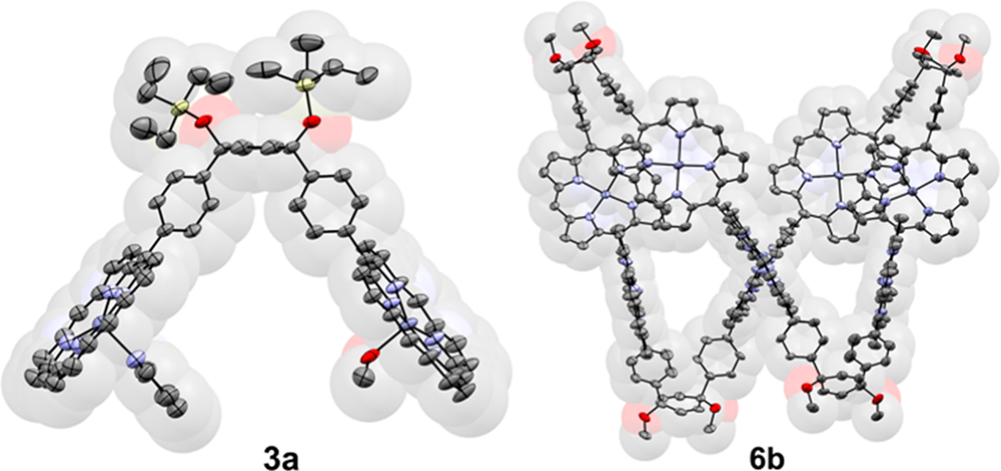
Molecular structures of **3a** and **6b** from X-ray crystallography (50% probability ellipsoids). Hydrogen atoms and aryl groups have been omitted from both structures for the sake of clarity, together with coordinated solvent molecules in **6b**.

Oxidative cyclo-oligomerization of **3a** was achieved using bis(trifluoroacetoxy)iodobenzene (PIFA) at −40 °C, in the presence of propylene oxide as an acid scavenger, generating a mixture of cyclic oligomers, which was separated by thin-layer chromatography (TLC, SiO_2_) to give mainly cyclic porphyrin hexamer **5a** (40% yield), together with some cyclic porphyrin octamer **6a** (13% yield; we did not detect any formation of the cyclic tetramer). The ^1^H and ^13^C NMR spectra reflect the high symmetry of these cyclic oligomers, and their ring sizes were determined from the MALDI mass spectra. Maintaining a low temperature (−40 °C) during this oxidative coupling reaction is important; otherwise, acid-mediated rearrangement occurs,^27^ manifested by the disappearance of cyclohexadienyl ^1^H NMR signals at 6.5–6.6 ppm and reducing the symmetry of the spectra (for details, see the Supporting Information). Use of propylene oxide as a proton scavenger improved the reproducibility of the reaction.

Next, reductive unmasking of the *p-*phenyl ring was tested. When porphyrin dimer **3a** was deprotected (OSiEt_3_ → OH) with tetra-*n*-butylammonium fluoride (TBAF) and then treated with H_2_SnCl_4_ in THF, followed by reinsertion of zinc(II), the product gave a complex ^1^H NMR spectrum, and MALDI MS analysis indicated the loss of only one oxygen atom rather than two, suggesting rearrangement with migration of a phenyl moiety (see Figure S69). This behavior was reported previously during syntheses of cycloparaphenylenes (CPPs).^28^ Similar undesired reactivity was observed when using tin(II) chloride dihydrate without the addition of acid^29^ and when the same conditions were applied to macrocycles **5a** and **6a**. Encouraged by recent reports by von Delius and co-workers,^9^ we also attempted the aromatization on a nickel(II) complex instead of zinc(II), but this was similarly unsuccessful (see section 4c of the Supporting Information). Moreover, attempted cyclization of the Ni^II^ version of **3a** failed because it is not sufficiently electron-rich to undergo oxidative coupling at low temperatures,^30^ while increasing the temperature leads to rearrangement and loss of the cyclohexadienyl signal in the ^1^H NMR spectrum (Figure S71).

Bearing in mind the acid sensitivity of the cyclohexadienyl derivatives, we decided to test non-acidic aromatization conditions. **3a** was successfully aromatized by reaction with sodium naphthalenide at −78 °C in dry THF, before quenching with iodine and isolating product **4** in 83% yield (see Scheme 1).^31^ However, applying the same reaction conditions to cyclic oligomers **5a** and **6a** gave complex mixtures of unidentified products. After many unsuccessful attempts at the aromatization of **5a** and **6a** using various reducing agents (e.g., SnCl_2_, LiDBB, and low-valent Ti),^24^ we decided to change our strategy and work with methoxy derivatives (OR = OMe, rather than OSiEt_3_ or OH).

Porphyrin dimer **3b** (the methoxy analogue of **3a**) was synthesized using similar reaction conditions in even higher yield (75%). Subjecting it to our optimized PIFA-mediated coupling conditions provided cyclic oligomers **5b** and **6b** in 39% and 11% yields, respectively. Single crystals of **5b** and **6b** were grown by vapor diffusion of methanol into a solution in toluene and slow evaporation of a solution in chloroform and methanol, respectively. Porphyrin octamer **6b** crystallizes in a tetragonal *I*4_1_/*a* space group with two porphyrin moieties in the asymmetric unit, with a dihedral angle of 85.21(4)° between the 24-atom mean planes of the *meso*-linked porphyrins. The geometry of cyclohexadiene within each bisporphyrin unit enforces a tub-like conformation. Each zinc(II) center is coordinated to a methanol molecule (Figure 1). Trigonal (*R*3̅*c*) **5b** adopts a more predictable triangularly shaped geometry with an angle between porphyrin mean planes of 89.23(4)° (Figure S79).

To our delight, after screening many conditions (see section 4 of the Supporting Information), we found that both **5b** and **6b** undergo reductive aromatization upon treatment with excess lithium metal in THF at 20 °C to form **7** and **8** in 65% and 60% yields, respectively. A critical factor for these reactions is the use of a glass-coated stirring bar. The reactions failed when PTFE-coated stirring bars were used, and it appears that the reduction products of PTFE interfere with aromatization.^32,33^ Upon conversion of **5b**/**6b** to **7**/**8**, the cyclohexadienyl signals in the ^1^H NMR spectra (**5b**, δ = 6.71 ppm; **6b**, δ = 6.68 ppm) are replaced by *p*-phenyl singlets [**7**, δ = 8.06 ppm; **8**, δ = 8.09 ppm (see Figure 2 and Figures S40 and S54)].

**Figure 2:**
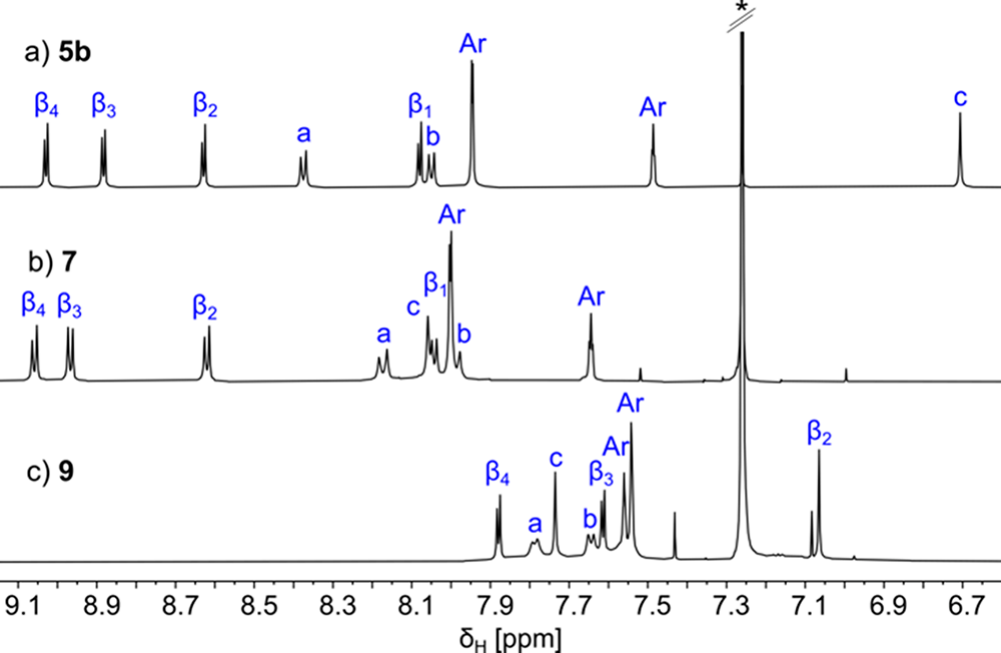
Partial ^1^H NMR spectra of cyclic porphyrin hexamers **5b**, **7**, and **9** [CDCl_3_, 600 MHz, 300 K (see Scheme 1 for labeling)].

Subjecting porphyrin hexamer **7** to DDQ/Sc(OTf)_3_ gave *β,meso,β* edge-fused product **9** in 70% yield. The solubility decreases drastically during the reaction, as expected from the formation of a more rigid structure with planar walls. Attempts to fuse octamer **8** were unsuccessful, perhaps due to the poor solubility of the product (Figure S78).

The UV–vis absorption spectra of cyclic porphyrin hexamers **5b**, **7**, and **9** are compared in Figure 3. Compounds **5b** and **7** have similar absorption spectra, showing that aromatization of the *p-*phenylene bridges does not significantly alter the electronic structure of the porphyrins. This conclusion is confirmed by the similar redox potentials of **5b** and **7** (Figure S77). Fusion of the porphyrins shifts the Q-band to the near-infrared (NIR) range at 1059 nm for **9**, which is similar to the fused linear dimer **10** (1042 nm), indicating that the strain in **9** and π-conjugation through the *p*-phenylene bridges do not strongly affect the electronic structure of the fused porphyrin dimer units. The cyclic and square-wave voltammograms of **9** and **10** (Figure 3b) confirm that they have similar electronic structures. The absorption spectra of porphyrin octamers **6b** and **8** resemble those of hexamers **5b** and **7** (see the Supporting Information).

**Figure 3:**
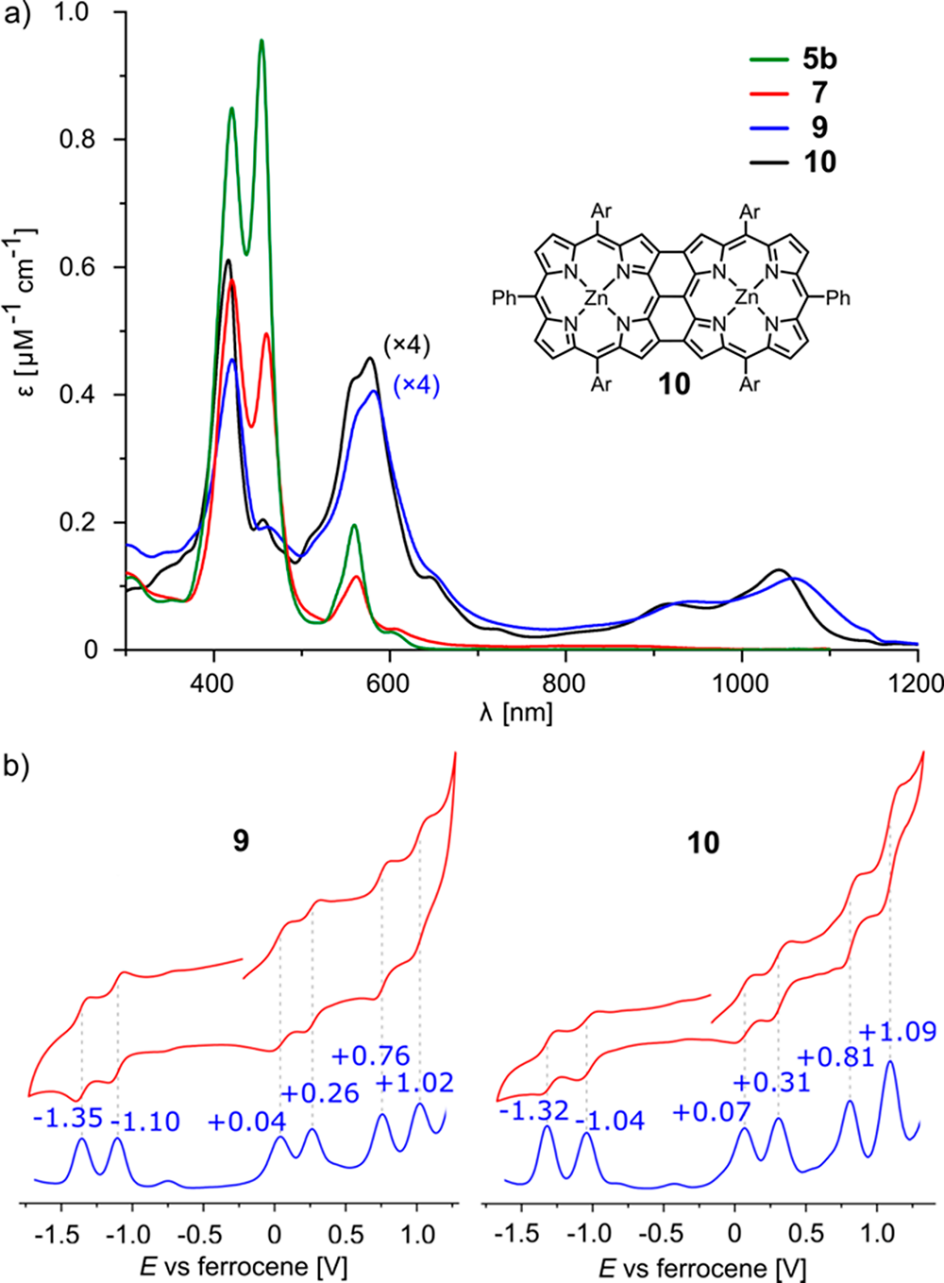
(a) UV–vis–NIR absorption spectra of cyclic porphyrin hexamers **5b**, **7**, and **9** and reference linear dimer **10**, recorded in CH_2_Cl_2_. (b) Cyclic (red) and square-wave (blue) voltammograms of **9** and **10** recorded in CH_2_Cl_2_ containing 0.1 M Bu_4_NPF_6_. Potentials relative to internal ferrocene (Fc/Fc^+^ at 0 V).

The geometries and electronic structure of nanorings **7–9** were modeled using density functional theory (DFT) in the gas phase at the B3LYP 6-31G(d,p) level of theory (with the Ar solubilizing groups replaced by H to simplify the calculations). 6-Porphyrin rings **7** and **9** have diameters of 28.5 and 28.2 Å, respectively [measured from the centroid of a benzene to the centroid of the opposite *meso–meso* bond (Figure 4)]. 8-Porphyrin ring **8** has a diameter of 38.6 Å (measured from benzene to benzene). In **7** and **8**, the *meso–meso* linked porphyrins are roughly orthogonal (β-*meso*-*meso*-β torsion angles of 75.7° in **7** and 81.1° in **8**), as expected, and there is a substantial twist between each porphyrin and its *meso*-linked *p*-phenylene (torsion angled of 66.3° in **7** and 64.8° in **8**), with a smaller twist at the biphenyl connections (torsion angles of 32.3° in **7** and 35.8° in **8**). The torsion angles are similar in **9** (*meso*-linked *p*-phenylene, 59.2°; phenylene–phenylene, 37.7°), which implies that there is only weak π-conjugation around the circumference of the nanoring.

**Figure 4:**
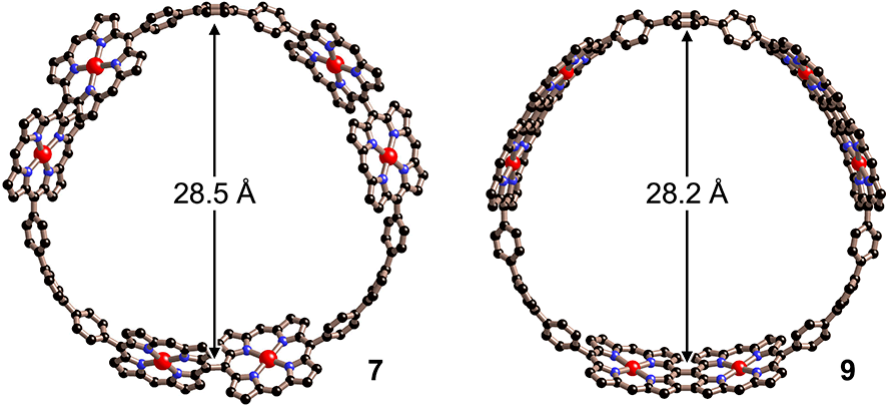
DFT-optimized structures of **7** and **9** (H atoms and *meso*-aryl substituents omitted solubilized).

The curvatures of the terphenylene and porphyrin dimer units in **7** and **9** were estimated by fitting the coordinates to an arc (see the Supporting Information for details). In **7**, the radii of curvature of the terphenylene and porphyrin dimer units are 11.7 and 17.1 Å, respectively, which shows that the terphenylene bridges are more flexible than the porphyrins. The curvature in the *p*-phenylenes matches that in [17]CPP. The radii of curvature are similar in **9** (terphenylene, 11.8 Å; fused porphyrin dimer, 19.0 Å). This corresponds to the predicted curvature in a fully edge-fused 14-porphyrin nanobelt.

The strain in the nanorings was estimated by analyzing homodesmotic reactions (Table S3). As expected, **7** (154 kJ mol^–1^) is more stained than **8** (112 kJ mol^–1^) and the strain does not change significantly upon fusion of **7** to **9** (154 kJ mol^–1^). These six-porphyrin nanorings are more strained than a butadiyne-linked six-porphyrin nanoring (100 kJ mol^–1^)^34^ and are close to [16]CPP (149 kJ mol^–1^) for **7**/**9** and to [20]CPP (119 kJ mol^–1^) for **8**.^35^ The calculated Kohn–Sham HOMO and LUMO orbitals of **9** are located on porphyrin units and have coefficients close to zero on the *p*-phenylene linkers (see the Supporting Information), which is consistent with the experimental observations from UV–vis–NIR spectra and voltammograms (Figure 3b and Figure S77). The electronic delocalization in the fused porphyrin dimer units in **9** is undisturbed by a substantial deviation from planarity.

In conclusion, we have demonstrated an efficient synthetic pathway to macrocyclic CPP–porphyrin hybrids using oxidative homocoupling of the porphyrins as the macrocyclization step. It is remarkable that macrocyclic products **5a** and **6a** are formed in good yields (40% and 13%, respectively) without using a template. The methoxy versions of these macrocycles, **5b** and **6b**, respectively, were synthesized in similar yields, and their crystal structures were determined. Aromatization of the terphenylene bridges and fusion of the porphyrin units in the six-porphyrin nanoring give macrocycle **9**, which incorporates three curved fused porphyrin dimers, although the strain does not significantly affect the electronic structure.

## Data Availability

The data underlying this study are available in the published article and its Supporting Information.

## Supporting Information Available

General methods, synthesis, and characterization of all compounds (PDF)
