# Supplementary material for: Strained Porphyrin Tape–Cycloparaphenylene Hybrid Nanorings
Source: Org Lett. 2023 Jan 10;25(2):378–83. doi: 10.1021/acs.orglett.2c04089 (PMC9872170; doi:10.1021/acs.orglett.2c04089)
Supplement: Supplementary file 1 — ol2c04089_si_001.pdf [file ol2c04089_si_001.pdf]

Supporting Information

Strained Porphyrin Tape-Cycloparaphenylene Hybrid Nanorings

Wojciech Stawski,[§] Jeff M. Van Raden,[§] Connor W. Patrick,[§] Peter N. Horton,[‡] Simon J. Coles[‡] and Harry L. Anderson*[,§]

[§]*Department of Chemistry, Chemistry Research Laboratory, University of Oxford, Oxford OX1 3TA, U.K.*
[‡]*UK National Crystallographic Service, Chemistry, University of Southampton, Highfield, Southampton SO17 1BJ, U.K.*

Table of Contents

Section 1.General methods S1
Section 2. Synthetic procedures S2
Section 3. NMR and mass spectra S9
Section 4. Unsuccessful aromatization, cyclization and fusion attempts S41
Section 5. X-ray crystallography S47
Section 6. DFT calculations S51
Section 7. Electrochemistry S65
Section 8. References S66

1. General Methods

Dichloromethane, toluene and DMF for reactions were obtained from an MBraun MBSPS-5-BenchTop solvent purification system (SPS) under nitrogen. Chloroform-*d* for NMR was stored over K_2CO_3 and passed through a short neutral alumina plug prior to use. All other reagents and solvents were obtained from commercial suppliers and used as received unless otherwise stated.

Thin-layer chromatography (TLC) was carried out using commercially available (Merck) aluminum sheets precoated with silica gel with fluorescence indicator and visualized under UV light at 254 or 360 nm. Purification by column chromatography was carried out on silica gel (SiO_2, 60 Å, 40−63 µm, Merck). Preparative Analtech Uniplate preparative TLC plates were used. Size exclusion chromatography (SEC) was carried out using Bio-Rad Bio-Beads S-X1 (40–80 µm bead size).

^{1}H and ^{13}C NMR spectra were recorded on either a Bruker AVIII HD 400, a Bruker AVIII HD 500, or a Bruker AVIII 600 with a broadband cryo-probe. Chemical shift values are quoted in ppm and coupling constants (*J*, reported $^3J_{H\text{-}H}$ if not indicated differently) in Hertz to the nearest 0.1 Hz. ^{1}H and ^{13}C NMR spectra are referenced against the residual solvent peak ($CHCl_3$ δ_H = 7.26 ppm, $CDCl_3$ δ_C = 77.16 ppm. UV-Vis-NIR measurements were carried out in a 1 cm path length glass cuvette at 298 K using either a Perkin Lambda 20 or a Jasco V770 spectrophotometer.

MALDI-TOF mass spectra were recorded using a Bruker Autoflex instrument with DCTB as a matrix. Calibration was performed before each measurement using Peptide Standard II for the 700–3500 Da Protein Standard I for the 5–20 kDa window (Bruker). Mass spectra of compounds with molecular weight up to 3.5 kDa were measured in a reflectron mode, whereas for larger mass a linear mode was used.

Electrospray mass spectrometry was carried out on a Waters Micromass LCT Premier XE spectrometer using 90:10 MeOH:H_2O (+0.1% formic acid) as the mobile phase. High-resolution mass spectrometry (HR-MS) measurements were performed on a Thermo Orbitrap Exactive MS with Waters Acquity Ultraperformance LC system.

2. Synthetic Procedures

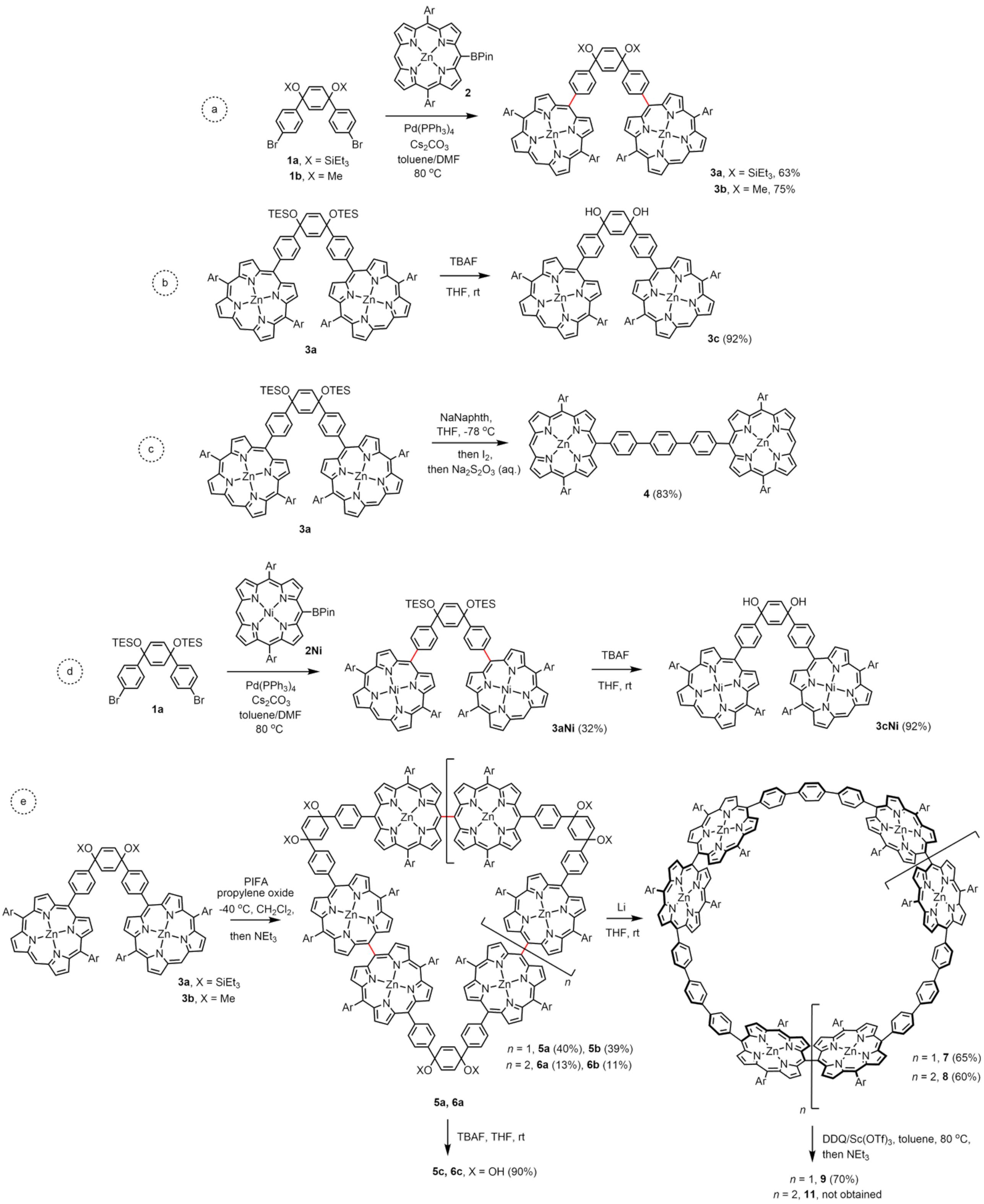

Scheme S1. Additional synthetic schemes.

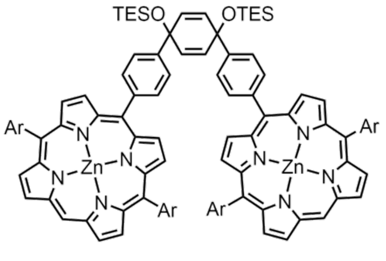

Synthesis of Porphyrin Dimer 3a. Borylated porphyrin **2**[1a] (280 mg, 0.320 mmol, 2.1 equiv.), dibromide **1a**[2] (99.0 mg, 0.152 mmol, 1 equiv.), Cs_2CO_3 (109 mg, 0.335 mmol, 2.2 equiv.) and $Pd(PPh_3)_4$ (21.1 mg, 0.0183 mmol, 0.12 equiv.) were placed in a Schlenk tube, dried under vacuum for 1 h and the atmosphere was changed to argon, then anhydrous, degassed toluene (4 mL) and DMF (2 mL) were added and the mixture was degassed by a freeze-pump-thaw technique. The mixture was heated at 80 °C on an oil bath for 16 h. After completion, the solvents were evaporated and the mixture was passed through a short silica gel plug, eluting with DCM. The solvent was evaporated and the mixture was subjected to SEC chromatography in THF, collecting first the most intensively colored pink fraction (second fraction overall). After evaporation of the solvent, recrystallization from DCM/MeOH was performed on a rotary evaporator followed by filtration, collecting pink solid as a pure product. Yield of **3a**: 63% (191 mg).

^{1}H NMR (500 MHz, $CDCl_3$) δ 10.22 (s, 2H), 9.37 (d, *J* = 4.5 Hz, 4H), 9.08 (d, *J* = 4.5 Hz, 4H), 8.97 (d, *J* = 4.7 Hz, 4H), 8.93 (d, *J* = 4.7 Hz, 4H), 8.20 (d, *J* = 8.3 Hz, 4H), 8.00 (d, *J* = 1.8 Hz, 8H), 7.94 (d, *J* = 8.3 Hz, 4H), 7.69 (t, *J* = 1.8 Hz, 4H), 6.52 (s, 4H), 1.40 (s, 72H), 1.18 (t, *J* = 7.9 Hz, 18H), 0.90 (q, *J* = 7.9 Hz, 12H).

^{13}C NMR (126 MHz, $CDCl_3$) δ 150.38, 150.35, 149.8, 149.7, 148.5, 145.5, 142.0, 141.6, 134.2, 132.9, 132.1, 132.0, 131.8, 131.5, 129.8, 124.1, 121.9, 121.0, 120.7, 105.7, 71.8, 34.9, 31.7, 7.3, 6.8.

HRMS (MALDI) *m/z*: $[M]^+$ Calcd for $C_{126}H_{144}N_8O_2Si_2Zn_2$ 1984.9528; Found: 1984.9985.

UV/Vis (CH_2Cl_2, 298 K) λ, log ε: 415(6.07), 506(3.87), 544 (4.73), 582 (3.82).

Cyclization of 3a to give 5a and 6a. The cyclization substrate **3a** (100 mg, 0.05 mmol, 1 equiv.) was dissolved in dry DCM (75 mL) under argon atmosphere. Propylene oxide (90 µL, 1.34 mmol, 27 equiv.) was added and the mixture was cooled to –40 °C using a MeCN/dry ice bath. A solution of PIFA (28.1 mg, 0.063 mmol, 1.3 equiv.) in dry DCM (2 mL) was added dropwise and the reaction mixture was stirred for 2.5 h at –40 °C. A gradual color change from pink to orange was observed. After completion, triethylamine (0.7 mL, 5.0 mmol, 100 equiv.) was added and the mixture was allowed to warm to room temperature. The solvents were evaporated. A short silica gel plug (DCM + 1% triethylamine) was performed, collecting the orange fraction which contains a mixture of cyclic oligomers. Nanorings were separated from each other using preparative TLC plates, using 3:1 DCM/hexane + 1% triethylamine as an eluent, eluting first **5a** and then **6a**. After evaporation of the solvents and precipitation on a rotary evaporator from DCM/MeOH followed by filtration, nanorings were obtained as brown solids. Yield: **5a** 40% (40 mg), **6a** 13% (13 mg).

5a

^{1}H NMR (600 MHz, $CDCl_3$) δ 8.99 (d, *J* = 4.6 Hz, 12H), 8.85 (d, *J* = 4.6 Hz, 12H), 8.61 (d, *J* = 4.6 Hz, 12H), 8.31 (d, *J* = 7.9 Hz, 12H), 8.06 (d, *J* = 4.6 Hz, 12H), 7.99 (d, *J* = 8.0 Hz, 12H), 7.93 (d, *J* = 1.6 Hz, 24H), 7.46 (t, *J* = 1.6 Hz, 12H), 6.57 (s, 12H), 1.21 (t, *J* = 7.9 Hz, 54H), 1.18 (s, 216H), 0.93 (q, *J* = 7.9 Hz, 36H).

^{13}C NMR (151 MHz, $CDCl_3$) δ 154.7, 150.9, 150.0, 148.3, 145.5, 142.0, 141.5, 134.3, 133.7, 132.1 (overlapping peaks), 131.8, 129.5, 124.3, 123.2, 121.4, 120.5, 119.4, 71.9, 34.7, 31.5, 7.3, 6.8.

HRMS (MALDI) *m/z*: $[M+Na]^+$ Calcd for $C_{378}H_{426}N_{24}O_6Si_6Zn_6Na$ 5984.8046; Found: 5984.7995.

UV/Vis (CH_2Cl_2, 298 K) λ, log ε: 419 (5.71), 453 (5.74), 559 (5.08), 600 (4.23).

6a

^{1}H NMR (400 MHz, $CDCl_3$) δ 8.99 (d, *J* = 4.7 Hz, 16H), 8.89 (d, *J* = 4.7 Hz, 16H), 8.58 (d, *J* = 4.8 Hz, 16H), 8.28 (d, *J* = 7.9 Hz, 16H), 8.01 (d, *J* = 4.7 Hz, 16H), 7.98 (d, *J* = 7.9 Hz, 16H), 7.94 (d, *J* = 1.8 Hz, 32H), 7.53 (t, *J* = 1.8 Hz, 16H), 6.54 (s, 16H), 1.26 (s, 288 H), 1.19 (t, *J* = 7.9 Hz, 72H), 0.92 (q, *J* = 7.9 Hz, 48H).

^{13}C NMR (101 MHz, $CDCl_3$, from HSQC experiment) δ 134.3, 133.7, 132.2, 132.12, 132.14, 131.9, 129.5, 124.2, 120.6, 31.5, 7.3, 6.9 (only CH, CH_2 and CH_3 carbons).

HRMS (MALDI) *m/z*: $[M]^+$ Calcd for $C_{504}H_{568}N_{32}O_8Si_8Zn_8$ 7949.7532; Found: 7950.0483.

UV/Vis (CH_2Cl_2, 298 K) λ, log ε: 419 (5.92), 454 (5.90), 558 (5.28), 599 (4.39).

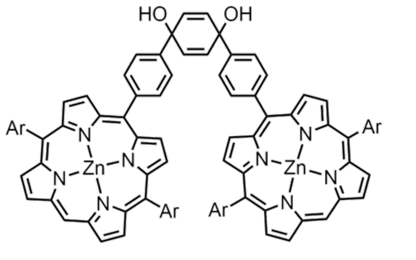

Synthesis of Porphyrin Dimer 3c. A solution of TBAF (1.0 M in THF, 50 µL, 0.05 mmol, 5 equiv.) was added to a solution of **3a** (20 mg, 0.01 mmol, 1 equiv.) in dry THF (2 mL) under argon and a solution was stirred for 30 min at room temperature. The reaction was quenched by addition of water (0.5 mL). Most of the THF was evaporated under a stream of nitrogen and the resulting mixture was treated with methanol (2 mL). The precipitate was filtered off on wool with a small amount of hexane, redissolved in dichloromethane and evaporated in vacuo to dryness to give **3c** as a pink solid. Yield: 15.9 mg (90%).

^{1}H NMR (600 MHz, $CDCl_3$ + 1% pyr-d_5) δ 10.09 (s, 2H), 9.28 (d, J = 4.4 Hz, 4H), 9.01 (d, J = 4.4 Hz, 4H), 8.90 (AB quartet, 8H), 8.23 (d, J = 8.4 Hz, 4H), 8.00 (d, J = 1.8 Hz, 8H), 7.97 (d, J = 8.4 Hz, 4H), 7.71 (t, J = 1.8 Hz, 4H), 6.58 (s, 4H), 2.94 (s, 2H), 1.46 (s, 72H).

^{13}C NMR (151 MHz, $CDCl_3$ + 1% pyr-d_5) δ 150.3, 150.2, 149.6, 149.5, 148.2, 143.3, 142.8, 142.3, 134.7, 132.7, 132.5, 131.8, 131.4, 131.2, 130.0, 123.4, 121.4, 120.4, 120.1, 105.2, 69.7, 34.9, 31.7.

HRMS (MALDI) *m/z*: $[M]^+$ Calcd for $C_{114}H_{116}N_8O_2Zn_2$ 1756.7799; Found: 1756.8772.

UV/Vis (CH_2Cl_2, 298 K), λ, log ε: 414.5(6.31), 543 (4.96).

Deprotection of TESO-protected nanorings 5a and 6a to give 5c and 6c. A solution of TBAF (1.0 M in THF, 15 equiv., 24 µL) was added to a solution of **5a** (10 mg, 1 equiv., 1.6 µmol) in dry THF (1 mL) under argon and followed by stirring for 30 min at room temperature. The reaction was quenched by addition of water (0.5 mL). Most of THF was evaporated under a stream of nitrogen and the formed mixture of water and precipitate of a reaction product was treated with methanol (2 mL) and filtered through cotton wool which followed by washing with a small amount of hexane. Solid was redissolved in dichloromethane and evaporated in vacuo to dryness to give a brownish solid. The product was used quickly directly in next step without further characterization other than confirming completion by ^{1}H NMR and mass spectrometry. Yield of **5c**: 90% (8.8 mg)

6c was not isolated as a pure substance, only as a mixture with **5c**: A mixture of **5a** and **6a** purified after cyclization (10 mg) was dissolved in dry THF (1 mL) under argon and a solution of TBAF (1.0 M in THF, 1.6 µmol, 24 µL) was added followed by stirring of the mixture for 30 min at room temperature. Reaction was quenched by addition of water (0.5 mL). Most of the THF was evaporated under a stream of nitrogen and the resulting mixture was treated with methanol (2 mL). The precipitate was filtered off on cotton wool with a small amount of hexane, redissolved in dichloromethane and evaporated in vacuo to dryness to give a brownish solid. The material was used quickly directly in next step without further characterization other than confirming formation of the product by ^{1}H NMR and mass spectrometry. Yield of mixture of **5c** and **6c**: 90% (8.8 mg).

5c

^{1}H NMR (400 MHz, $CDCl_3$) δ 9.02 (d, J = 4.8 Hz, 12H), 8.91 (d, J = 4.7 Hz, 12H), 8.63 (d, J = 4.8 Hz, 12H), 8.37 (d, J = 8.0 Hz, 12H), 8.07 (d, J = 4.8 Hz, 12H), 8.04 (d, J = 8.1 Hz, 12H), 7.96 (d, J = 1.8 Hz, 24H), 7.52 (t, J = 1.8 Hz, 12H), 6.64 (s, 12H), 2.67 (s, 6H), 1.25 (s, 216H).

HRMS (MALDI) *m/z*: $[M]^+$ Calcd for $C_{342}H_{342}N_{24}O_6Zn_6$ 5276.2953; Found: 5276.2483.

6c

HRMS (MALDI) *m/z*: $[M]^+$ Calcd for $C_{456}H_{456}N_{32}O_8Zn_8$ 7035.0607; Found: 7036.0282

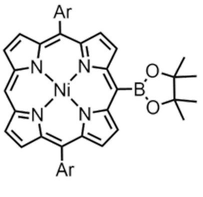

Preparation of nickel porphyrin 2Ni. Borylated zinc porphyrin **2** (100 mg, 0.114 mmol, 1 equiv.) was dissolved in dichloromethane (60 mL) and treated with concentrated hydrochloric acid (0.78 mL, 9.13 mmol, 80 equiv.). After stirring at room temperature for 1 h, the acid was neutralized with saturated aqueous solution of $NaHCO_3$ and thus obtained metal-free porphyrin was extracted with dichloromethane (2x50 mL). Combined organic extracts were dried over anhydrous Na_2SO_4 and solvent was evaporated. The solid was dissolved in toluene (20 mL) together with nickel(II) acetylacetonate (293 mg, 1.14 mmol, 10 equiv.) and heated at reflux on an oil bath for 5 h. After completion and evaporation of the solvent, short silica gel plug in dichloromethane was performed. Evaporation of the solvent provided **2Ni** as a red solid with 85% (85 mg) yield. The compound is used as a substrate in two papers but synthetic procedure and spectroscopic data was not reported.[1b-c]

^{1}H NMR (400 MHz, $CDCl_3$) δ 9.84 (s, 1H), 9.79 (d, J = 4.8 Hz, 2H), 9.14 (d, J = 4.8 Hz, 2H), 8.94 (d, J = 4.8 Hz, 2H), 8.92 (d, J = 4.8 Hz, 2H), 7.90 (d, J = 1.8 Hz, 4H), 7.77 (t, J = 1.9 Hz, 2H), 1.73 (s, 12H), 1.52 (s, 36H).

^{13}C NMR (101 MHz, $CDCl_3$) δ 149.1, 146.5, 143.3, 142.5, 141.9, 140.2, 133.6, 133.4, 132.4, 132.2, 129.0, 121.2, 119.8, 105.8, 85.0, 35.2, 31.9, 25.3.

HRMS (ESI) *m/z*: $[M+H]^+$ Calcd for $C_{54}H_{63}BN_4NiO_2$ 869.4470; Found: 869.4462.

UV/Vis (CH_2Cl_2, 298 K), λ, log ε: 407.5 (5.57), 523 (4.35), 555 (4.05).

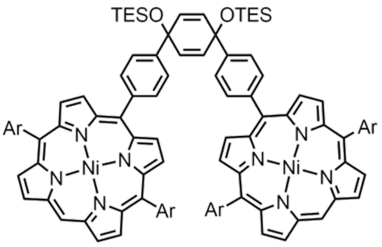

Synthesis of Porphyrin Dimer 3aNi. Borylated porphyrin **2Ni** (146 mg, 0.168 mmol, 2.1 equiv.), dibromide **1a**[2] (52.0 mg, 0.080 mmol, 1 equiv.), Cs_2CO_3 (57 mg, 0.176 mmol, 2.2 equiv.) and $Pd(PPh_3)_4$ (11.1 mg, 0.0096 mmol, 0.12 equiv.) were placed in a Schlenk tube, dried under vacuum for 1 h and the atmosphere was changed to argon. Anhydrous, degassed toluene (2 mL) and DMF (1 mL) were added and the mixture was degassed by a freeze-pump-thaw technique. The mixture was heated at 80 °C in an oil bath for 16 h. The solvent was evaporated and the mixture was passed through a short silica gel plug, eluting with DCM. The solvent was evaporated and the mixture was subjected to SEC chromatography in THF, collecting first the most intensively colored red fraction (second fraction overall). After evaporation of the solvent, precipitation from DCM/MeOH was performed on a rotary evaporator and the solid was filtered, collecting the red precipitate which was then purified by column chromatography (SiO_2, petrol/DCM : 5/1 + 1% triethylamine), collecting the intense red band. Precipitation from dichloromethane/methanol on a rotary evaporator and filtration provided pure product **3aNi** as a red solid. Yield: 32% (50 mg)

^{1}H NMR (600 MHz, $CDCl_3$) δ 9.79 (s, 2H), 9.09 (d, *J* = 4.7 Hz, 4H), 8.88 (d, *J* = 4.7 Hz, 4H), 8.75 (d, *J* = 4.9 Hz, 4H), 8.73 (d, *J* = 4.9 Hz, 4H), 7.99 (d, *J* = 8.3 Hz, 4H), 7.83 (d, *J* = 8.4 Hz, 4H), 7.80 (d, *J* = 1.8 Hz, 8H), 7.64 (t, *J* = 1.8 Hz, 4H), 6.41 (s, 4H), 1.38 (s, 72H), 1.12 (t, *J* = 7.9 Hz, 18H), 0.84 (q, *J* = 7.9 Hz, 12H)

^{13}C NMR (151 MHz, $CDCl_3$) δ 147.8, 144.6, 141.9, 141.8, 141.6, 141.3, 139.2, 138.9, 132.5, 131.7, 131.2, 130.9, 130.74, 130.71, 127.8, 123.3, 119.9, 118.8, 117.9, 103.3, 70.6, 33.9, 30.5, 6.2, 5.6.

HRMS (MALDI) *m/z*: $[M]^+$ Calcd for $C_{126}H_{144}N_8Ni_2O_2Si_2$ 1972.9652; Found: 1972.9762.

UV/Vis (CH_2Cl_2, 298 K) λ, log ε: 408 (5.69), 521 (4.56).

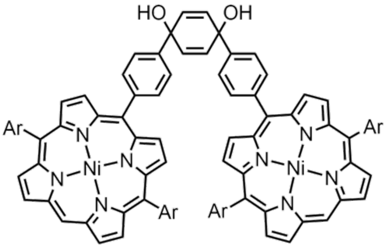

Synthesis of Porphyrin Dimer 3cNi. A solution of TBAF (1.0 M in THF, 60 µL, 0.03 mmol, 5 equiv.) was added to a solution of **3aNi** (10 mg, 0.005 mmol, 1 equiv.) in dry THF (2 mL) and followed by stirring for 30 min at room temperature under argon. The reaction was quenched by addition of water (0.5 mL). Most of the THF was evaporated under a stream of nitrogen and the resulting mixture was treated with methanol (2 mL). The red precipitate was filtered off on cotton wool, redissolved in dichloromethane and evaporated in vacuo to dryness. Yield of **3cNi**: 8.0 mg (92%).

^{1}H NMR (400 MHz, $CDCl_3$) δ 9.80 (s, 2H), 9.10 (d, *J* = 4.8 Hz, 4H), 8.89 (d, *J* = 4.8 Hz, 4H), 8.78 (d, *J* = 4.8 Hz, 4H), 8.76 (d, *J* = 4.8 Hz, 4H), 8.06 (d, *J* = 8.4 Hz, 4H), 7.91 (d, *J* = 8.3 Hz, 4H), 7.83 (d, *J* = 1.8 Hz, 8H), 7.68 (t, *J* = 1.8 Hz, 4H), 6.50 (s, 4H), 1.42 (s, 72H).

^{13}C NMR (151 MHz, $CDCl_3$) δ 148.9, 143.3, 143.0, 142.9, 142.7, 142.3, 140.8, 139.9, 134.0, 132.8, 132.6, 132.3, 131.9, 131.8, 128.9, 124.0, 121.0, 120.0, 118.7, 104.4, 69.6, 34.9, 31.6.

HRMS (MALDI) *m/z*: $[M]^+$ Calcd for $C_{114}H_{116}N_8Ni_2O_2$ 1744.7923; Found: 1744.6343.

UV/Vis (CH_2Cl_2, 298 K) λ, log ε: 408.5 (5.42), 521.5 (4.30).

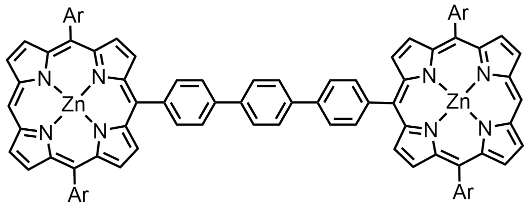

Synthesis of 4. Porphyrin dimer **3a** (10 mg, 0.005 mmol, 1 equiv.) or **3b** (9 mg, 0.005 mmol, 1 equiv.) was placed in a Schlenk flask and dried under high vacuum for 1 h. Dry THF (1.5 mL) was added under argon. The solution was cooled to –78 °C in a dry ice bath and a solution of sodium naphthalenide (0.2 M in THF, 0.5 mL, 0.1 mmol, 20 equiv.) was added dropwise. The mixture was stirred at –78 °C for 20 min and this was accompanied by a color change from brown/orange to blue. While still at –78 °C, a solution of iodine (1.0 M in THF, 130 µL, 25 equiv.) was added, followed by an excess of a saturated solution of sodium thiosulfate (0.2 mL). The mixture was allowed to reach the room temperature, diluted with dichloromethane, washed with water, dried over anhydrous Na_2SO_4 and evaporated to dryness. The solid residue was washed several times with hexane and filtered. Subsequent short column

chromatography using 2:1 DCM/petroleum ether as an eluent provided pure **4** as a pink solid. Isolated yield: 7.2 mg (83%; similar yield is obtained when the methoxy analogue **3b** is used instead of **3a** in the same procedure).

^{1}H NMR (600 MHz, $CDCl_3$) δ 10.13 (s, 2H), 9.33 (d, *J* = 4.4 Hz, 4H), 9.07 (d, *J* = 4.4 Hz, 4H), 9.02 (d, *J* = 4.6 Hz, 4H), 9.00 (d, *J* = 4.6 Hz, 4H), 8.34 (d, *J* = 8.1 Hz, 4H), 8.14 (s, 4H), 8.09 (d, *J* = 1.8 Hz, overlapping, 8H), 8.07 (d, overlapping, 4H), 7.78 (t, *J* = 1.8 Hz, 4H), 1.54 (s, 72H).

^{13}C NMR was not measured directly due to substantial aggregation and low solubility. Data from HSQC experiment (600 MHz, 300 K): δ 135.19, 132.61, 131.65, 131.32, 130.04, 128.10, 124.88, 120.70, 105.25, 31.85 (only CH and CH_3 carbons).

HRMS (MALDI) *m/z*: [M]$^+$ Calcd for $C_{114}H_{114}N_8Zn_2$ 1722.7744; Found: 1722.5678.

UV/Vis (CH_2Cl_2, 298 K) λ, log ε: 417.5 (5.82), 544 (4.48).

Preparation of a 0.2 M solution of sodium naphthalenide: Naphthalene (128 mg, 1 mmol, 1 equiv.) was placed in a pre-dried Schlenk flask equipped with a rubber septum and a glass-coated stirrer bar and the atmosphere was changed to argon. Small pieces of freshly cut sodium metal (excess, ca. 50 mg, 2.2 mmol, 2.2 equiv.) were added quickly which was followed by switching vacuum/argon three times. Dry THF (5 mL) under argon was added through a septum and the mixture was stirred vigorously for 5 h at room temperature, which was accompanied by a change of color from colorless to deep green.

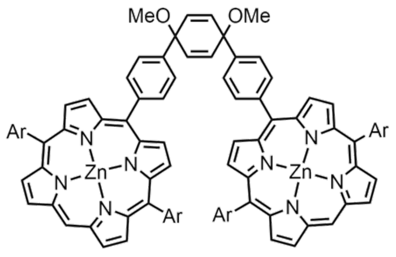

Synthesis of Porphyrin Dimer 3b. Borylated porphyrin **2**[1] (347 mg, 0.397 mmol, 2.1 equiv.), dibromide **1b**[3] (85.0 mg, 0.189 mmol, 1 equiv.), Cs_2CO_3 (135 mg, 0.415 mmol, 2.2 equiv.) and $Pd(PPh_3)_4$ (26.2 mg, 0.0227 mmol, 0.12 equiv.) were placed in a Schlenk tube, dried under vacuum for 1 h and the atmosphere was changed to argon. Then anhydrous, degassed toluene (5 mL) and DMF (2.5 mL) were added and the mixture was degassed using a freeze-pump-thaw technique. The mixture was heated at 80 °C on an oil bath for 16 h. After completion, the solvents were evaporated and the mixture was passed through a short silica gel plug, eluting with DCM. Solvent was evaporated and the mixture was subjected to SEC chromatography (THF), collecting the first intensely pink colored fraction (second fraction overall). After evaporation of the solvent, silica gel chromatography was performed. The first fraction was eluted with 2:1 mixture of petroleum ether and DCM + 1% triethylamine. Then, the eluent was changed to 3:2 DCM/petroleum ether +1% triethylamine and the product was collected as a pink fraction. Solvents were evaporated and recrystallization from DCM/methanol on a rotary evaporator was performed. Drying provided pure product as a pink solid. Yield of **3b**: 254 mg (75%).

^{1}H NMR (600 MHz, $CDCl_3$) δ 10.07 (s, 2H), 9.27 (d, *J* = 4.4 Hz, 4H), 8.99 (d, *J* = 4.4 Hz, 4H), 8.88 (d, *J* = 4.5 Hz, 4H), 8.86 (d, *J* = 4.5 Hz, 4H), 8.21 (d, *J* = 8.1 Hz, 4H), 7.97 (d, *J* = 1.8 Hz, 8H), 7.95 (d, *J* = 8.3 Hz, 4H), 7.69 (t, *J* = 1.8 Hz, 4H), 6.61 (s, 4H), 3.76 (s, 6H), 1.43 (s, 72H).

^{13}C NMR (151 MHz, $CDCl_3$) δ 150.2, 150.1, 149.6, 149.5, 148.2, 143.2, 142.4, 142.3, 134.6, 134.0, 132.4, 131.8, 131.4, 131.1, 130.0, 123.9, 121.4, 120.3, 120.2, 105.1, 75.3, 52.3, 34.9, 31.7.

HRMS (MALDI) *m/z*: [M]$^+$ Calcd for $C_{116}H_{120}N_8O_2Zn_2$ 1784.8111; Found: 1784.9255.

UV/Vis (CH_2Cl_2, 298 K) λ, log ε: 414 (6.25), 542 (4.90).

Cyclization of 3b to give 5b and 6b. The cyclization substrate **3b** (100 mg, 0.056 mmol, 1 equiv.) was dissolved in dry DCM (75 mL) under argon. Propylene oxide (102 µL, 1.51 mmol, 27 equiv.) was added and the mixture was cooled to –40 °C using a MeCN/dry ice bath. A solution of PIFA (31.2 mg, 0.073 mmol, 1.3 equiv.) in dry DCM (2 mL) was added dropwise and the reaction was stirred for 2.5 h at –40 °C. A gradual color change from pink to orange was observed. After completion, triethylamine (0.78 mL, 5.6 mmol, 100 equiv.) was added and the mixture was allowed to warm to room temperature. The solvent was then evaporated and the resulting solid was redissolved in DCM and then passed through a short silica gel plug (DCM + 1% Et_3N), collecting the orange fraction which contains a mixture of a cyclic oligomers. The desired cyclic products were then purified using preparative TLC plates, using 3:1 DCM/hexane + 1% triethylamine as an eluent, eluting first **5b** and then **6b**. Evaporation of the solvents followed by recrystallization from DCM/methanol on a rotary evaporator provided **5b** and **6b** as brown solids. Yield: **5b** 39% (39 mg), **6b** 11% (11 mg).

5b

^{1}H NMR (600 MHz, $CDCl_3$) δ 9.03 (d, *J* = 4.7 Hz, 12H), 8.88 (d, *J* = 4.7 Hz, 12H), 8.63 (d, *J* = 4.7 Hz, 12H), 8.38 (d, *J* = 8.2 Hz, 12H), 8.08 (d, *J* = 4.7 Hz, 12H), 8.05 (d, *J* = 8.2 Hz, 12H), 7.95 (d, *J* = 1.9 Hz, 24H), 7.49 (t, *J* = 1.9 Hz, 12H), 6.71 (s, 12H), 3.82 (s, 18H), 1.21 (s, 216H).

^{13}C NMR (151 MHz, $CDCl_3$) δ 154.8, 150.7, 149.8 (two overlapping signals), 148.2, 142.8, 142.7, 141.9, 134.6, 134.1, 133.5, 131.93, 131.87, 131.7, 129.5, 124.2, 122.9, 120.8, 120.4, 119.5, 75.4, 52.4, 34.8, 31.5.

HRMS (MALDI) *m/z*: $[M]^+$ Calcd for $C_{348}H_{354}N_{24}O_6Zn_6$ 5360.3895; Found: 5358.4073.

UV/Vis (CH_2Cl_2, 298 K) λ, log ε: 420.5 (5.89), 453.5 (5.94), 559.5 (5.25), 601.5 (4.40).

<u>6b</u>

^{1}H NMR (600 MHz, $CDCl_3$) δ 9.02 (d, *J* = 4.7 Hz, 16H), 8.92 (d, *J* = 4.7 Hz, 16H), 8.60 (d, *J* = 4.7 Hz, 16H), 8.34 (d, *J* = 8.0 Hz, 16H), 8.06 (d, *J* = 8.0 Hz, 16H), 8.03 (d, *J* = 4.8 Hz, 16H), 7.96 (d, *J* = 1.8 Hz, 32H), 7.57 (t, *J* = 1.8 Hz, 16H), 6.68 (s, 16H), 3.82 (s, 24H), 1.30 (s, 288H).

^{13}C NMR (151 MHz, $CDCl_3$) δ 153.7, 149.8, 148.92, 148.89, 147.3, 141.8, 141.4, 140.5, 133.5, 133.1, 132.7, 131.1, 131.0, 130.8, 128.5, 123.3, 122.1, 120.1, 119.5, 118.4, 74.4, 51.4, 33.8, 30.5.

HRMS (MALDI) *m/z*: $[M]^+$ Calcd for $C_{464}H_{472}N_{32}O_8Zn_8$ 7148.1859; Found: 7148.1947.

UV/Vis (CH_2Cl_2, 298 K) λ, log ε: 419 (5.99), 454.5 (5.97), 559.5 (5.33), 599 (4.48).

<u>Aromatization of 5b and 6b to 7 and 8.</u> **5b** (8.0 mg, 1.5 µmol) was placed in a glass tube equipped with a J-Young cap together with a glass-coated stir bar and dried for 2 h under high vacuum. Pieces of lithium metal (freshly squeezed with tweezers to expose the metal surface) were added (around 30 mg, excess, ca. 3500 equiv.) The flask was evacuated and filled with argon. While under argon, dry and freshly degassed (via 3 x freeze-pump-thaw cycles) THF (2 mL) was added. The reaction vessel was then sealed and allowed to stir at room temperature overnight on 200 rpm rotation. After 16 h, stirring was increased to 800 rpm and reaction was monitored every hour until the color changed to deep brown and then a slightly greenish color appeared (Figure S1). Quenching the reaction too late when the color of the mixture turns completely green or black/grey causes complete decomposition of the material. Quenching the reaction too early means that part of the cyclohexadiene rings are not aromatized. After the desired change of color was observed, the J-Young seal was quickly opened and five drops of methanol were added. The solution was quickly (before much sodium reacts with methanol) transferred to a separate flask followed by the removal of solvent. The resulting solid was washed several times with methanol. The solid was redissolved in DCM (1 mL) and filtered through a pipette with a piece of wool inside to remove insoluble impurities and the solvent was evaporated which provided product as a brown solid. Yield: **7** (65%, 5.2 mg). No noticeable decomposition was noticed when solid samples of **7** were kept in a freezer for four months, however storing them in solution for a prolonged period of time (>2 days) should be avoided.

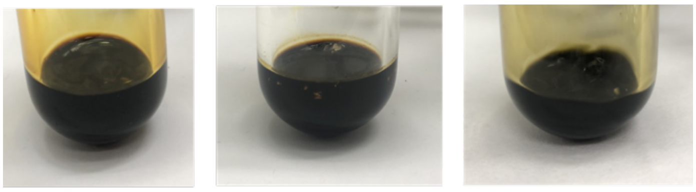

Figure S1. Progress of aromatization reaction. The picture on the left was taken at the beginning of the reaction. In the middle, reaction is close to completion but still requires time. On the right the reaction is finished and should be quenched as soon as possible.

6b (8.0 mg, 1.1 µmol) was placed in a glass tube equipped with a J-Young cap together with a glass-coated stir bar and dried for 2 h under high vacuum. Pieces of lithium metal (freshly squeezed with tweezers to expose the metal surface) were added (around 30 mg, excess, ca. 3500 equiv.) The flask was evacuated and filled with argon. While under argon, dry and freshly degassed (via 3 x freeze-pump-thaw cycles) THF (2 mL) was added. The reaction vessel was then sealed and allowed to stir at room temperature overnight on 200 rpm rotation. After 16 h, stirring was increased to 800 rpm and reaction was monitored every hour until the color changed to deep brown and then a slightly greenish color appeared (Figure S1). Quenching the reaction too late when the color of the mixture turns completely green or black/grey causes complete decomposition of the material. Quenching the reaction too early means that part of the cyclohexadiene rings are not aromatized. After the desired change of color was observed, the J-Young seal was quickly opened and five drops of methanol were added. The solution was quickly (before much sodium reacts with methanol) transferred to a separate flask followed by the removal of solvent. The resulting solid was washed several times with methanol. The solid was redissolved in DCM (1 mL) and filtered through a pipette with a piece of wool inside to remove insoluble impurities and the solvent was evaporated which provided product as a brown solid. Yield of **8** (4.8 mg, 60%). No noticeable decomposition was noticed when solid samples were kept in a freezer for four months, however storing them in solution for a prolonged period of time (>2 days) should be avoided.

7

^{1}H NMR (400 MHz, $CDCl_3$) δ 9.06 (d, *J* = 4.7 Hz, 12H), 8.97 (d, *J* = 4.7 Hz, 12H), 8.62 (d, *J* = 4.8 Hz, 12H), 8.17 (d, *J* = 7.9 Hz, 12H), 8.06 (s, 12H), 8.04 (d, *J* = 4.8 Hz, 12H), 8.00 (d, *J* = 1.9 Hz, 24H), 7.98 (d, overlapping with doublet at 8.00, 12H), 7.64 (t, *J* = 1.8 Hz, 12H), 1.38 (s, 216H).

^{13}C NMR (151 MHz, $CDCl_3$) δ 154.0, 150.5, 149.78, 149.75, 148.5, 141.7, 141.5, 139.96, 139.87, 134.8, 133.6, 132.5, 131.9, 129.5, 128.1, 125.4, 123.3, 120.8 (two overlapping peaks), 120.7, 118.8, 34.9, 31.6.

HRMS (MALDI) *m/z*: $[M]^+$ Calcd for $C_{342}H_{336}N_{24}Zn_6$ 5174.2788; Found: 5177.3552.

UV/Vis (CH_2Cl_2, 298 K) λ, log ε: 420.5 (5.76), 460 (5.69), 562 (5.06).

8

^{1}H NMR (400 MHz, $CDCl_3$ + 1% pyr-d_5) δ 8.98 (d, *J* = 4.6 Hz, 16H), 8.91 (d, *J* = 4.6 Hz, 16H), 8.48 (d, *J* = 4.8 Hz, 16H), 8.23 (d, *J* = 8.2 Hz, 16H), 8.09 (s, 16H), 8.01 (d, *J* = 8.2 Hz, 16H), 7.98 (d, *J* = 1.9 Hz, 32H), 7.82 (d, *J* = 4.8 Hz, 16H), 7.63 (t, *J* = 1.9 Hz, 32H), 1.40 (s, 288 H).

^{13}C NMR (151 MHz, $CDCl_3$ + 1% pyr-d_5) δ 154.4, 150.4, 149.7, 148.1, 142.8, 142.3, 140.1, 139.4, 135.7, 134.9, 133.4, 131.9, 131.7, 131.3, 129.6, 128.0, 125.1, 123.4, 122.6, 120.3, 119.4, 34.9, 31.7.

HRMS (MALDI) *m/z*: $[M]^+$ Calcd for $C_{456}H_{448}N_{32}Zn_8$ 6899.0386; Found: 6902.8487.

UV/Vis (CH_2Cl_2, 298 K) λ, log ε: 420 (5.89), 461 (5.84), 562 (5.29).

Fusion of 7 to 9. 7 (10 mg, 1.9 µmol) was placed in a Schlenk flask together with DDQ (5.3 mg, 23 µmol, 12 equiv.) and $Sc(OTf)_3$ (14.8 mg, 30 µmol, 15.6 equiv.) and the atmosphere was changed to argon. Dry toluene (3.2 mL) was added under argon and the mixture was stirred for 1.5 h in an oil bath at 80 °C. A color change from brown to blue/green was observed. The reaction was cooled to room temperature and then quenched by addition of triethylamine (100 µL, 0.72 mmol). The solvent was evaporated with rotary evaporator and the product was recrystallized from methanol, redissolved in dichloromethane (1.5 mL) and filtered through cotton wool. Evaporation of solvent provided compound **9** as a violet/blue solid. Yield: 7.0 mg (70%). Under identical conditions, fusion of **8** was unsuccessful (See: Section 4f).

^{1}H NMR (600 MHz, $CDCl_3$) δ 7.88 (d, *J* = 4.6 Hz, 12H), 7.79 (d, *J* = 8.1 Hz, 12H), 7.74 (s, 12H), 7.64 (d, *J* = 8.1 Hz, 12H), 7.61 (d, *J* = 4.6 Hz, 12H), 7.56 (t, *J* = 1.8 Hz, 12H), 7.54 (d, *J* = 1.8 Hz, 24H), 7.06 (s, 12H), 1.40 (s, 216H).

^{13}C NMR (151 MHz, $CDCl_3$) δ 153.7, 153.2, 152.6, 148.8, 139.8, 135.6, 133.9, 131.4, 130.6, 128.2, 128.0, 127.5, 126.5, 125.2, 120.9, 116.5, 34.9, 31.6. 18 out of expected 23 carbons are visible due to low quality of the spectrum resulting from low solubility and aggregation, even though a long experiment on an NMR instrument equipped with a cryoprobe was used. We report HSQC and HMBC spectra in Figures S65 and S66.

HRMS (MALDI) *m/z*: $[M]^+$ Calcd for $C_{342}H_{324}N_{24}Zn_6$ 5162.1849; Found: 5162.8419.

UV/Vis (CH_2Cl_2, 298 K) λ, log ε: 420.5 (5.06), 587.5 (4.99), 946 (4.28), 1059 (4.45).

3. NMR and Mass Spectra

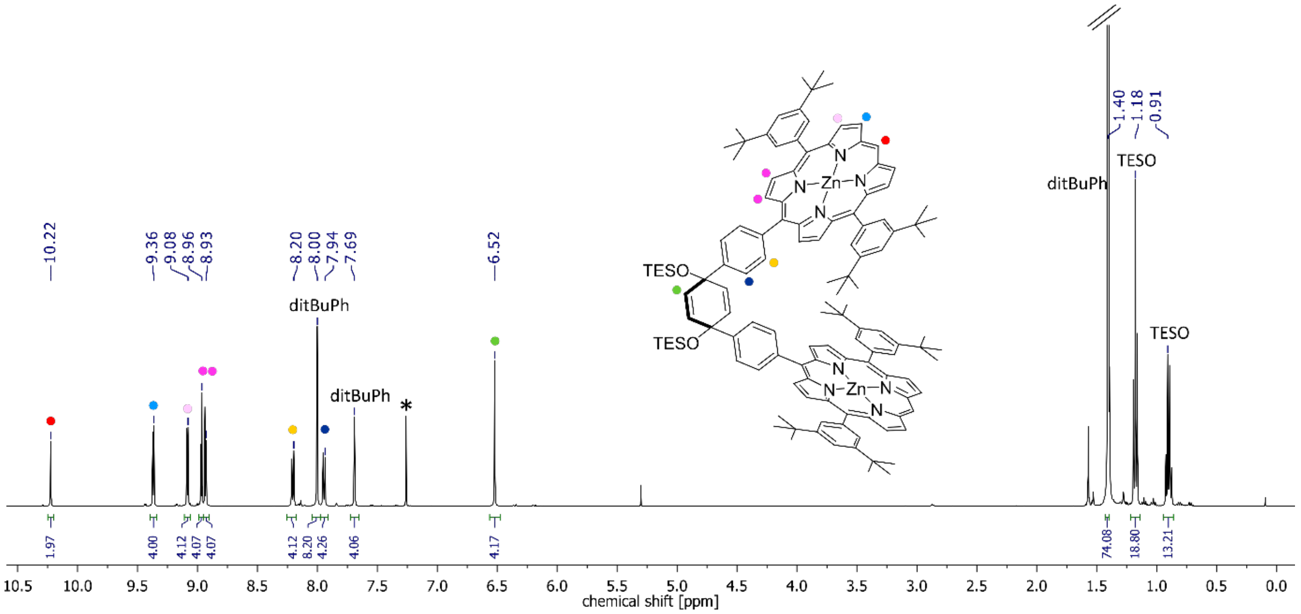

Figure S2. ^{1}H NMR spectrum of **3a**, $CDCl_3$, 600 MHz, 300 K.

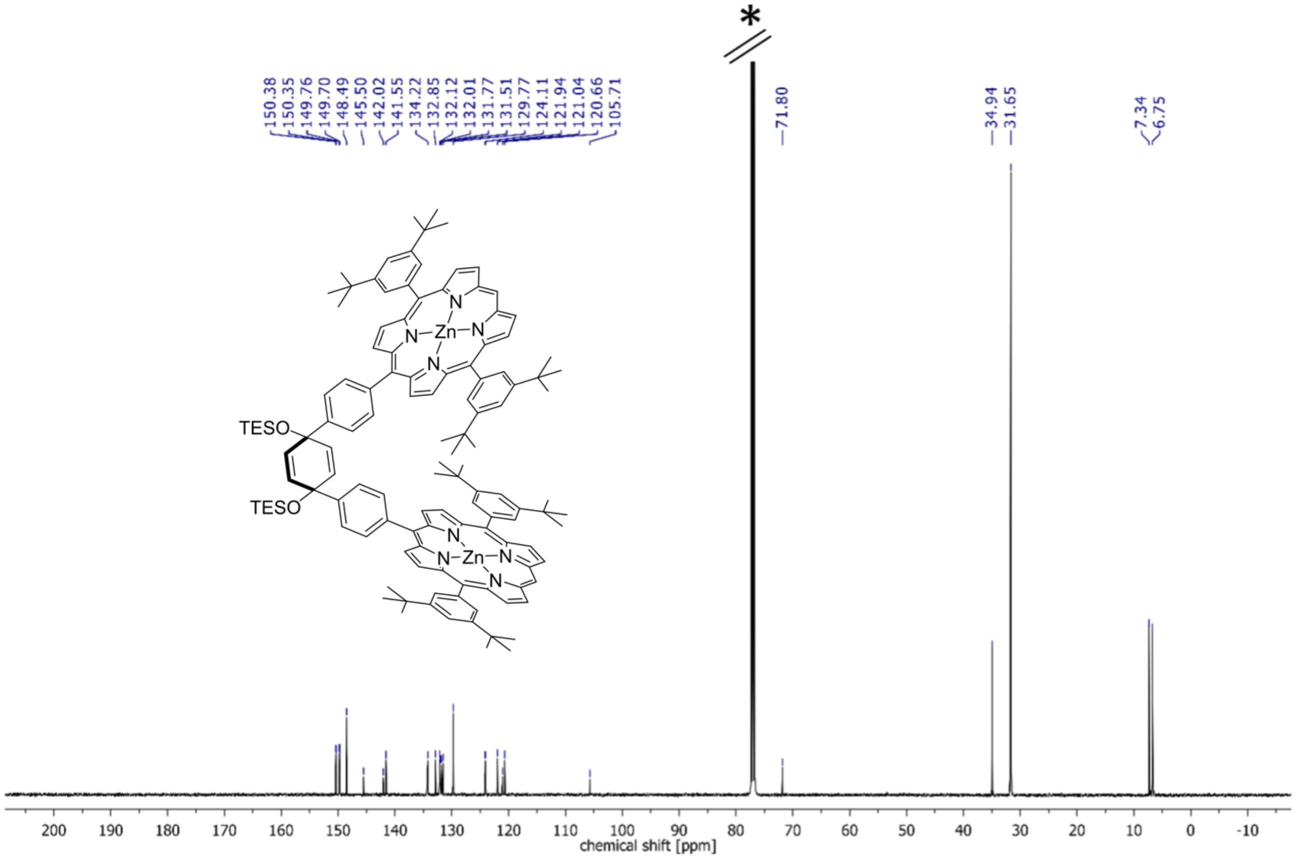

Figure S3. ^{13}C NMR spectrum of **3a**, $CDCl_3$, 126 MHz, 300 K.

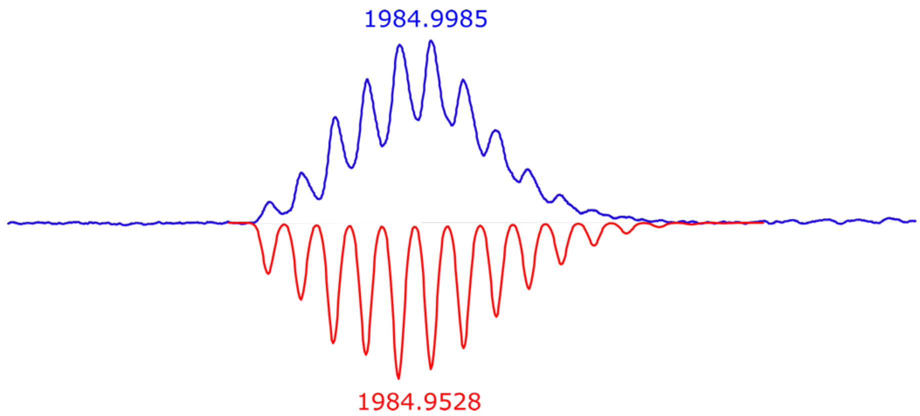

Figure S4. Selected region of a MALDI mass spectrum of **3a** together with simulated isotopic pattern (in red), $[M]^{+}$.

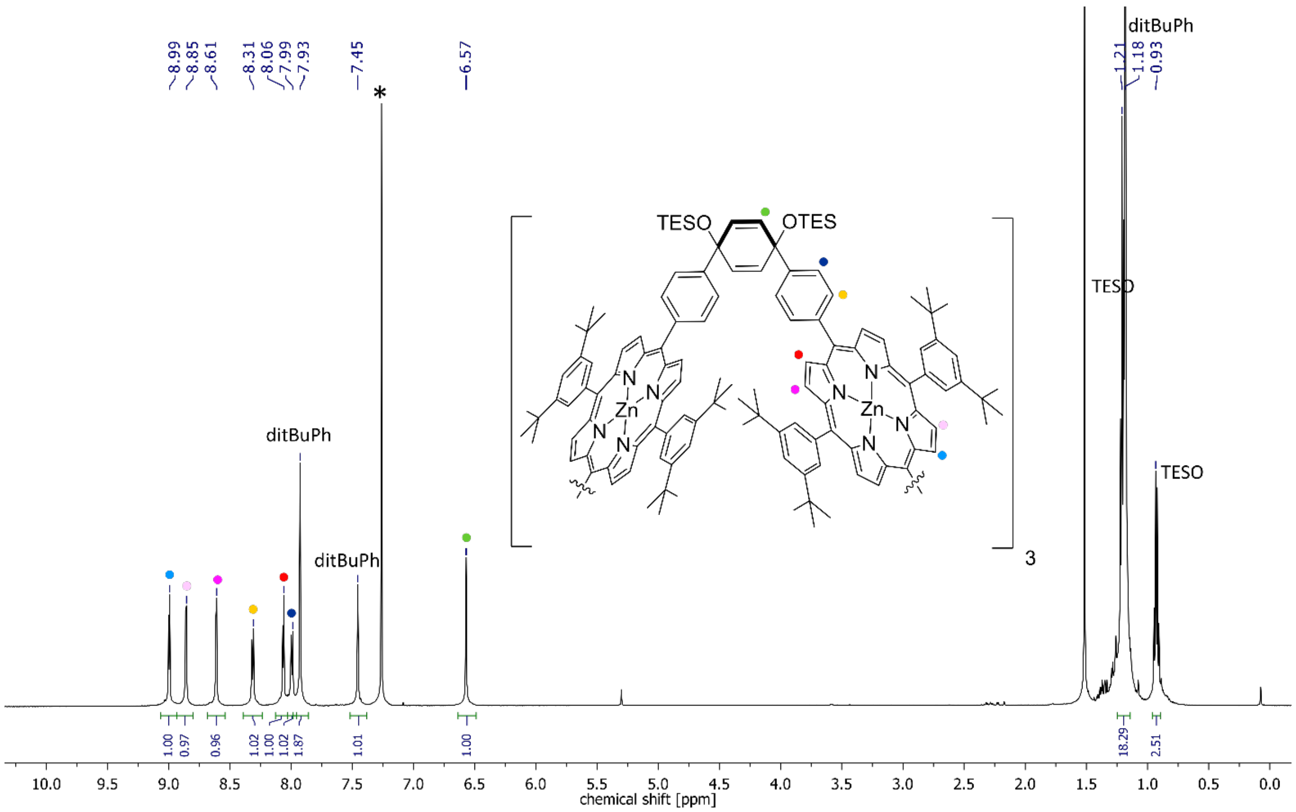

Figure S5. ^{1}H NMR spectrum of **5a**, $CDCl_3$, 600 MHz, 300 K.

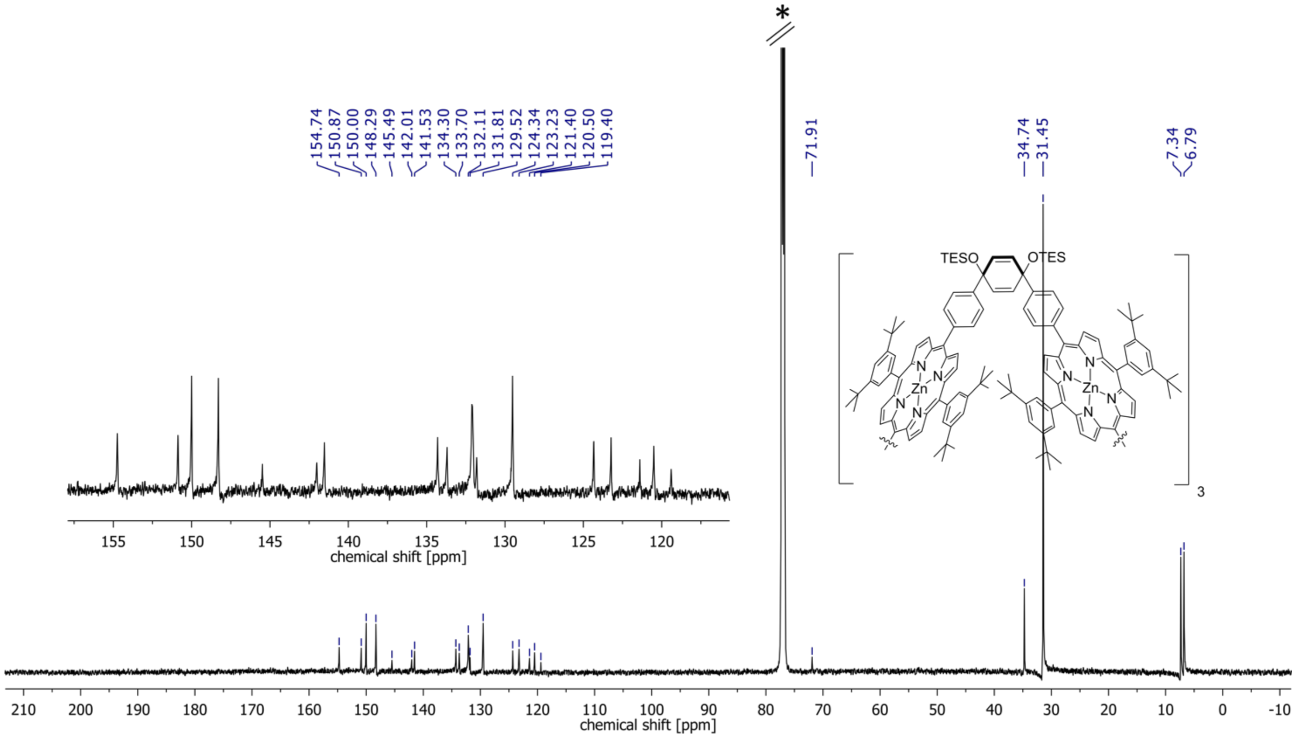

Figure S6. ^{13}C NMR spectrum of **5a**, $CDCl_3$, 151 MHz, 300 K.

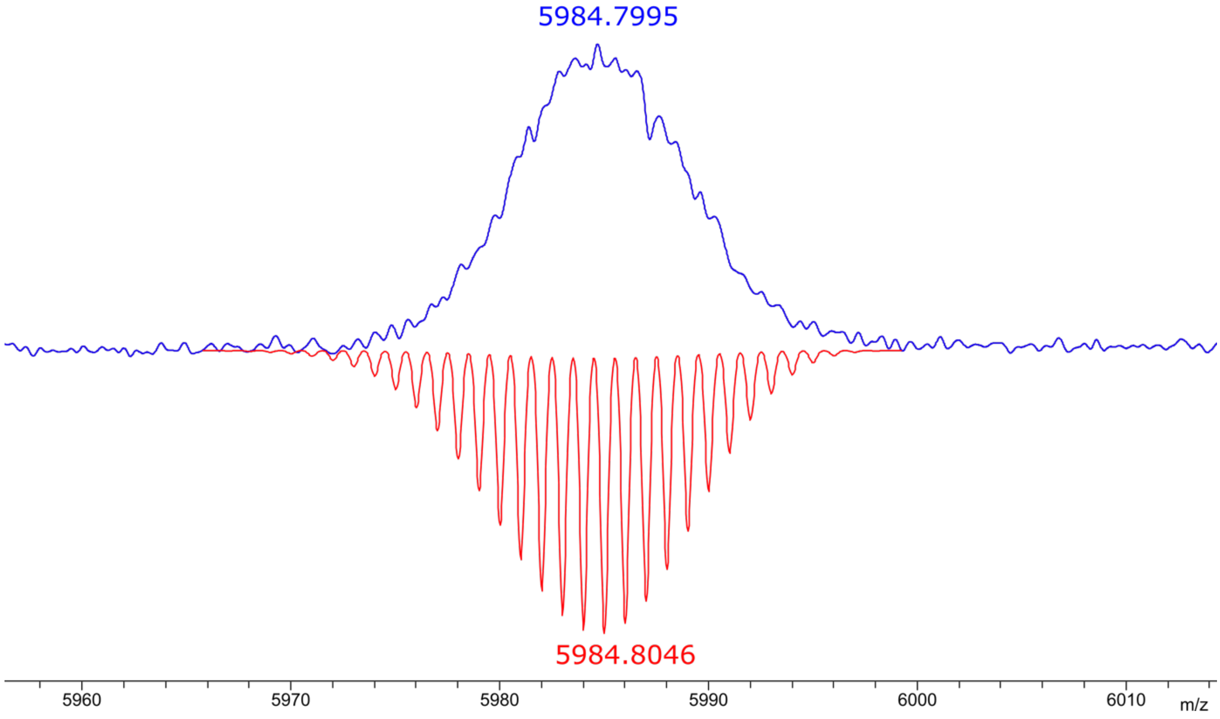

Figure S7. Selected region of a MALDI mass spectrum of **5a** together with simulated isotopic pattern (in red), $[M+Na]^+$.

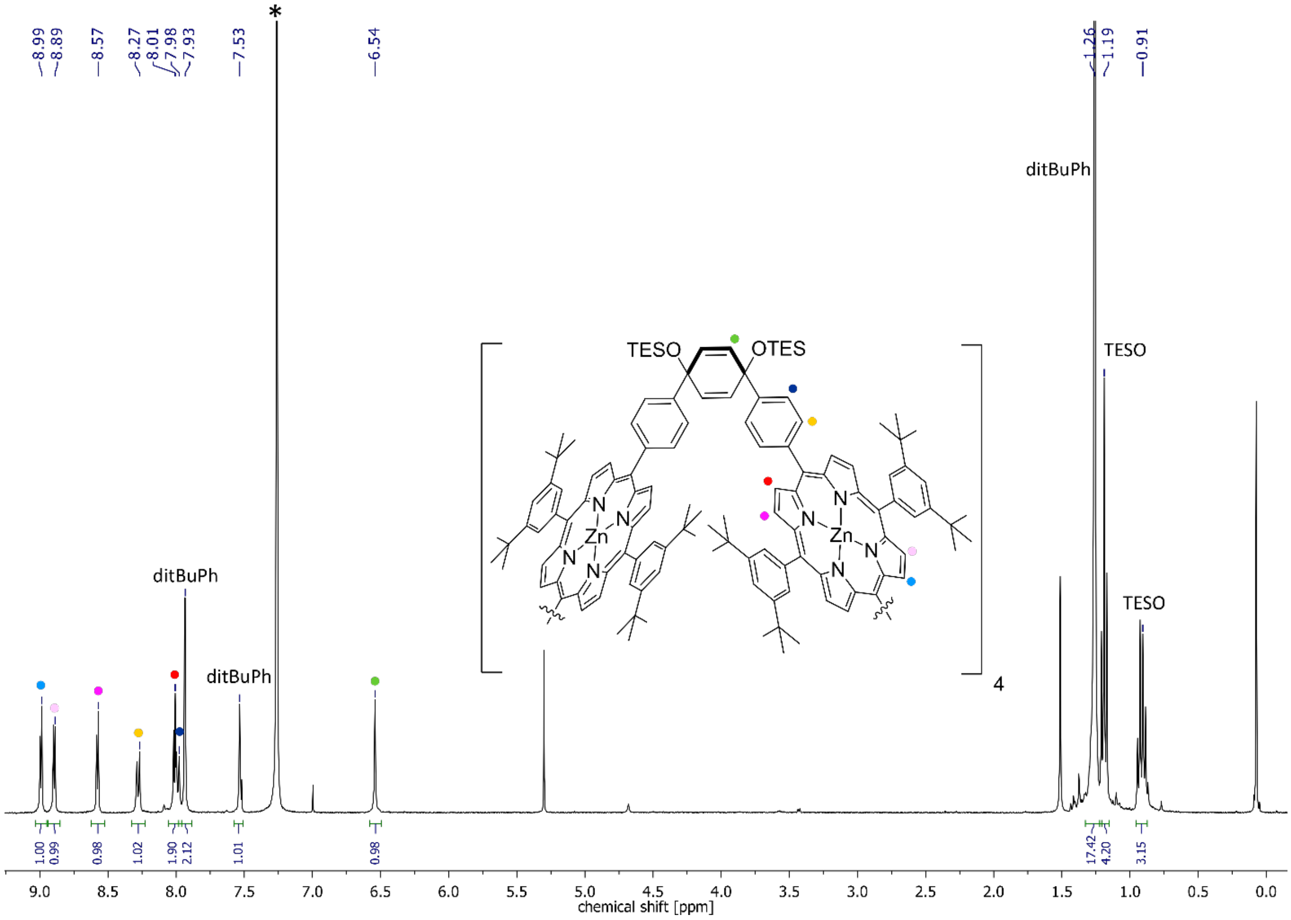

Figure S8. ^{1}H NMR spectrum of **6a**, $CDCl_3$, 400 MHz, 300 K.

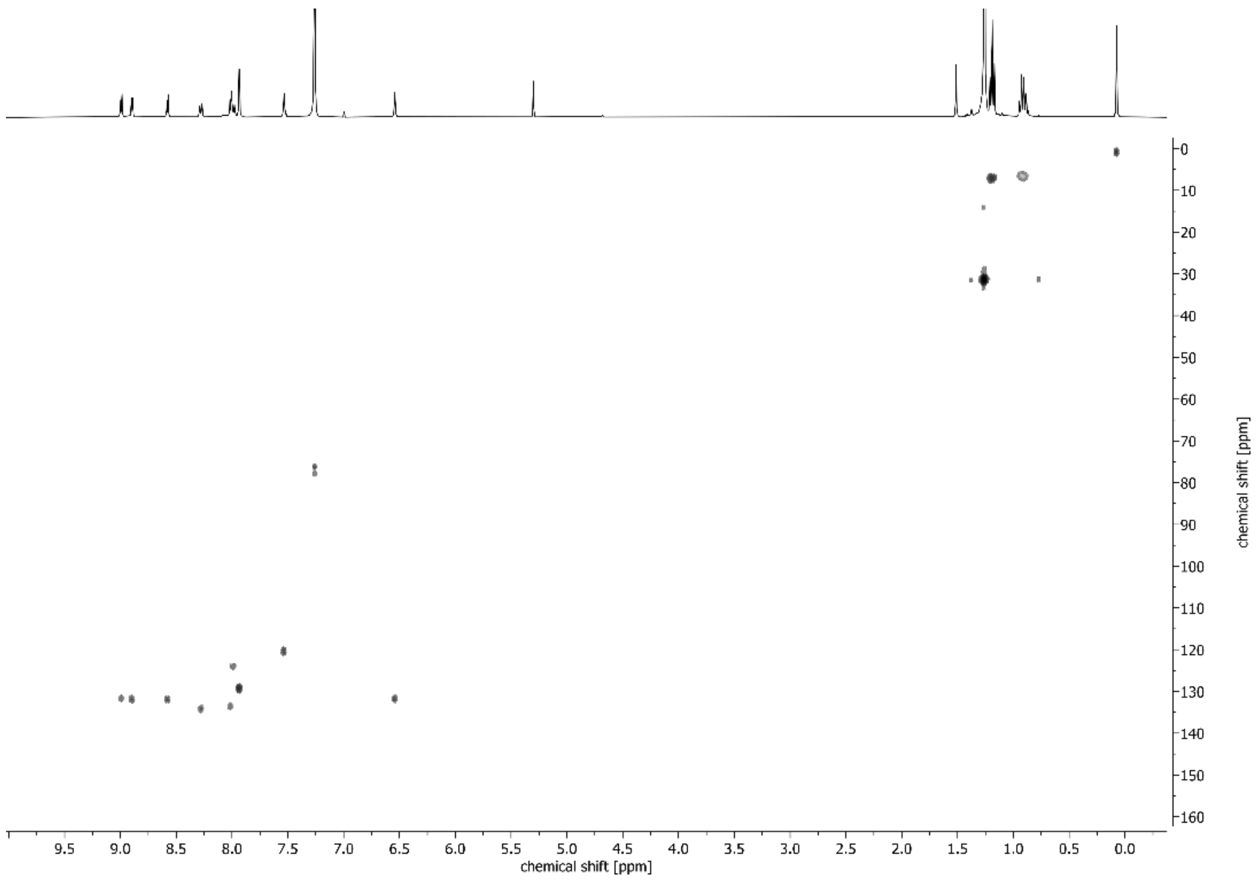

Figure S9. ^{1}H-^{13}C HSQC NMR spectrum of **6a**, $CDCl_3$, 400 MHz, 300 K.

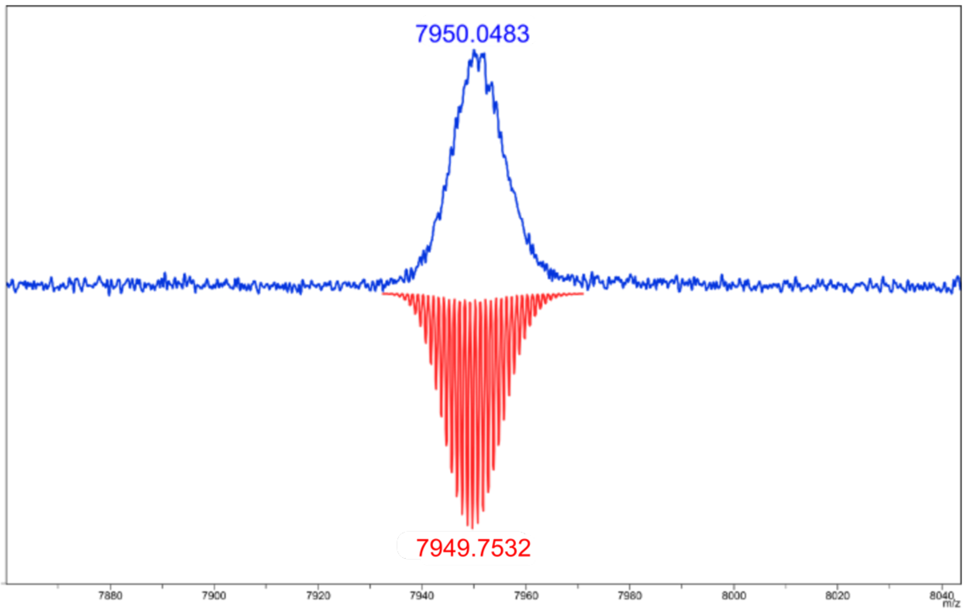

Figure S10. Selected region of a MALDI mass spectrum of **6a** together with simulated isotopic pattern (in red), [M]$^+$.

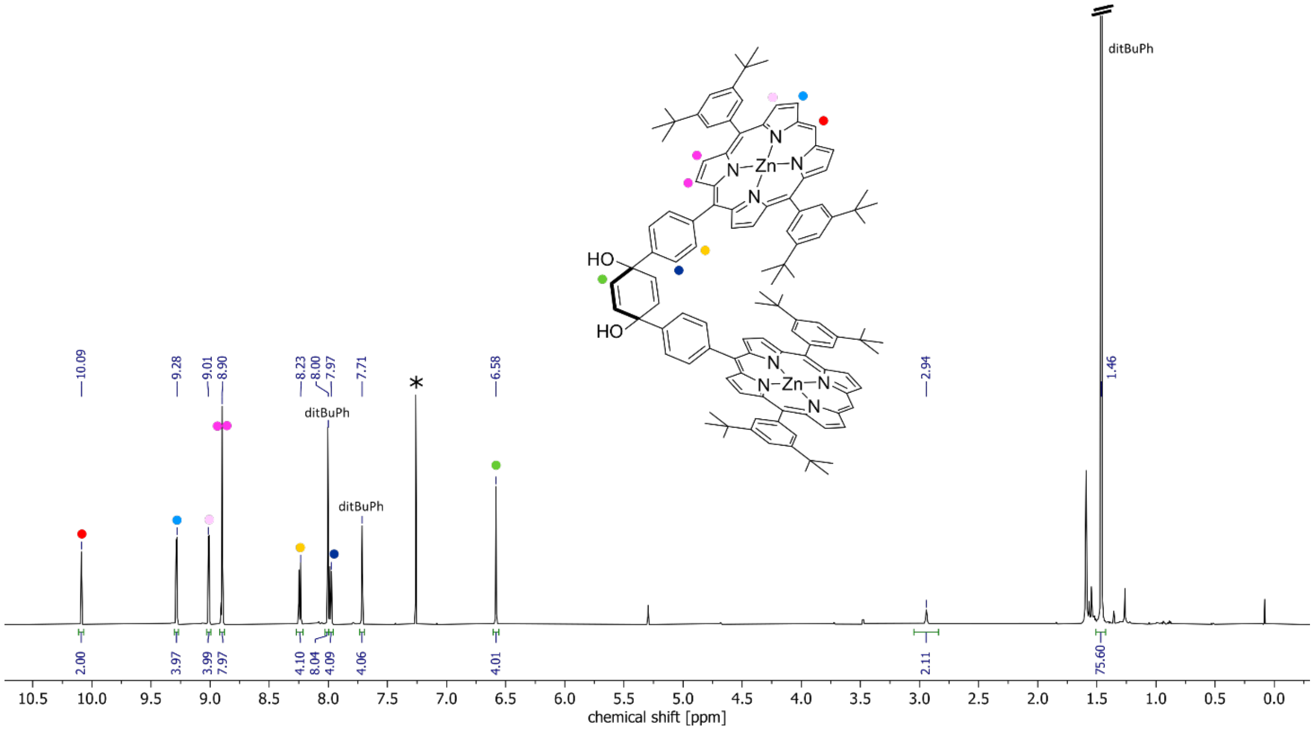

Figure S11. ^1H NMR spectrum of **3c**, $CDCl_3$ + 1% pyr-d_5, 600 MHz, 300 K.

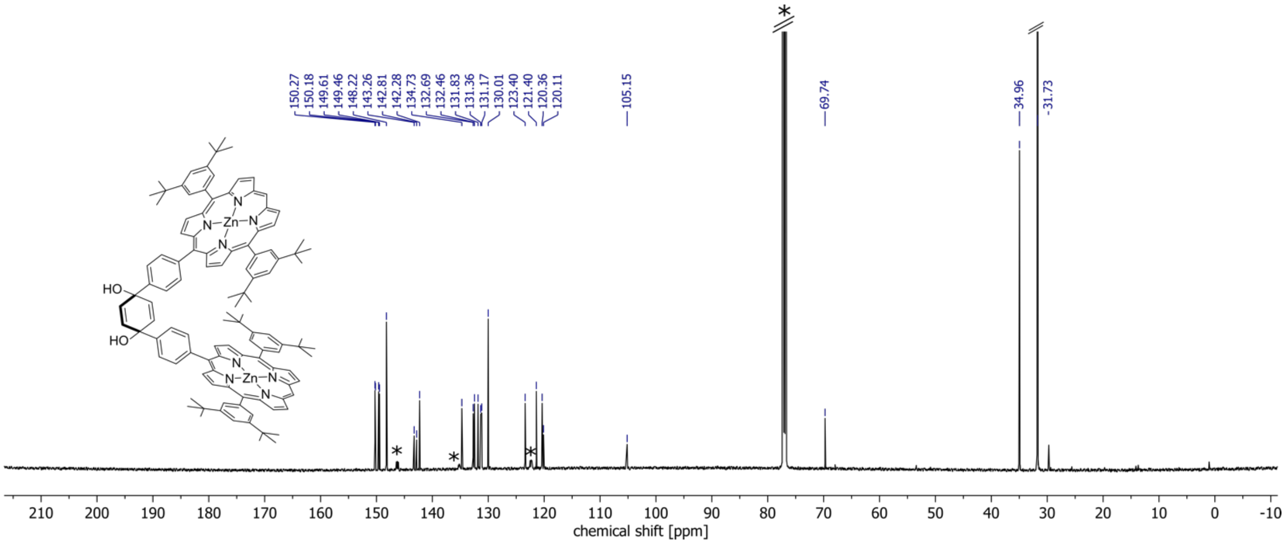

Figure S12. ^{13}C NMR spectrum of **3c**, $CDCl_3$ + 1% pyr-d_5, 151 MHz, 300 K.

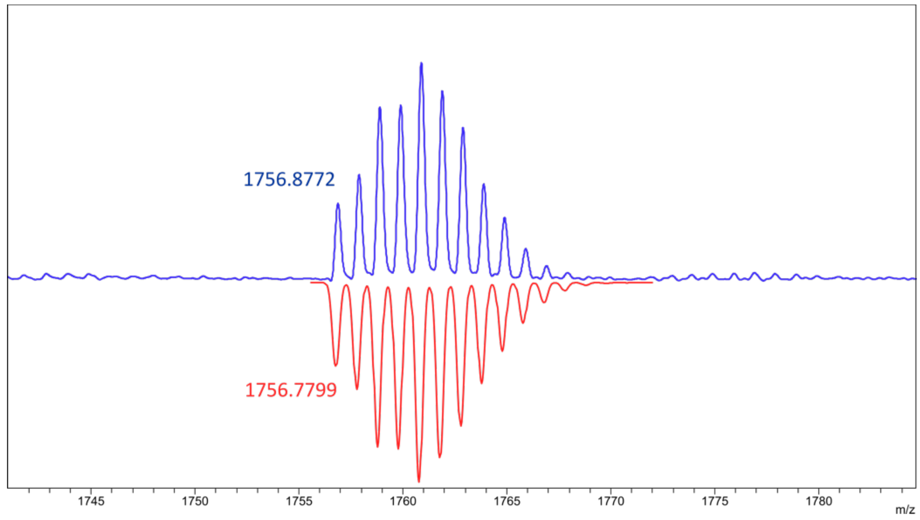

Figure S13. Selected region of a MALDI mass spectrum of **3c** together with simulated isotopic pattern (in red), $[M]^+$.

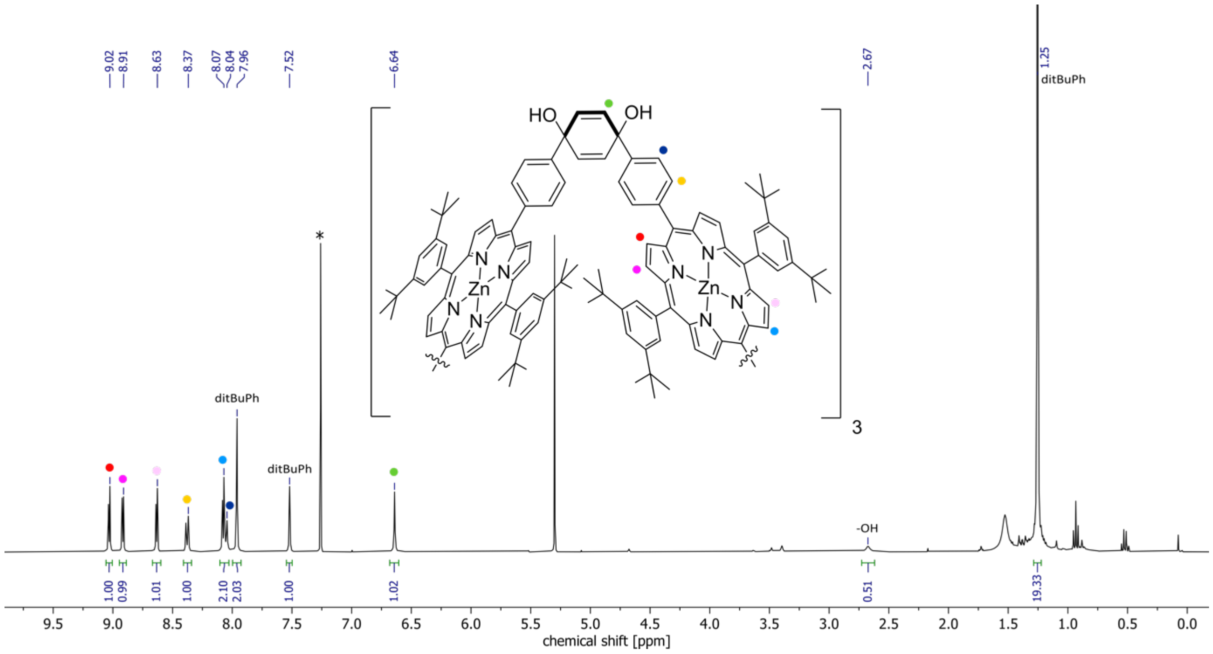

Figure S14. ^{1}H NMR spectrum of **5c**, $CDCl_3$, 400 MHz, 300 K.

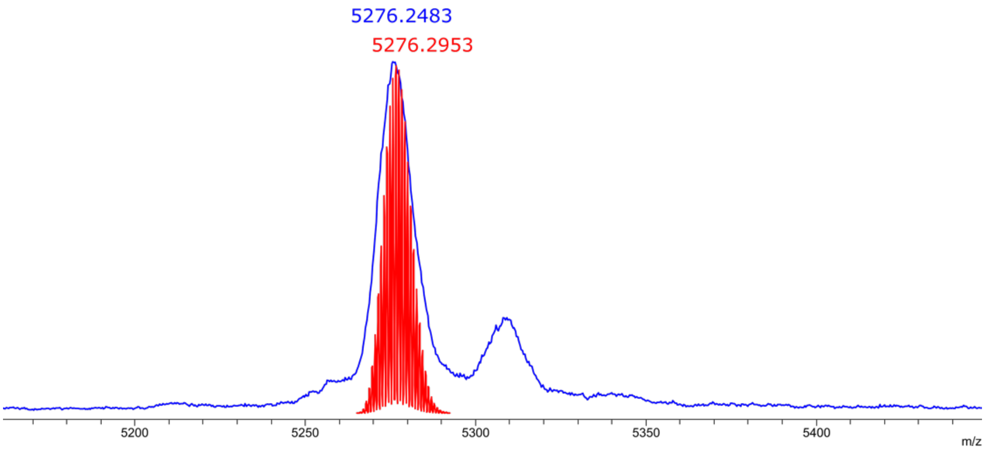

Figure S15. Selected region of a MALDI mass spectrum of **5c** together with simulated isotopic pattern (in red), [M]+.

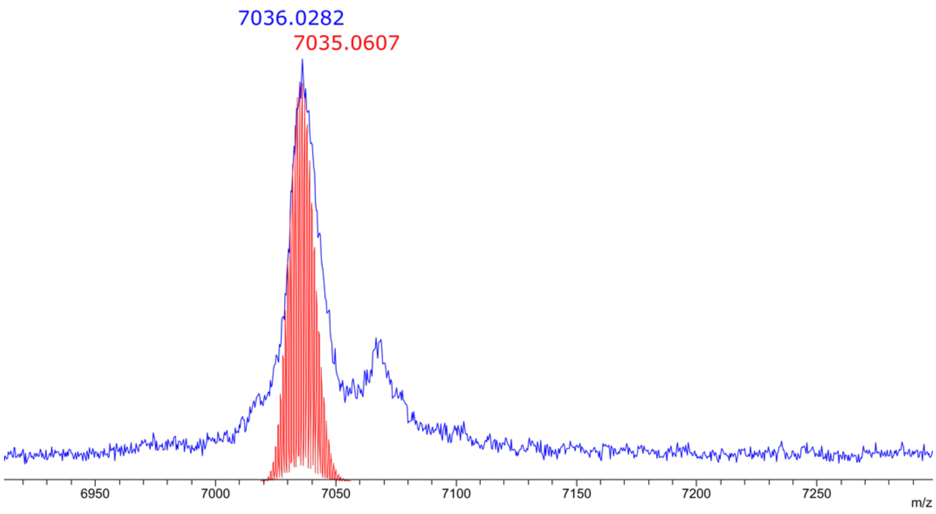

Figure S16. Selected region of a MALDI mass spectrum of **6c** together with simulated isotopic pattern (in red), [M]+.

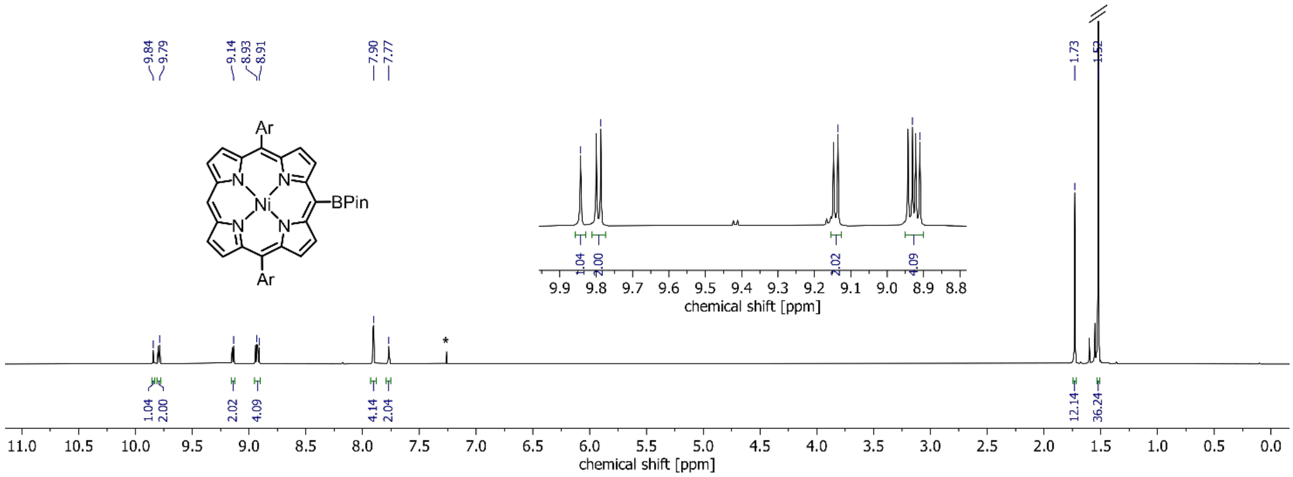

Figure S17. ^{1}H NMR spectrum of **2Ni**, $CDCl_3$, 400 MHz, 300 K.

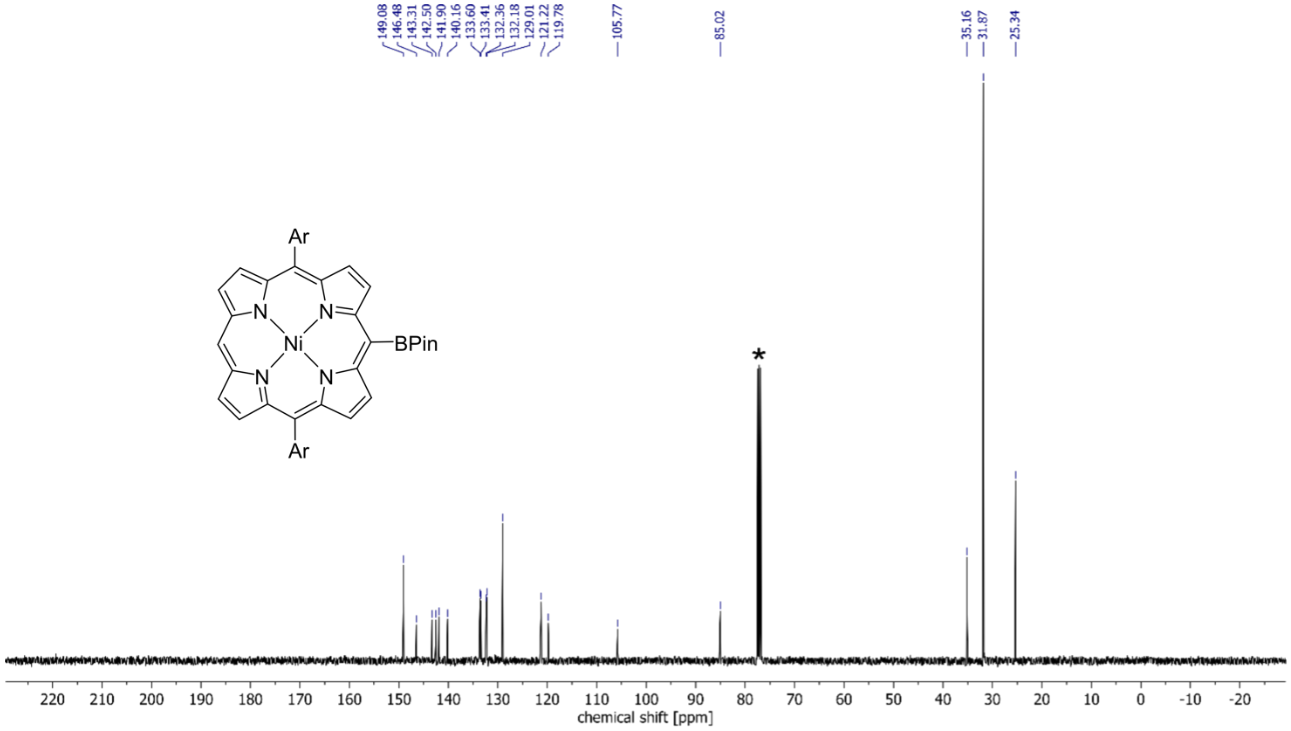

Figure S18. ^{13}C NMR spectrum of **2Ni**, $CDCl_3$, 101 MHz, 300 K.

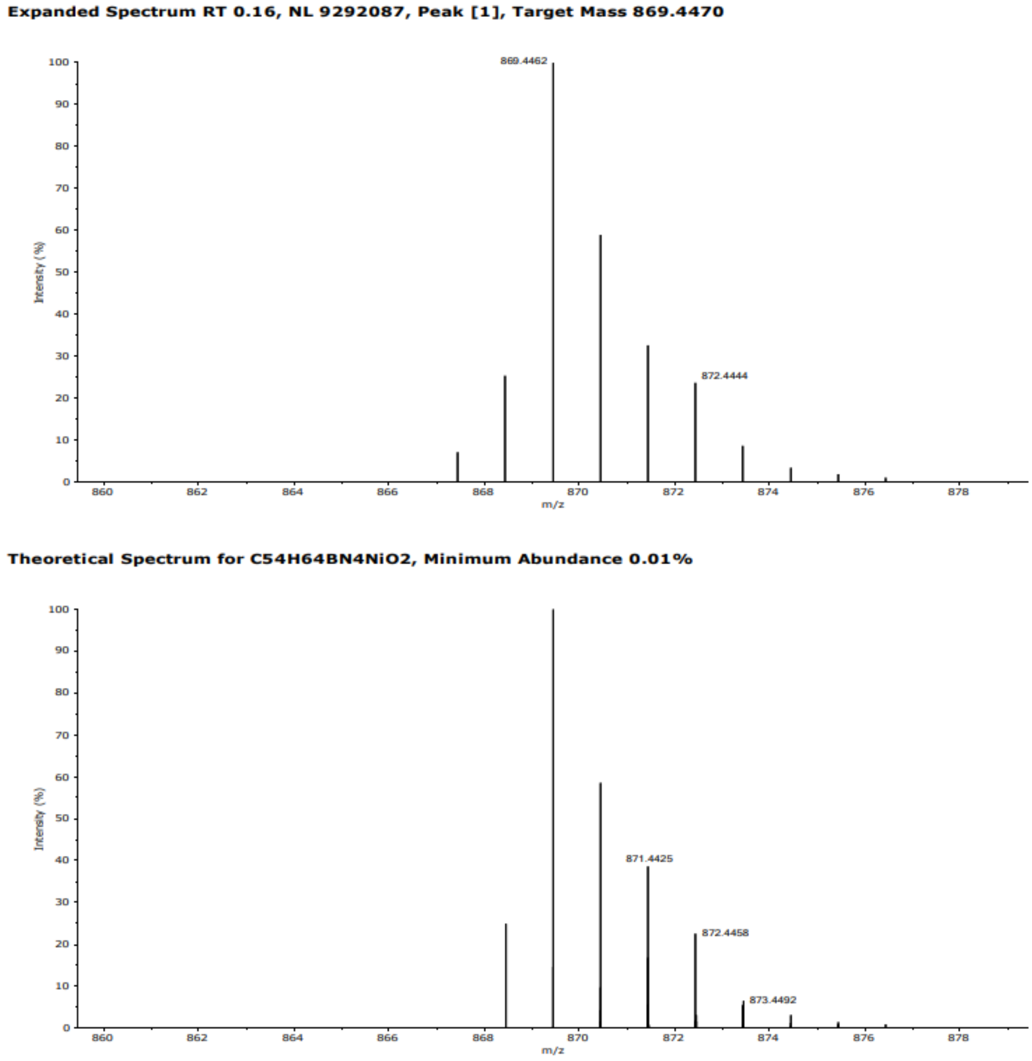

Figure S19. Selected region of a mass spectrum (ESI) of **2Ni** together with simulated isotopic pattern, $[M+H]^+$.

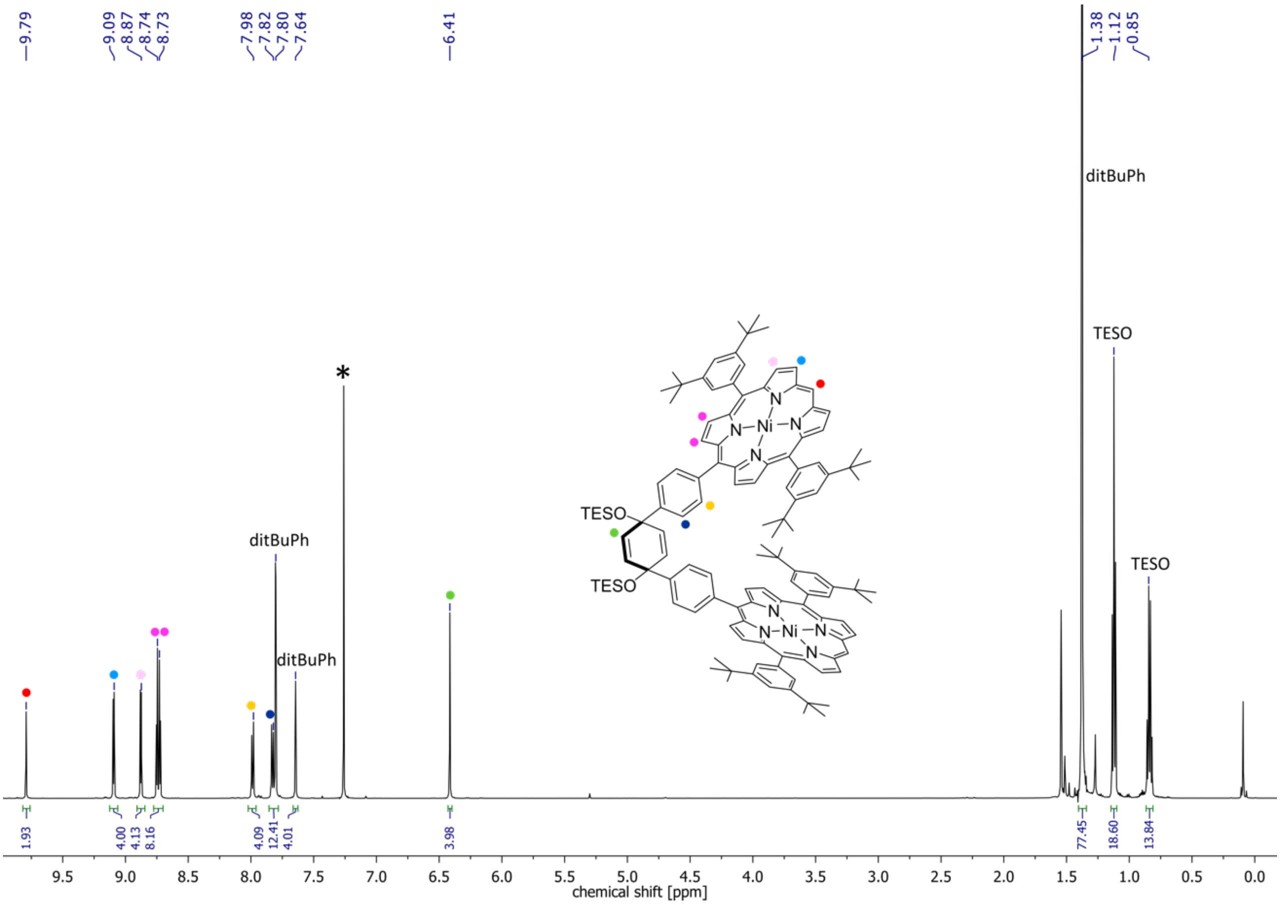

Figure S20. ^{1}H NMR spectrum of **3aNi**, $CDCl_3$, 600 MHz, 300 K.

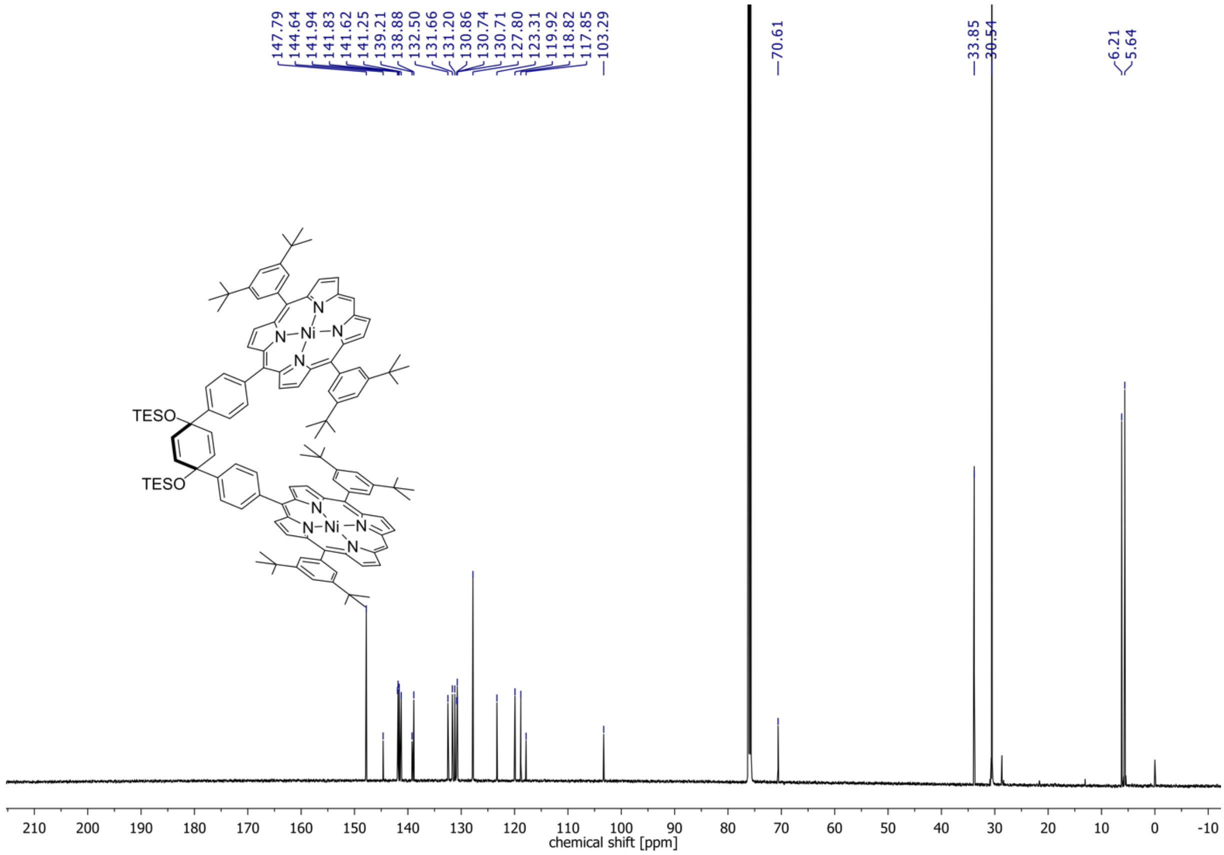

Figure S21. ^{13}C NMR spectrum of **3aNi**, $CDCl_3$, 151 MHz, 300 K.

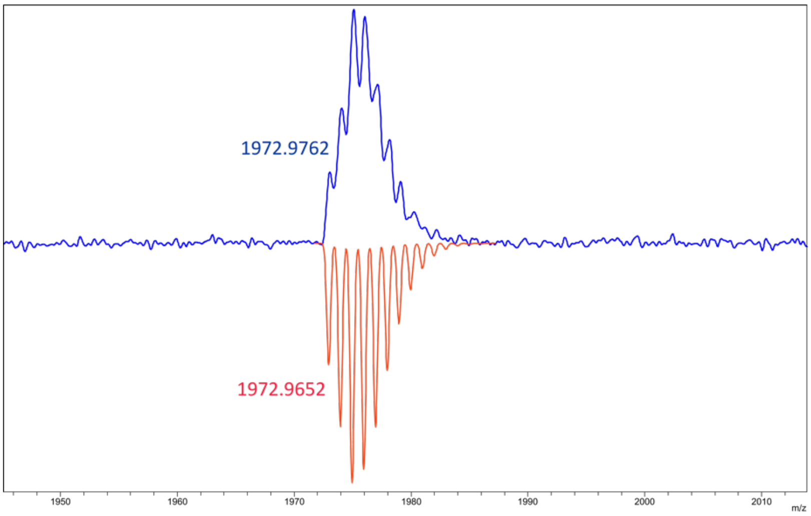

Figure S22. Selected region of a MALDI mass spectrum of **3aNi** together with simulated isotopic pattern (in red), $[M]^+$.

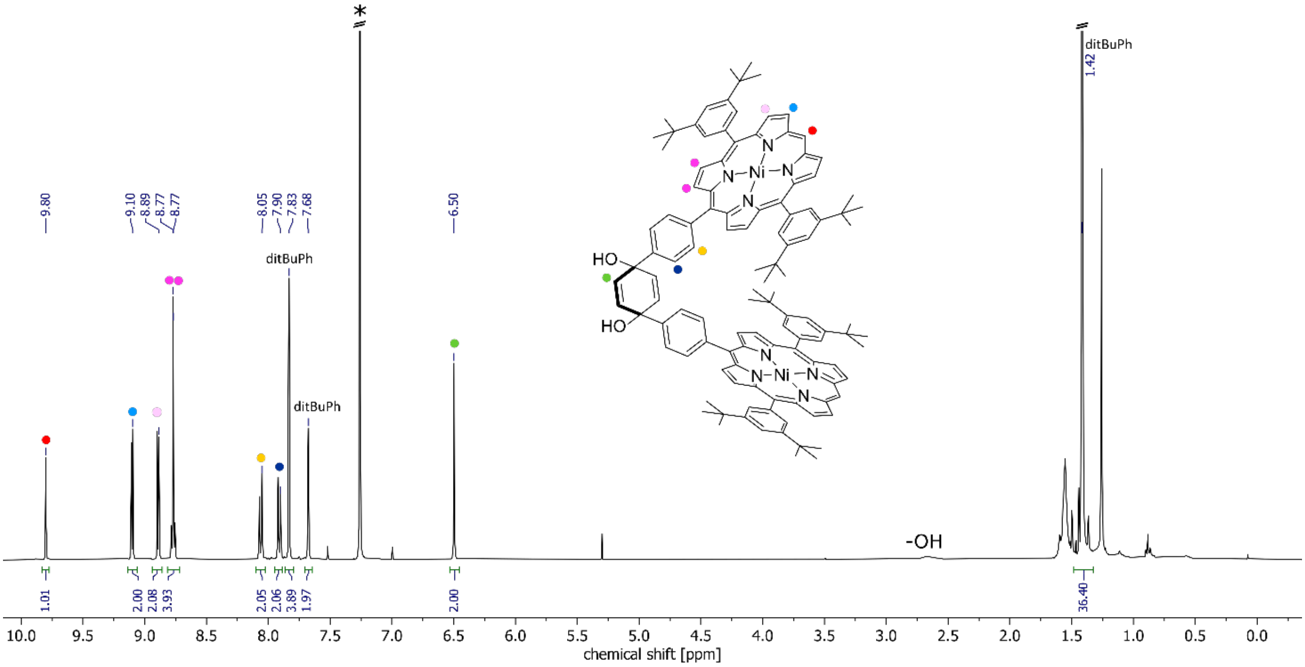

Figure S23. ^1H NMR spectrum of **3cNi** in $CDCl_3$ (600 MHz, 300 K).

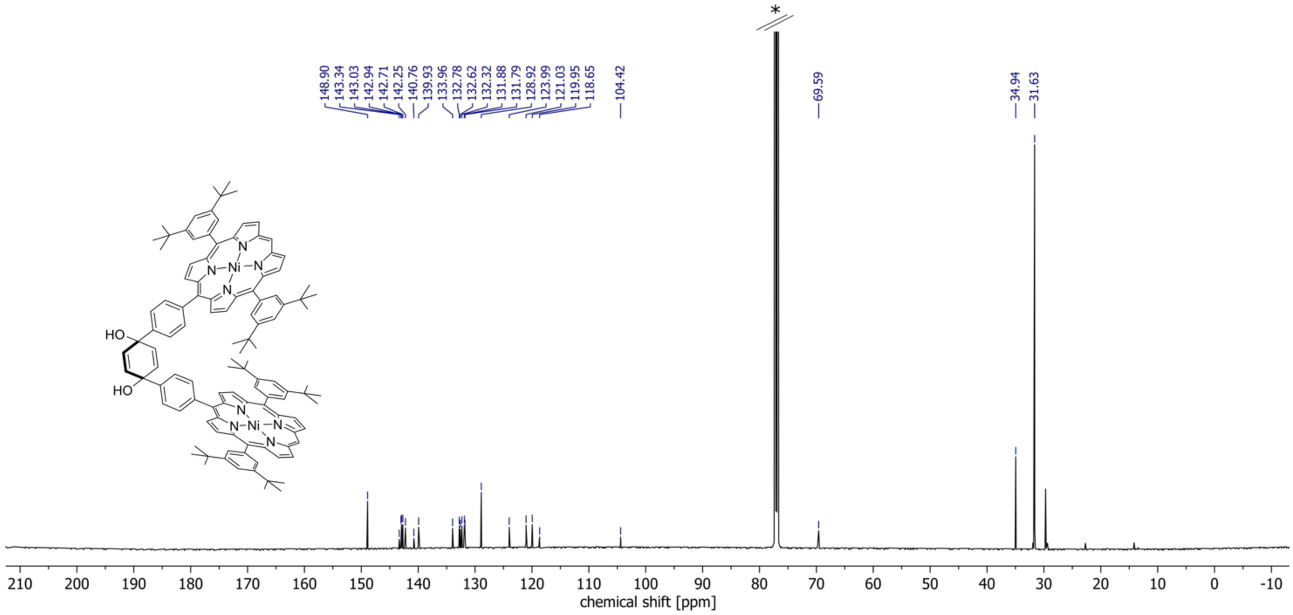

Figure S24. ^{13}C NMR spectrum of **3cNi**, $CDCl_3$, 151 MHz, 300 K.

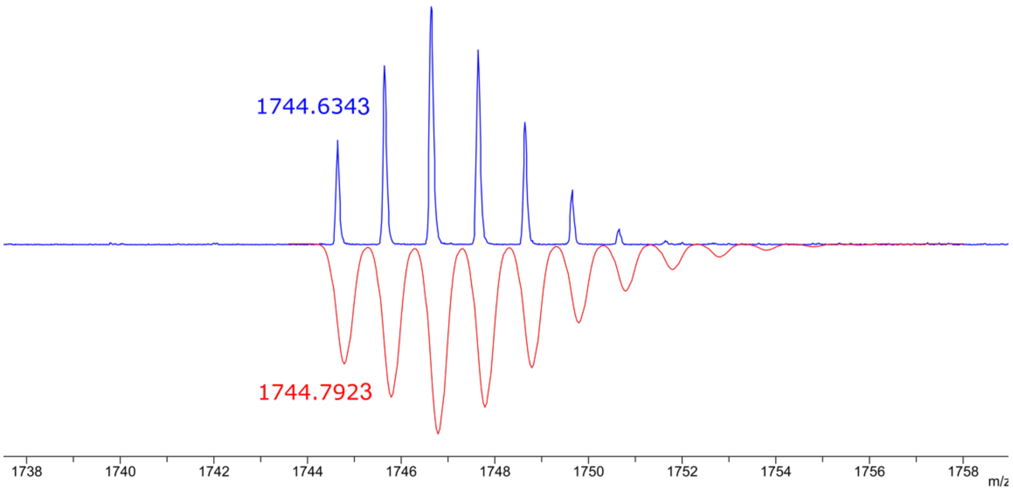

Figure S25. Selected region of a MALDI mass spectrum of **3cNi** together with simulated isotopic pattern (in red), $[M]^+$.

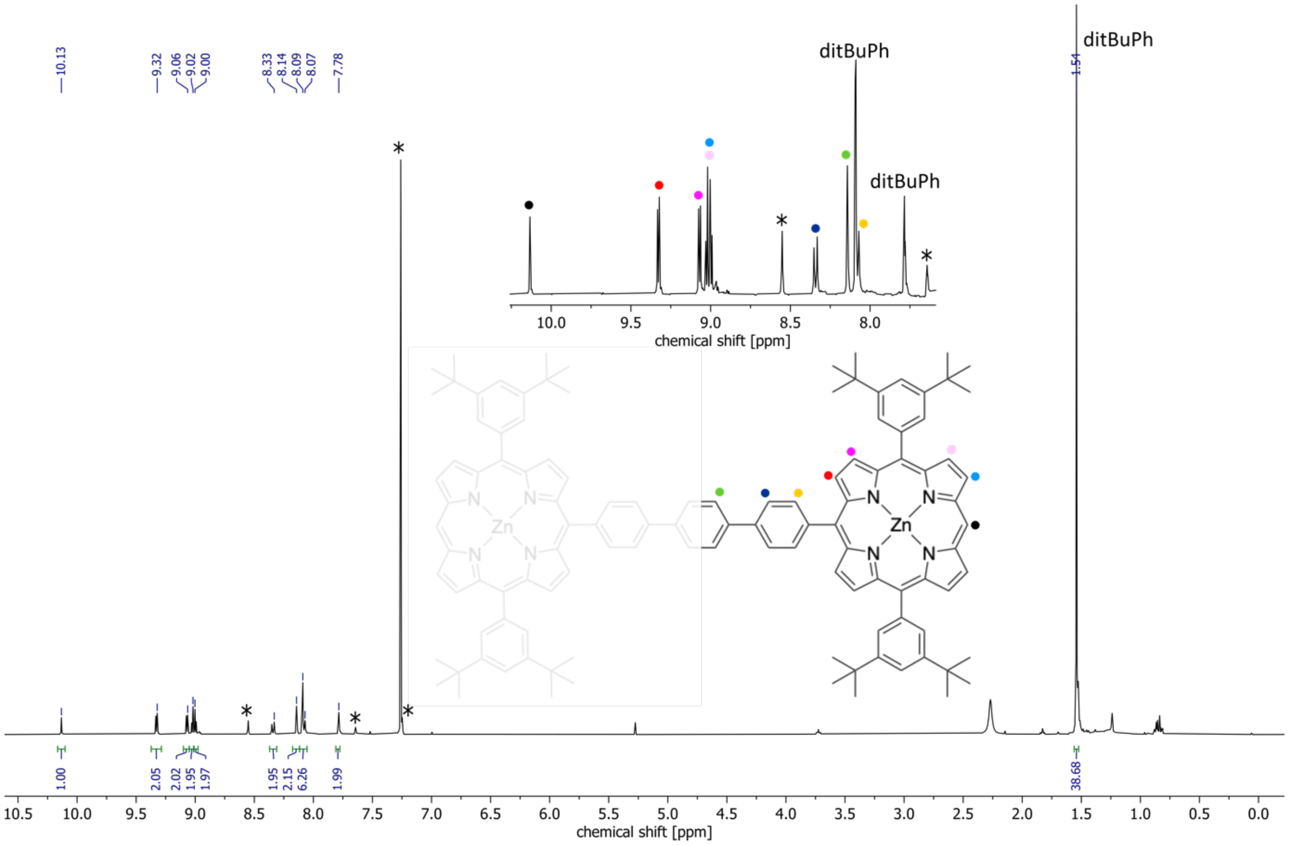

Figure S26. ^{1}H NMR spectrum of **4** in $CDCl_3$ + 1% pyr-d_5, (600 MHz, 300 K).

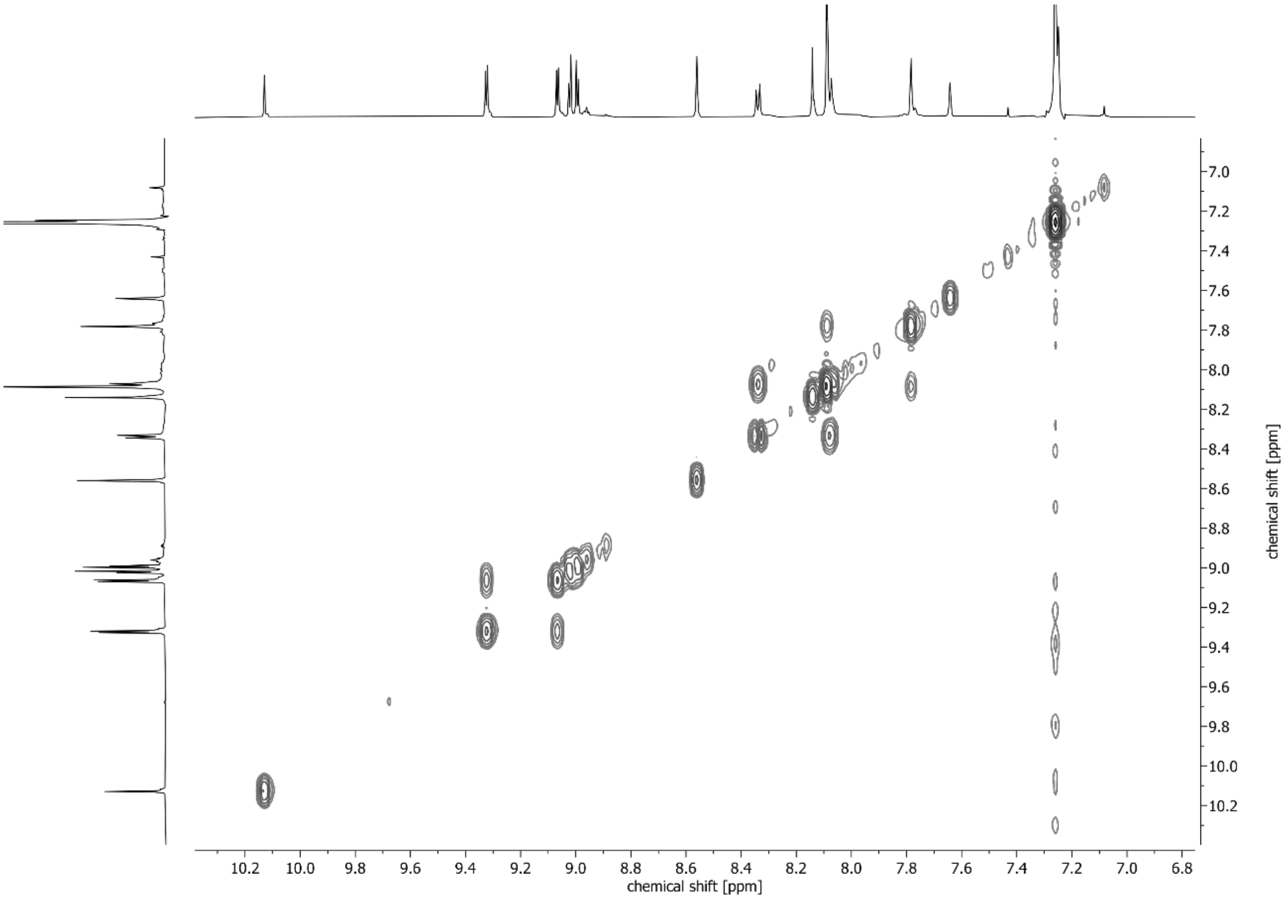

Figure S27. ^{1}H-^{1}H COSY NMR spectrum of **4** in $CDCl_3$ + 1% pyr-d_5, (600 MHz, 300 K).

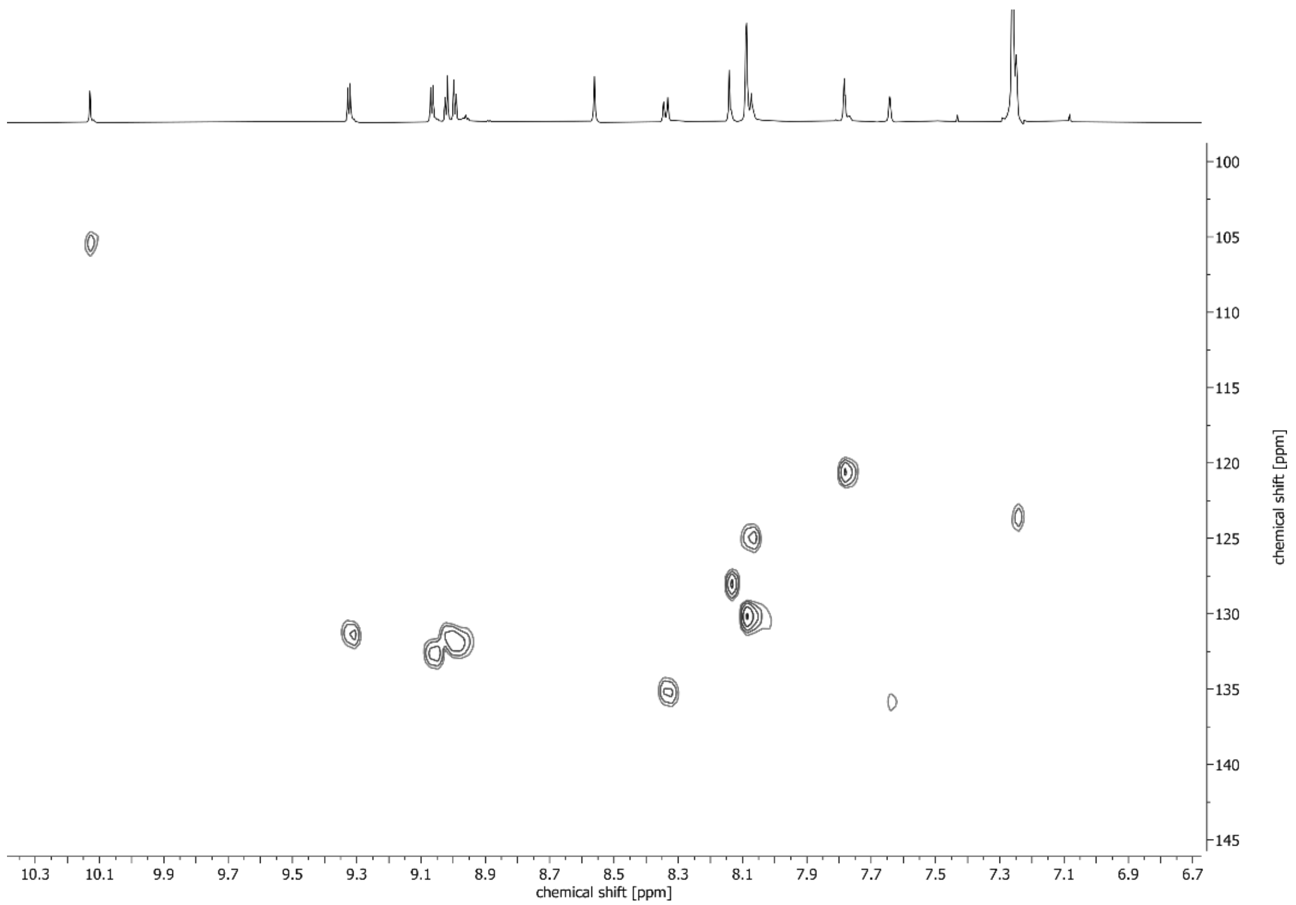

Figure S28. ^{1}H-^{13}C HSQC NMR spectrum of **4** in $CDCl_3$ + 1% pyr-d_5, (600 MHz, 300 K).

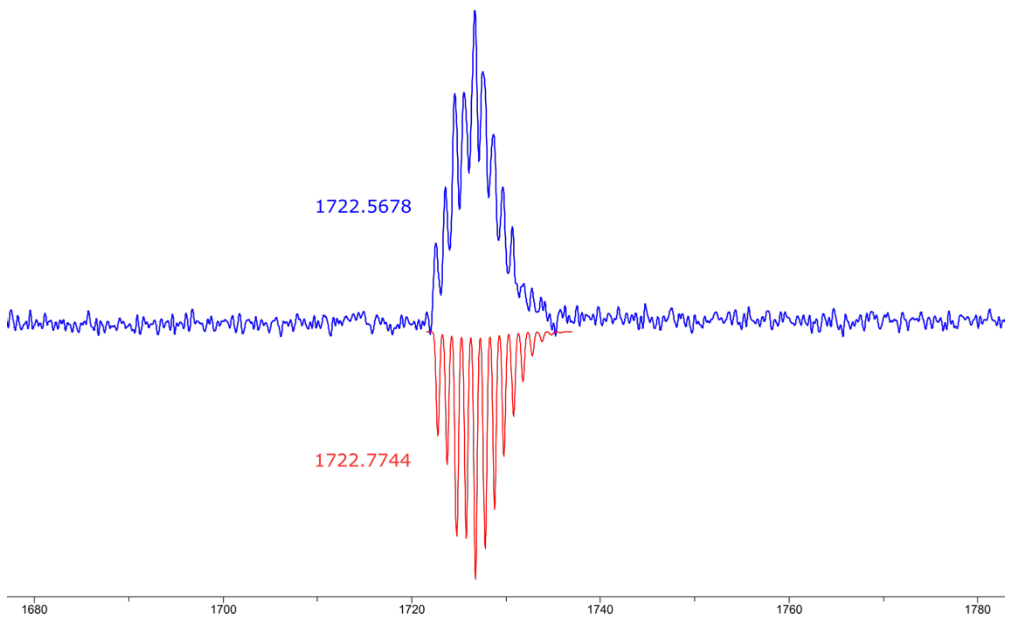

Figure S29. Selected region of a MALDI mass spectrum of **4** together with simulated isotopic pattern (in red), $[M]^{+}$.

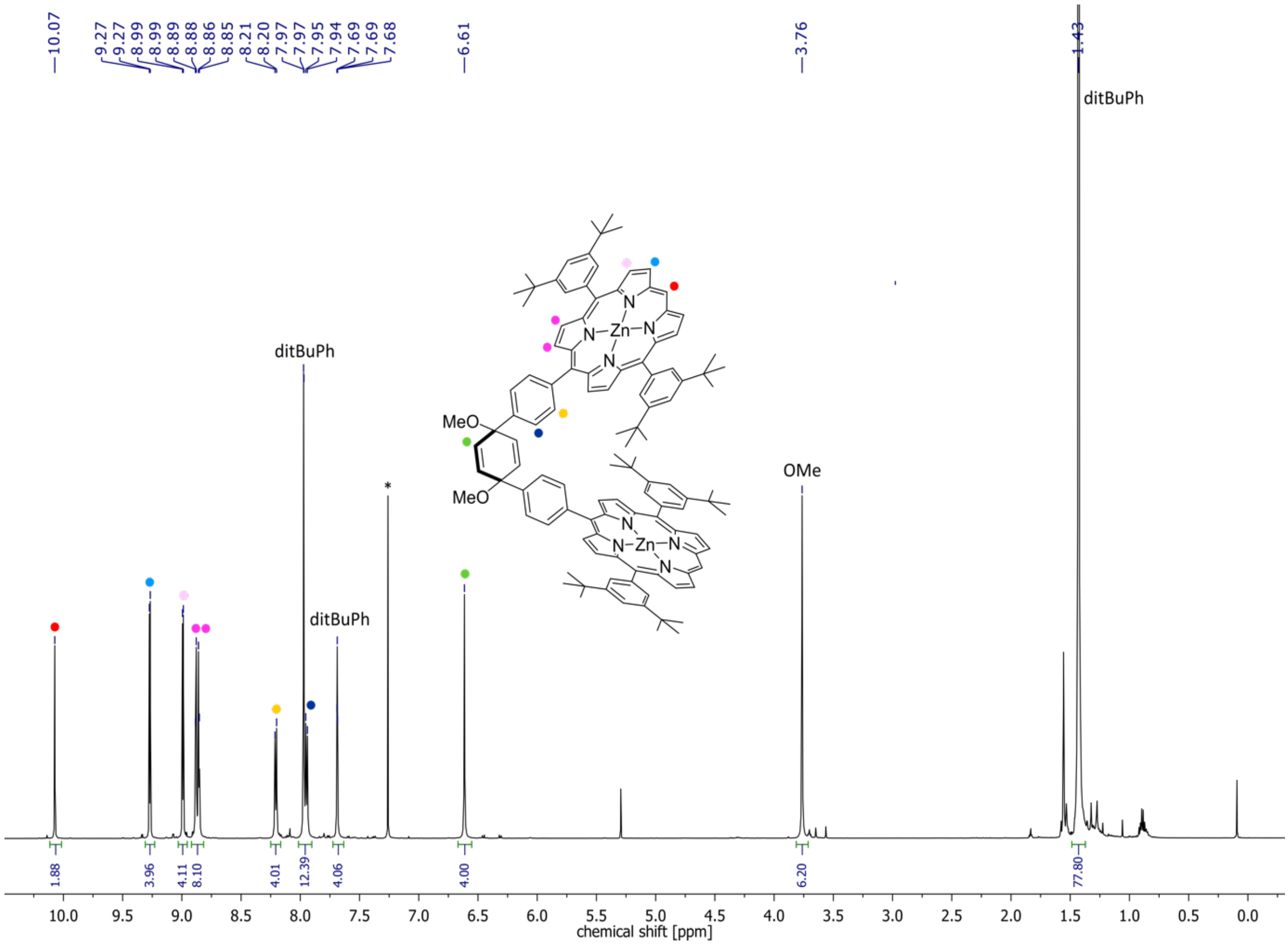

Figure S30. ^{1}H NMR spectrum of **3b** in $CDCl_3$ (600 MHz, 300 K).

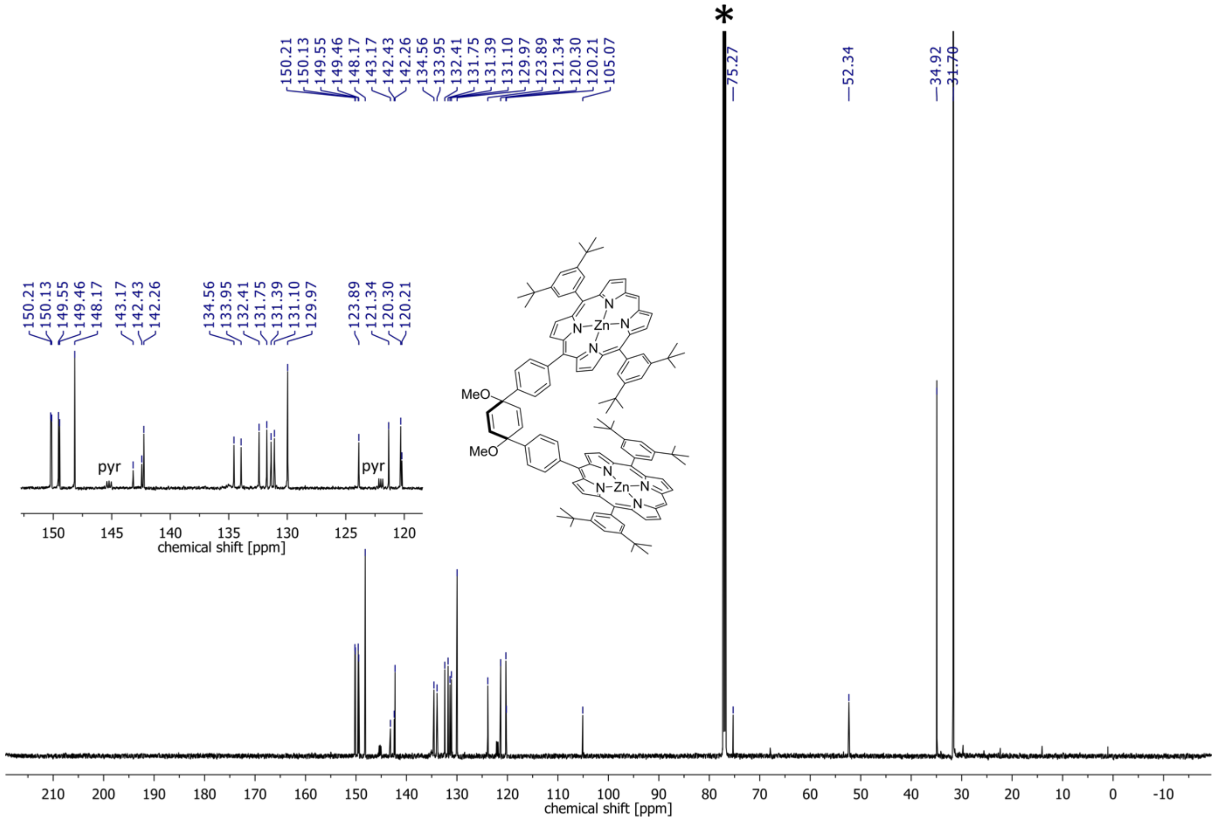

Figure S31. ^{13}C NMR spectrum of **3b** in $CDCl_3$ (151 MHz, 300 K).

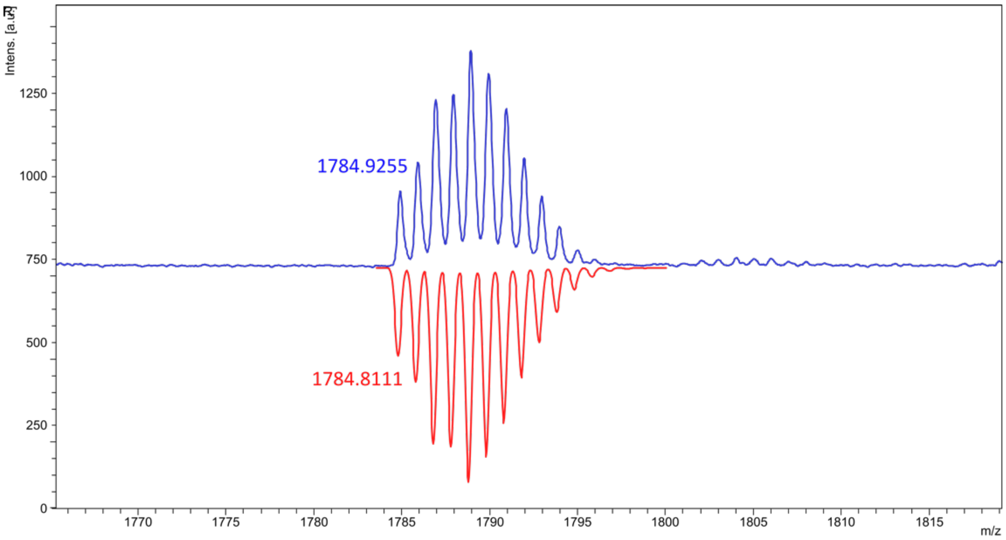

Figure S32. Selected region of a MALDI mass spectrum of **3b** together with simulated isotopic pattern (in red), $[M]^+$.

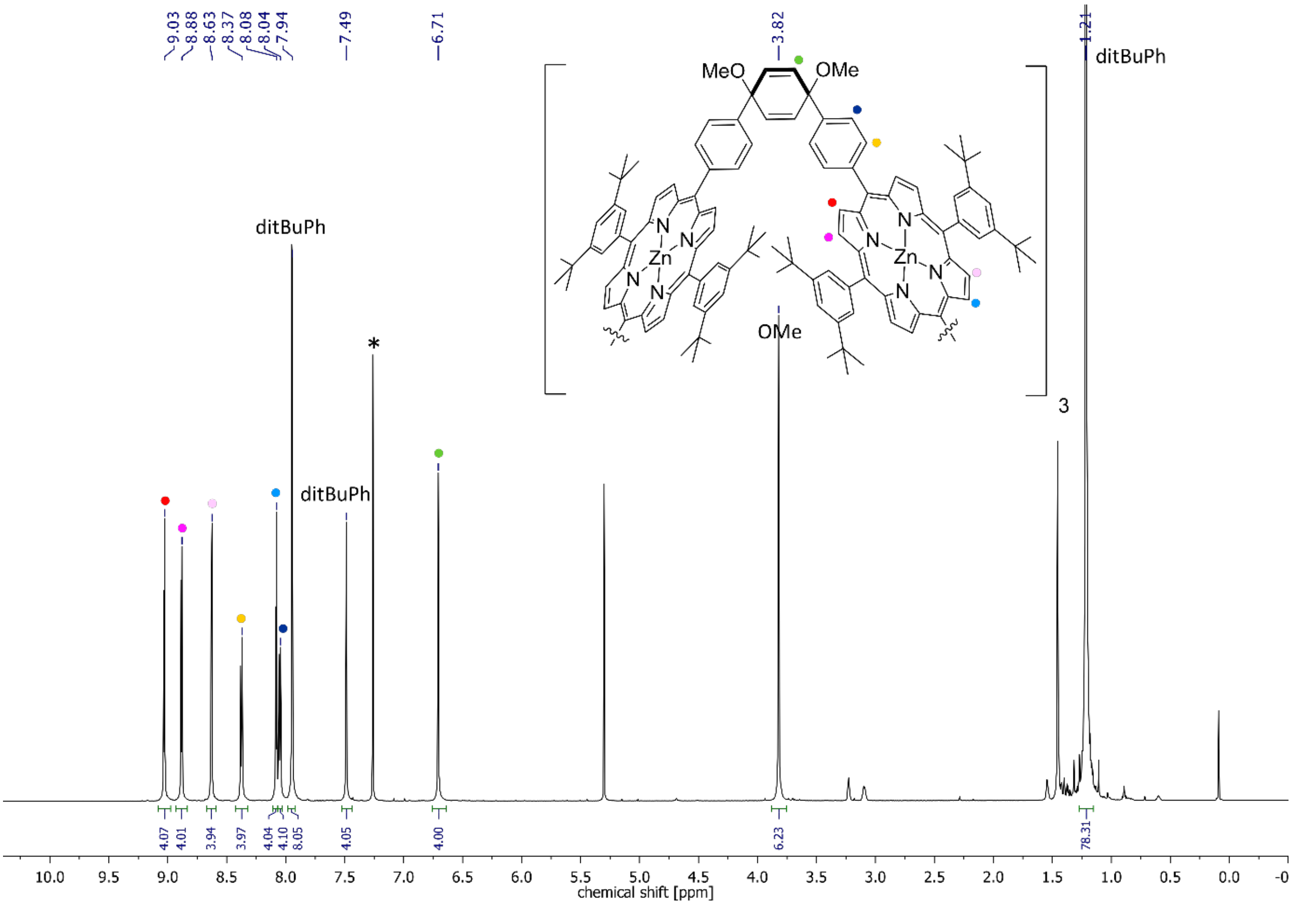

Figure S33. ^{1}H NMR spectrum of **5b** in $CDCl_3$ (600 MHz, 300 K).

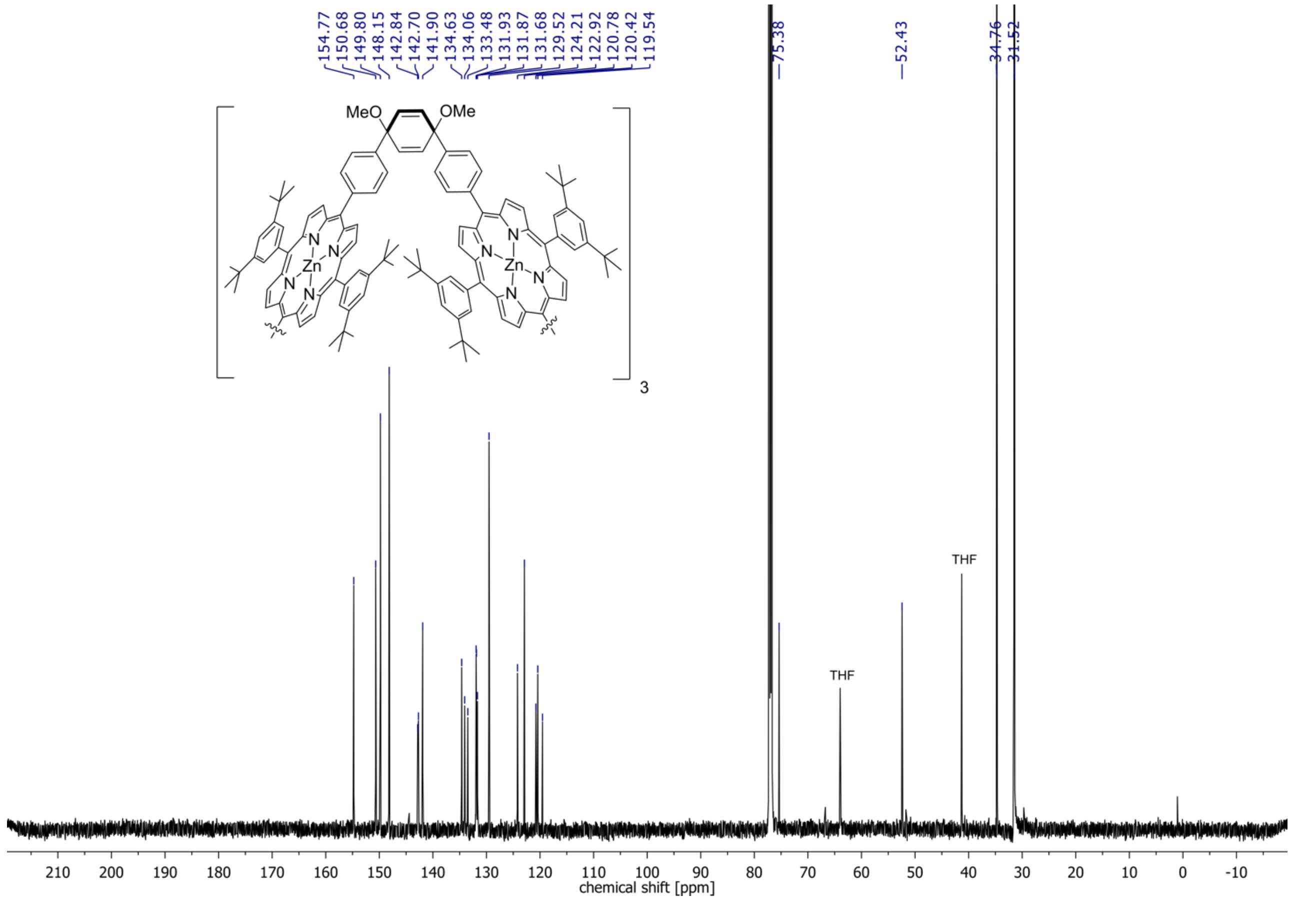

Figure S34. ^{13}C NMR spectrum of **5b** in $CDCl_3$ (151 MHz, 300 K).

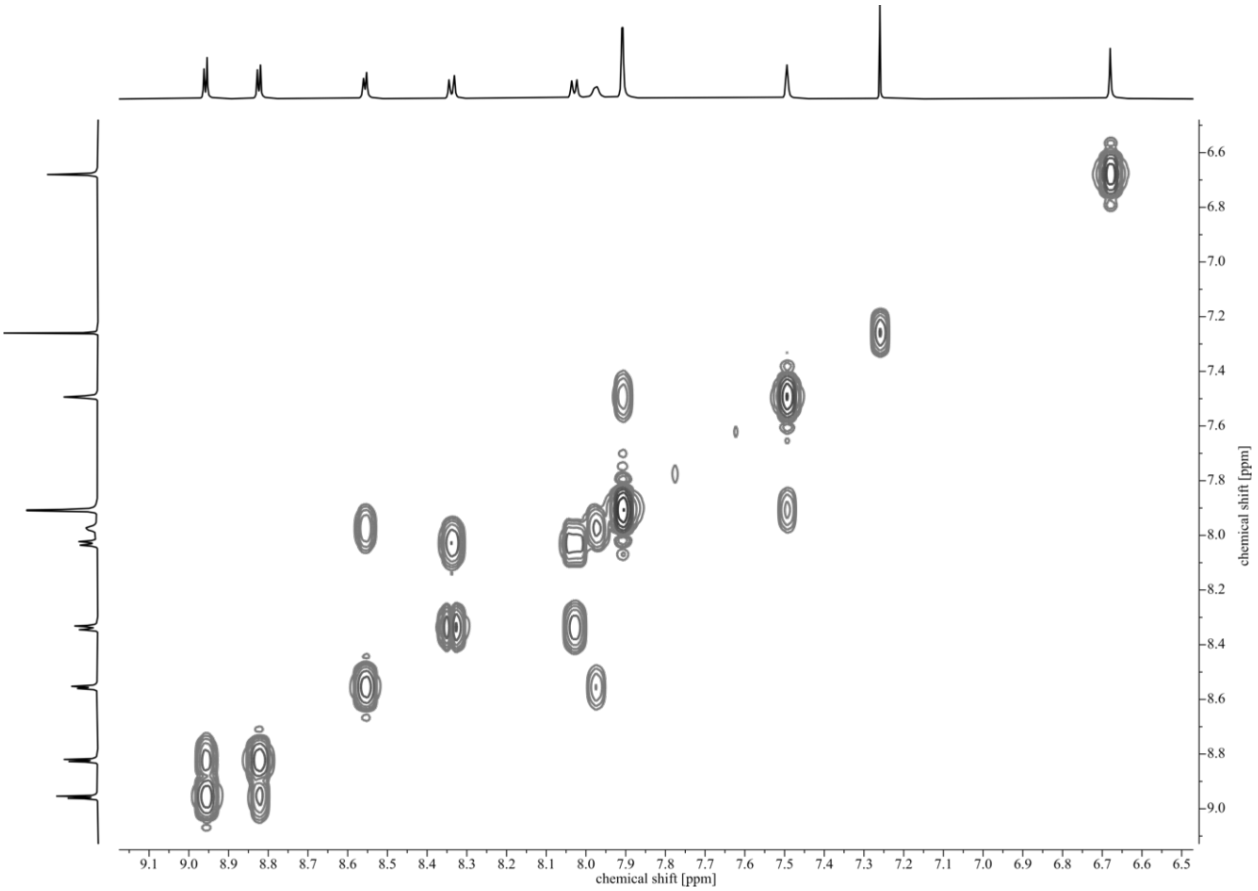

Figure S35. Selected region of a ^{1}H-^{1}H COSY NMR spectrum of **5b** in $CDCl_3$ (600 MHz, 300 K).

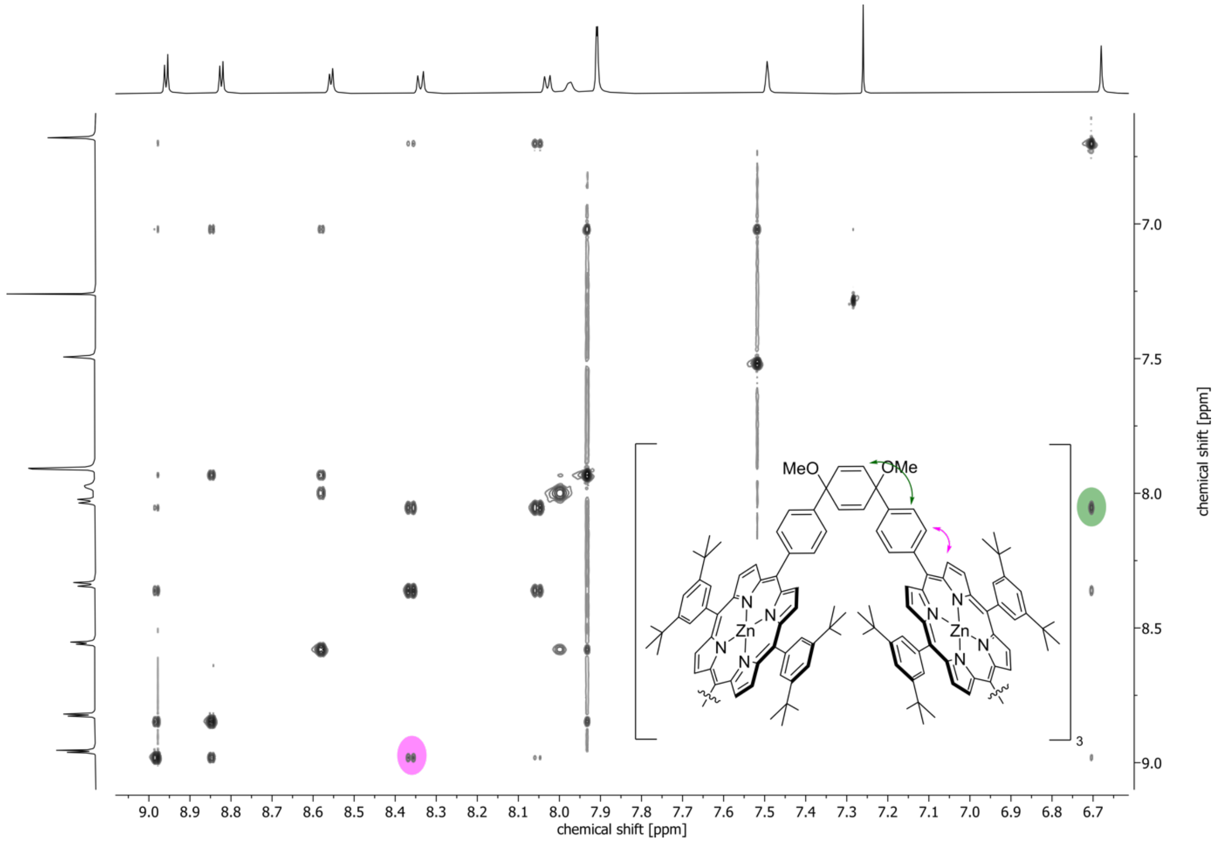

Figure S36. Selected region of a ^{1}H-^{1}H NOESY NMR spectrum of **5b** in $CDCl_3$ (600 MHz, 300 K) showing correlations crucial for the signals assignment.

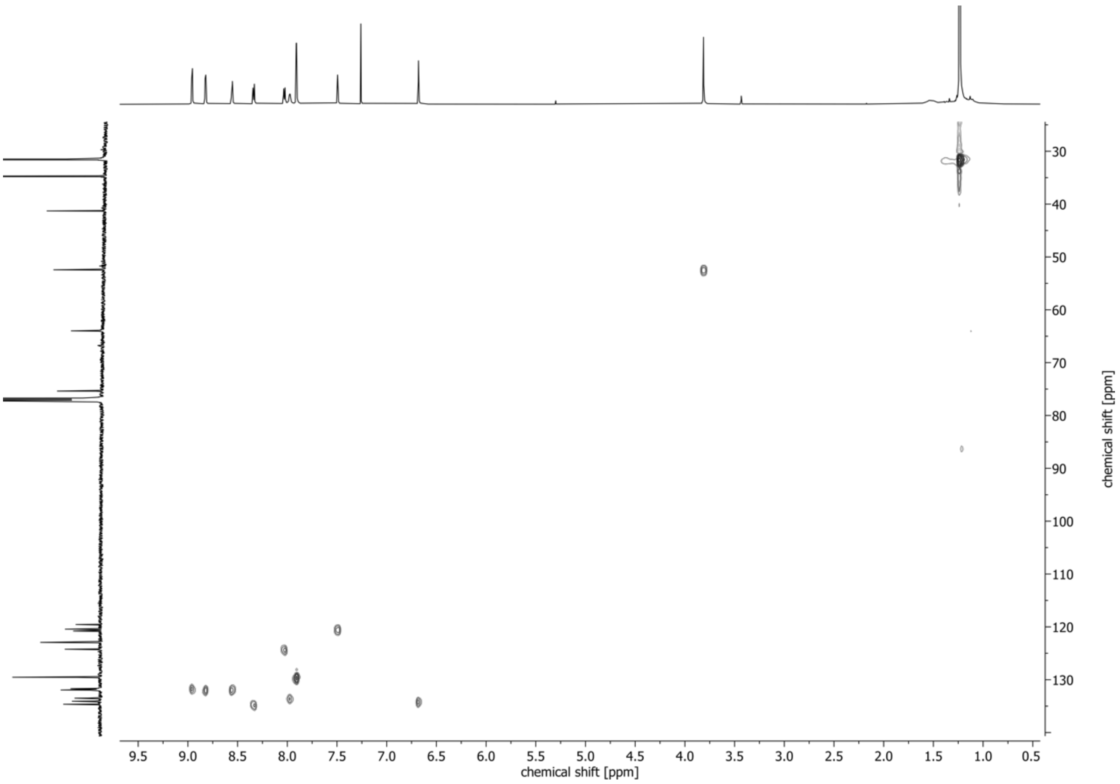

Figure S37. ^{1}H-^{13}C HSQC NMR spectrum of **5b** in $CDCl_3$ (600 MHz, 300 K).

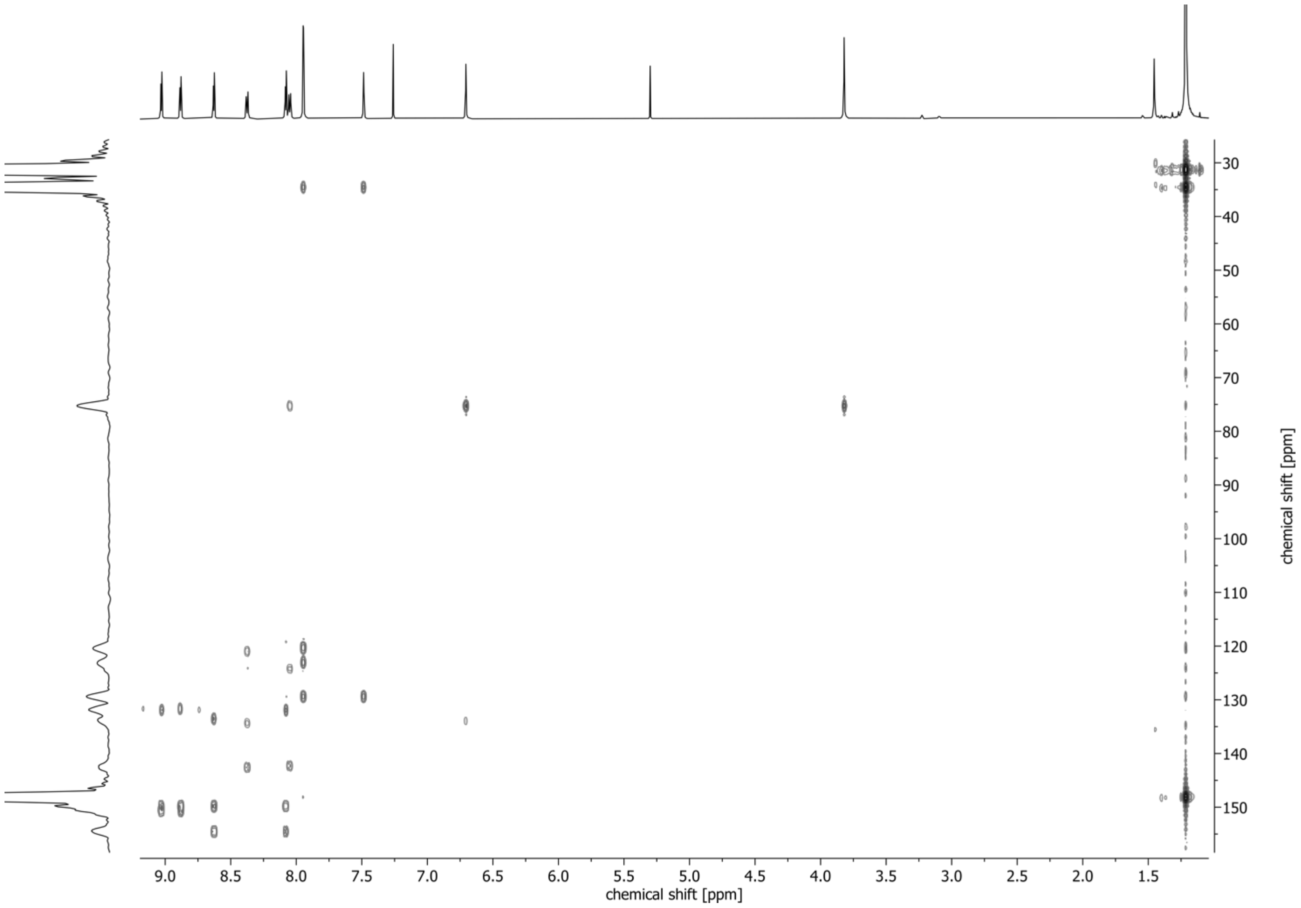

Figure S38. ^{1}H-^{13}C HMBC NMR spectrum of **5b** in $CDCl_3$ (600 MHz, 300 K).

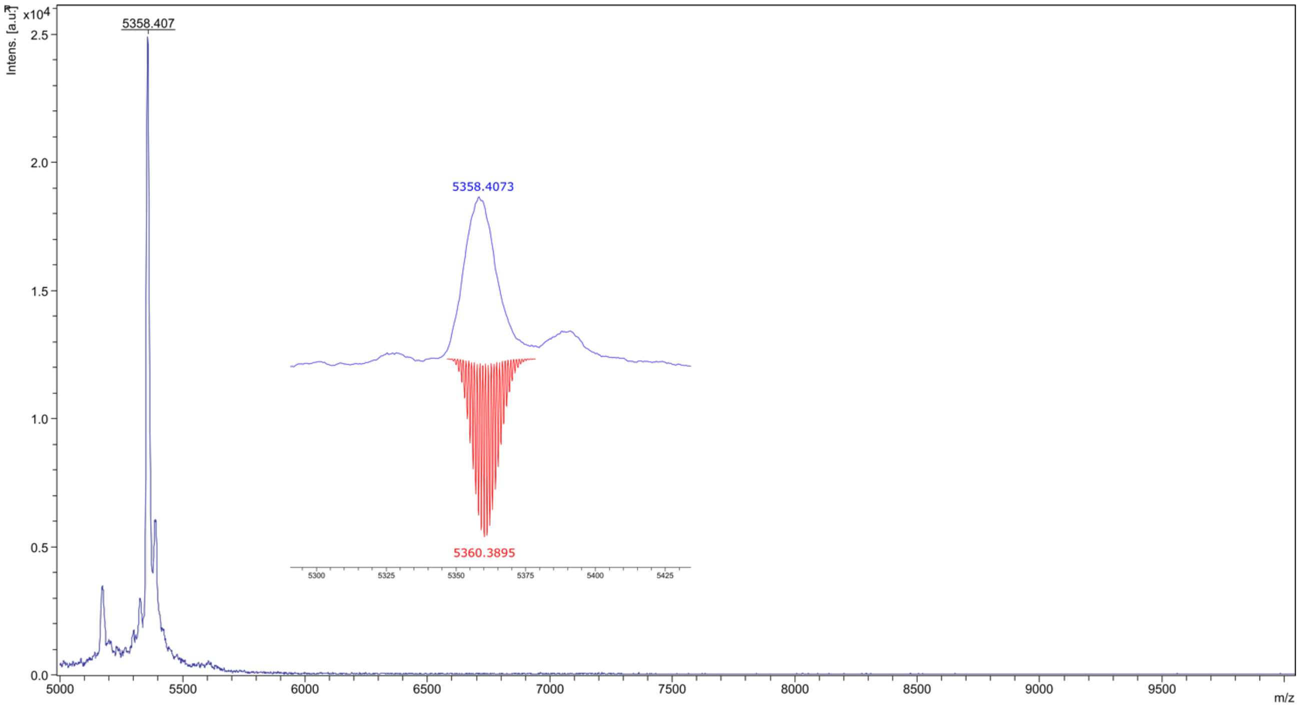

Figure S39. MALDI mass spectrum of **5b** together with simulated isotopic pattern (in red), $[M]^{+}$.

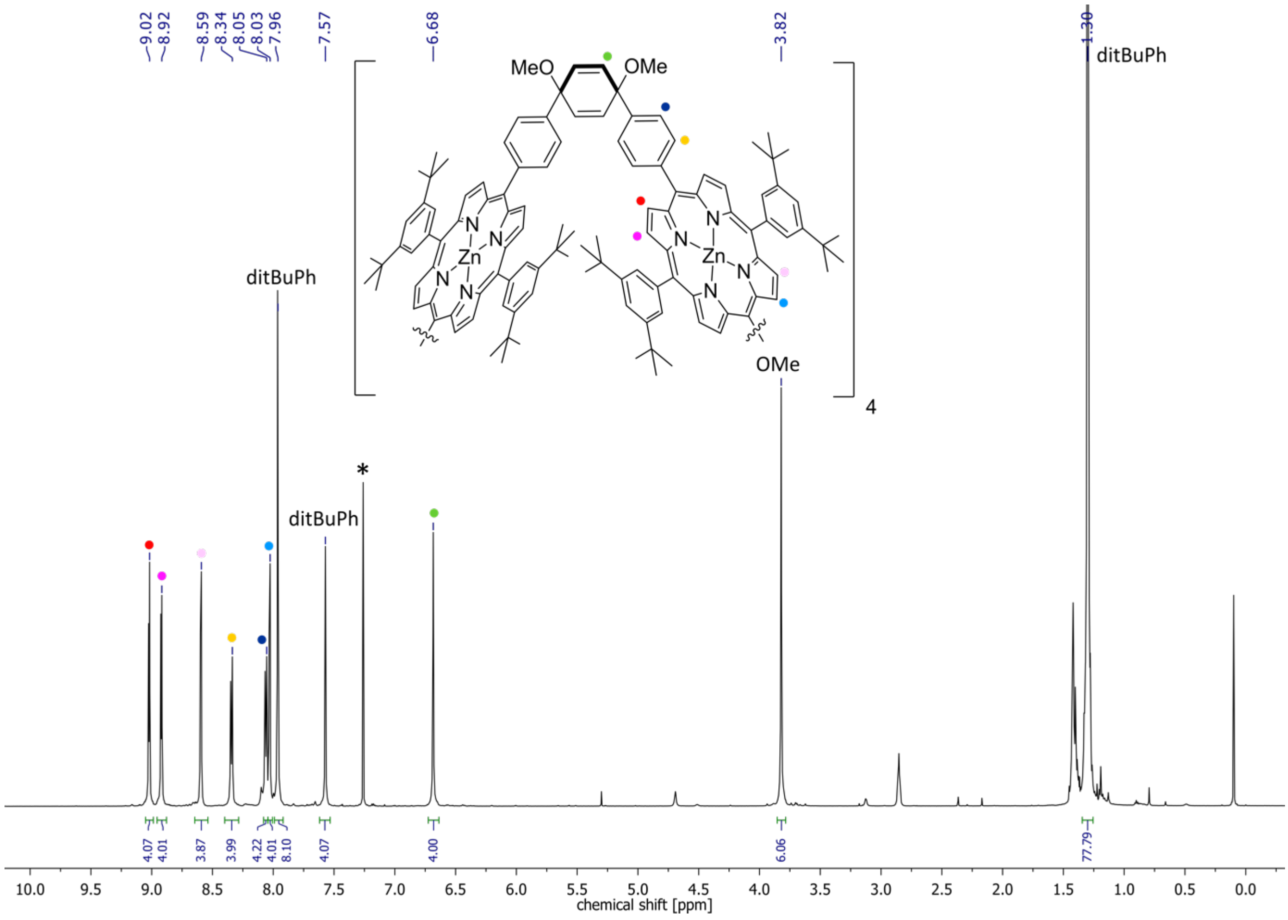

Figure S40. ^{1}H NMR spectrum of **6b** in $CDCl_3$ (600 MHz, 300 K).

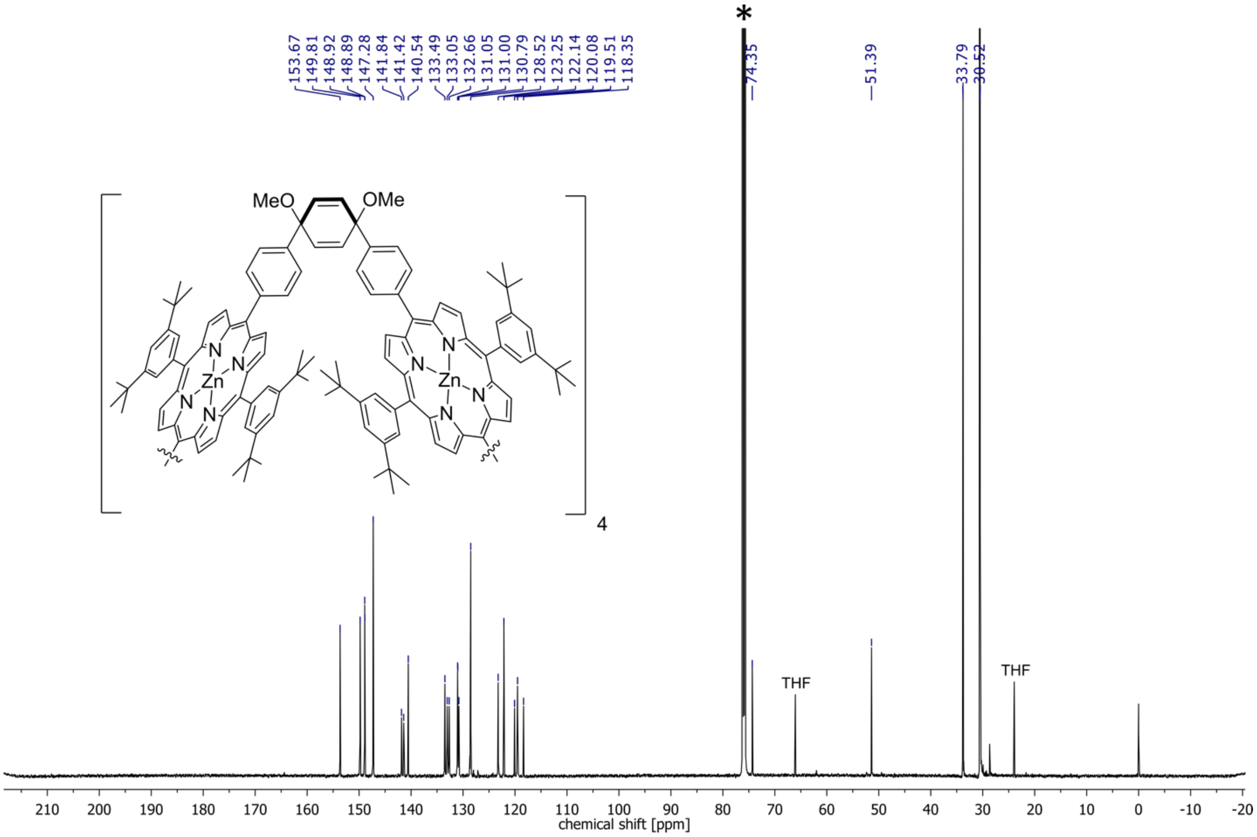

Figure S41. ^{13}C NMR spectrum of **6b** in $CDCl_3$ (151 MHz, 300 K).

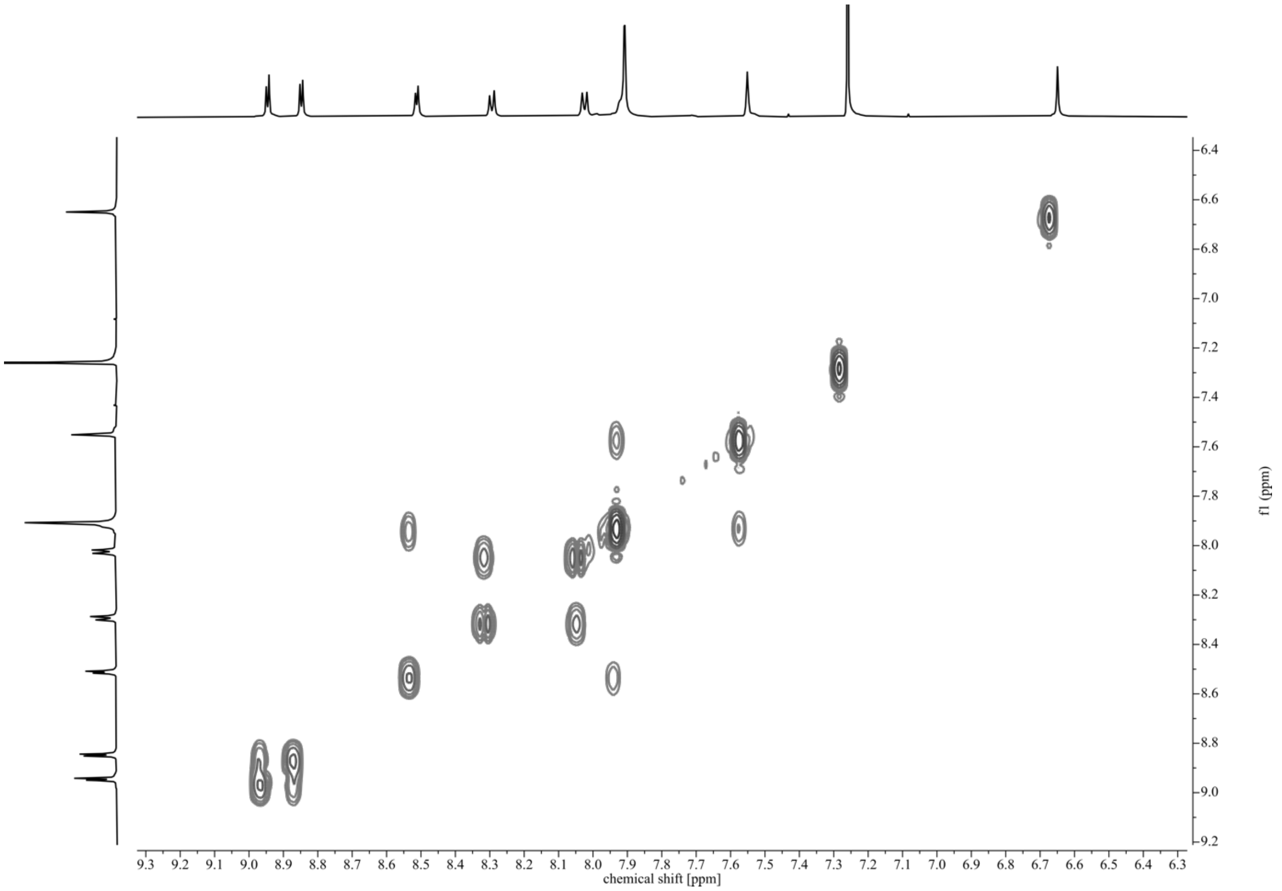

Figure S42. Selected region of a ^{1}H-^{1}H COSY NMR spectrum of **6b** in $CDCl_3$ (600 MHz, 300 K).

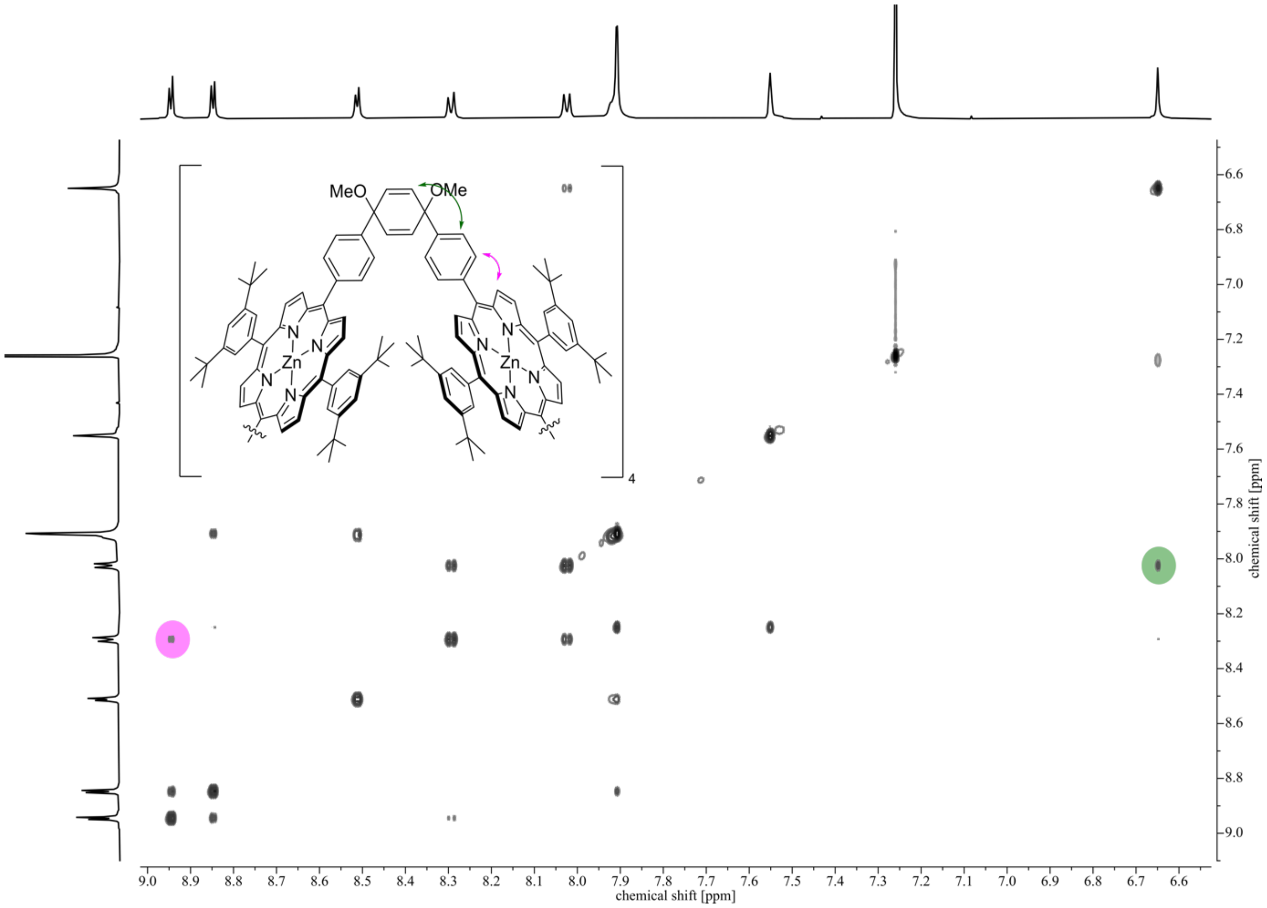

Figure S43. Selected region of a ^{1}H-^{1}H NOESY NMR spectrum of **6b** in $CDCl_3$ (600 MHz, 300 K) showing correlations crucial for the signals assignment.

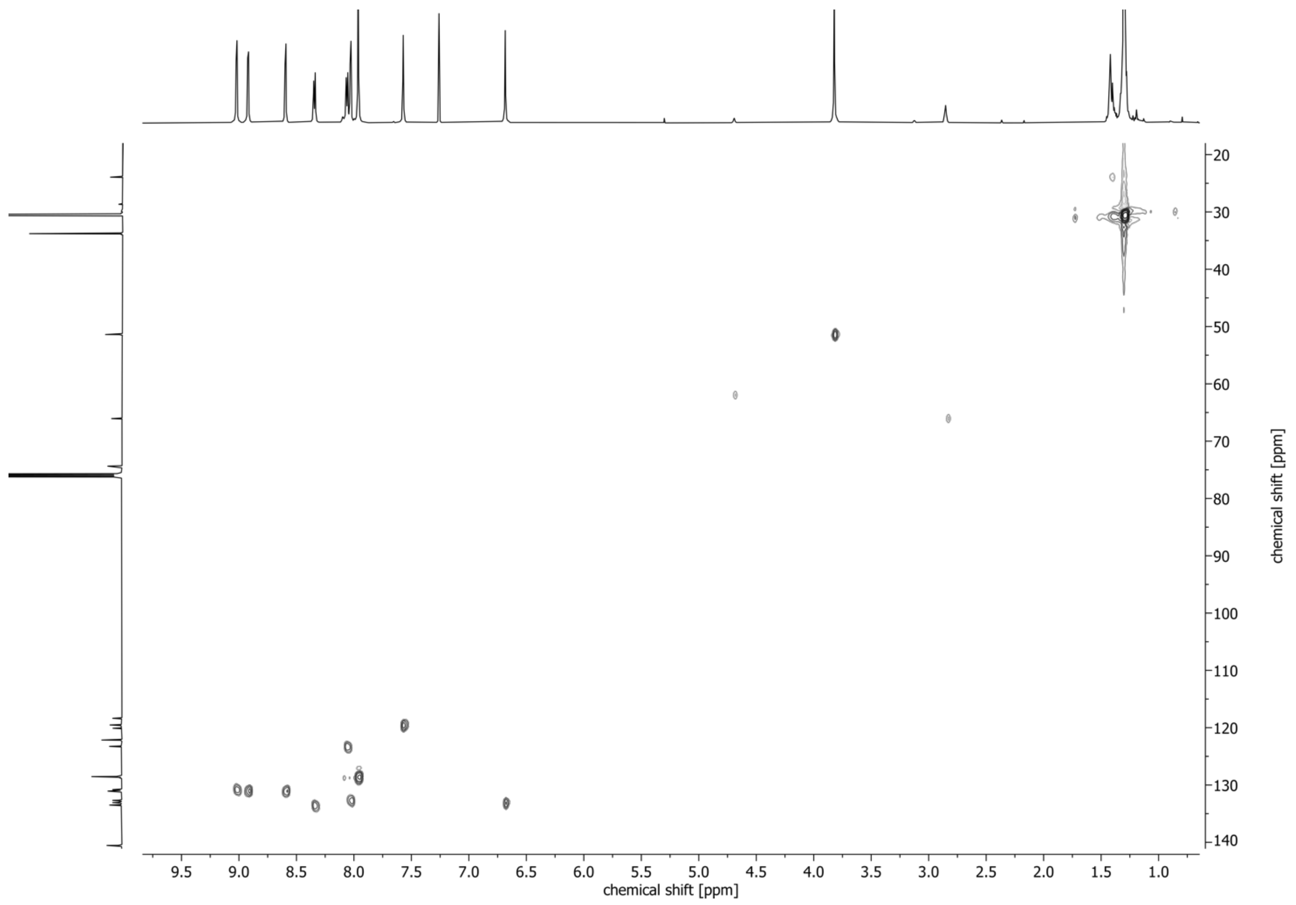

Figure S44. ^{1}H-^{13}C HSQC NMR spectrum of **6b** in $CDCl_3$ (600 MHz, 300 K).

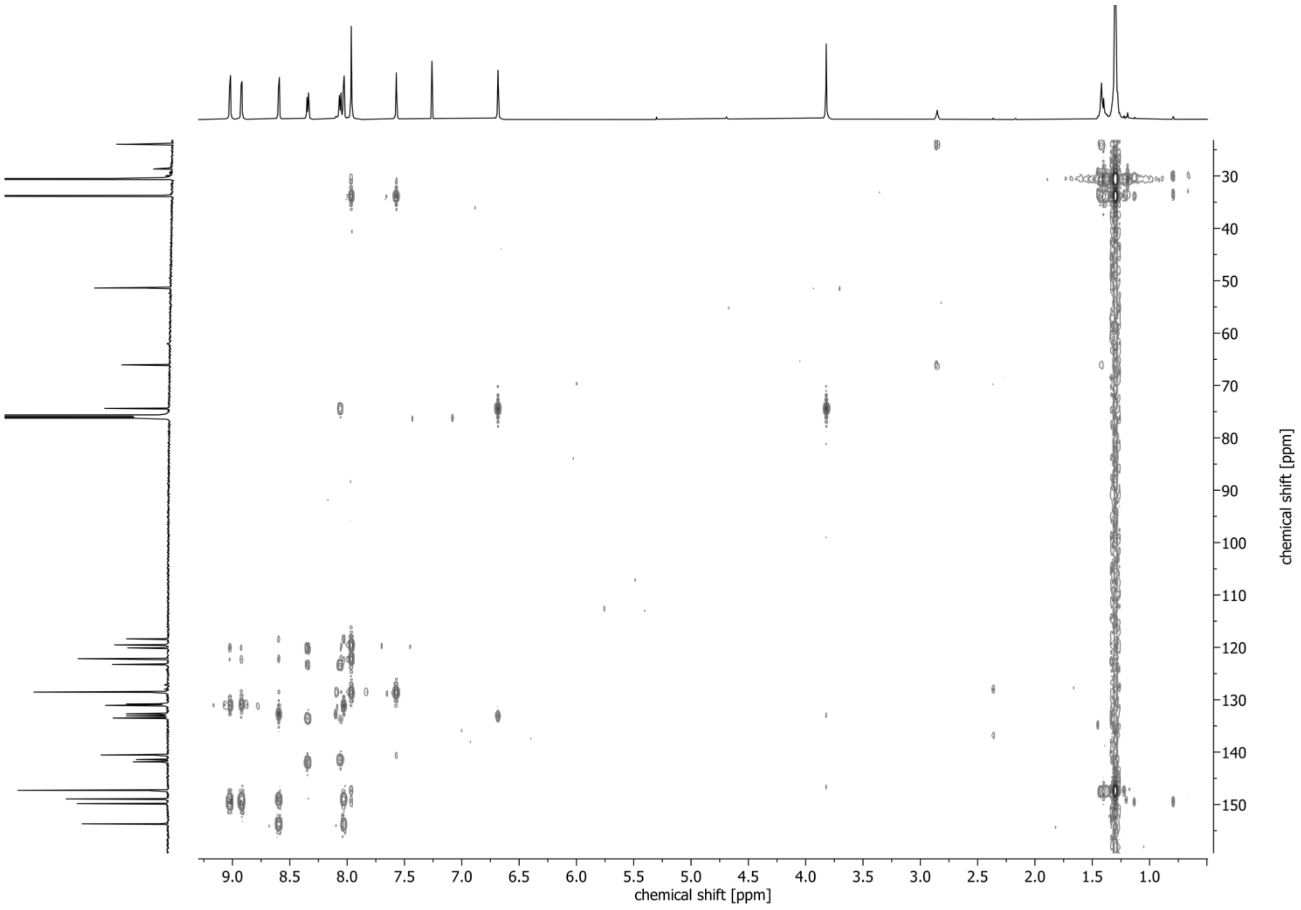

Figure S45. ^{1}H-^{13}C HMBC NMR spectrum of **6b** in $CDCl_3$ (600 MHz, 300 K).

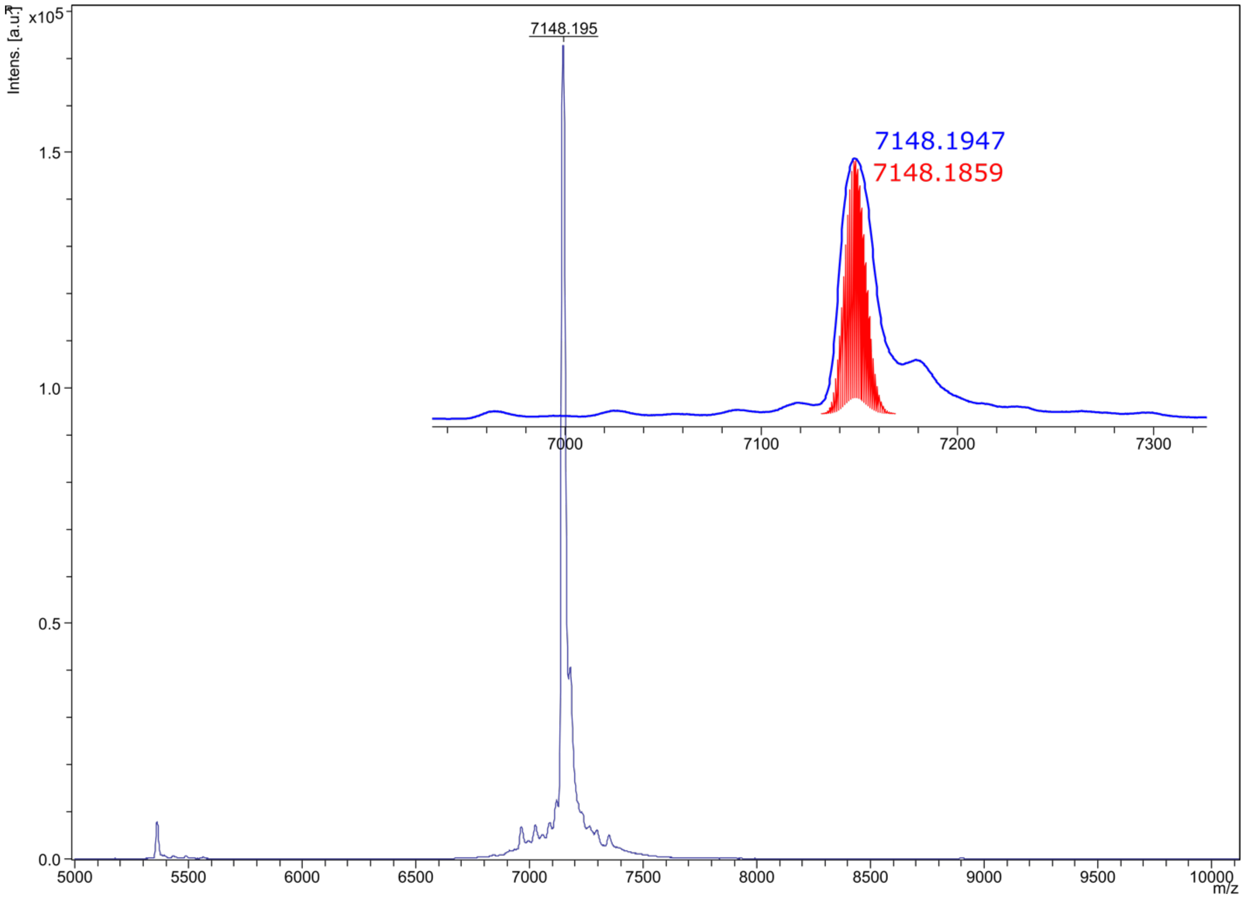

Figure S46. MALDI mass spectrum of **6b** together with simulated isotopic pattern (in red), $[M]^+$.

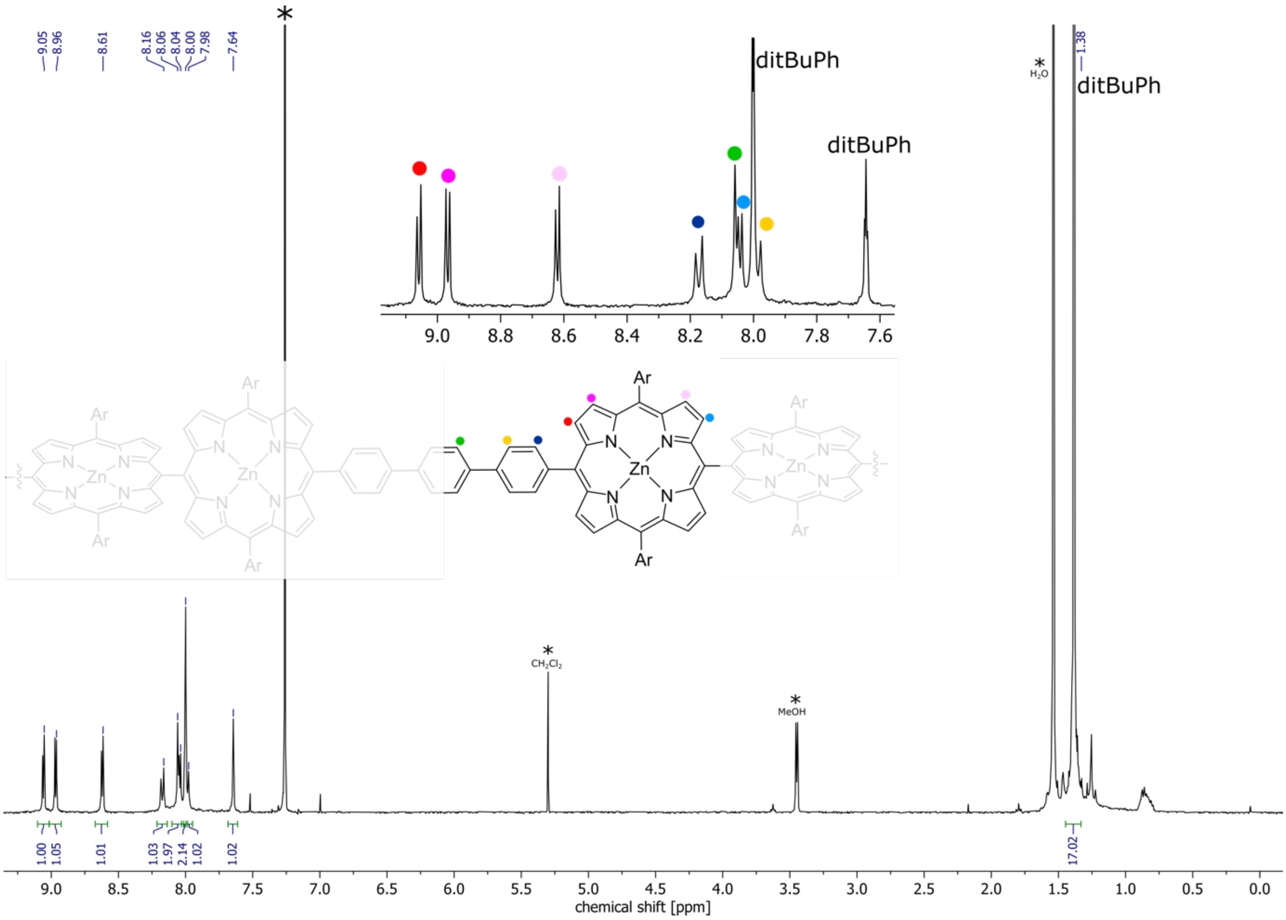

Figure S47. ^1H NMR spectrum of **7** in $CDCl_3$ (600 MHz, 300 K).

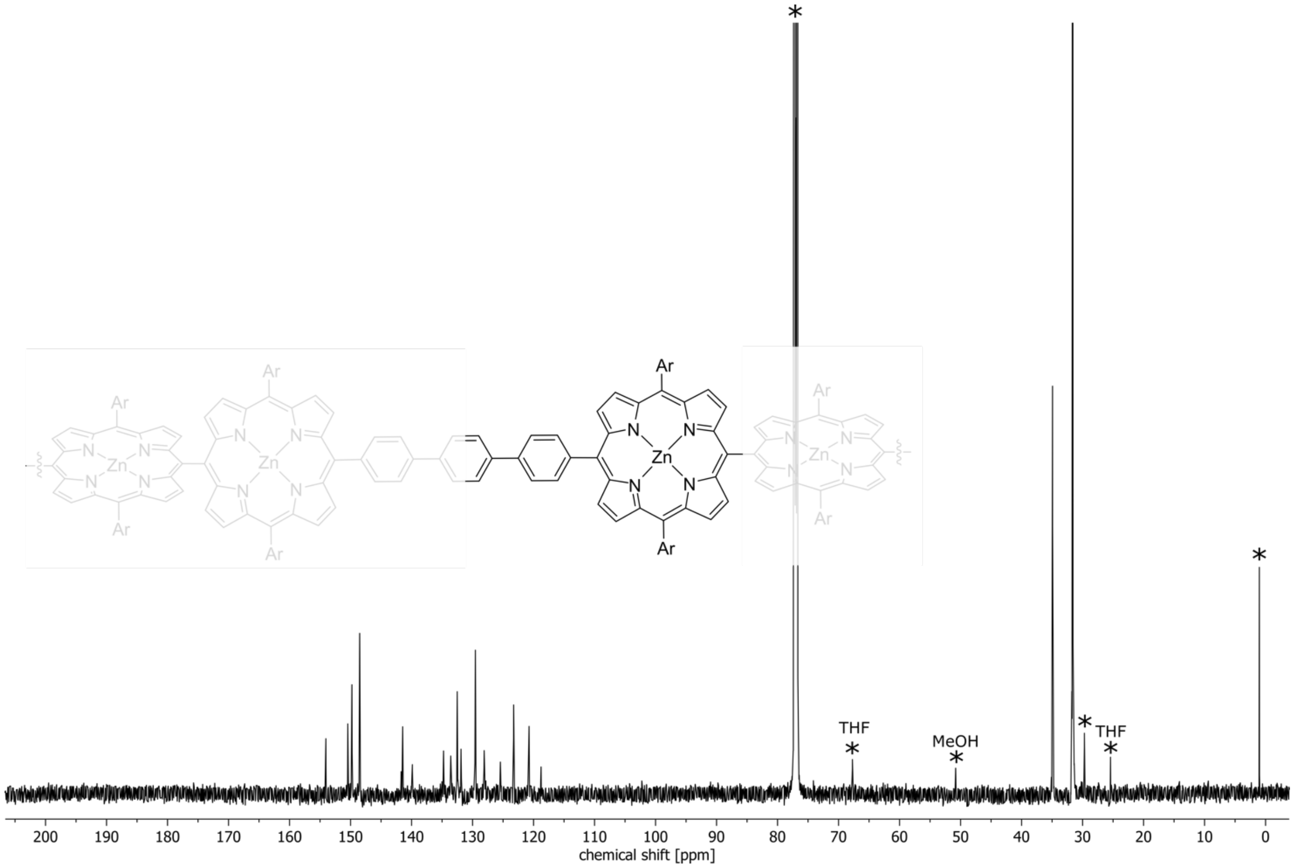

Figure S48. ^{13}C NMR spectrum of **7** in $CDCl_3$ (151 MHz, 300 K).

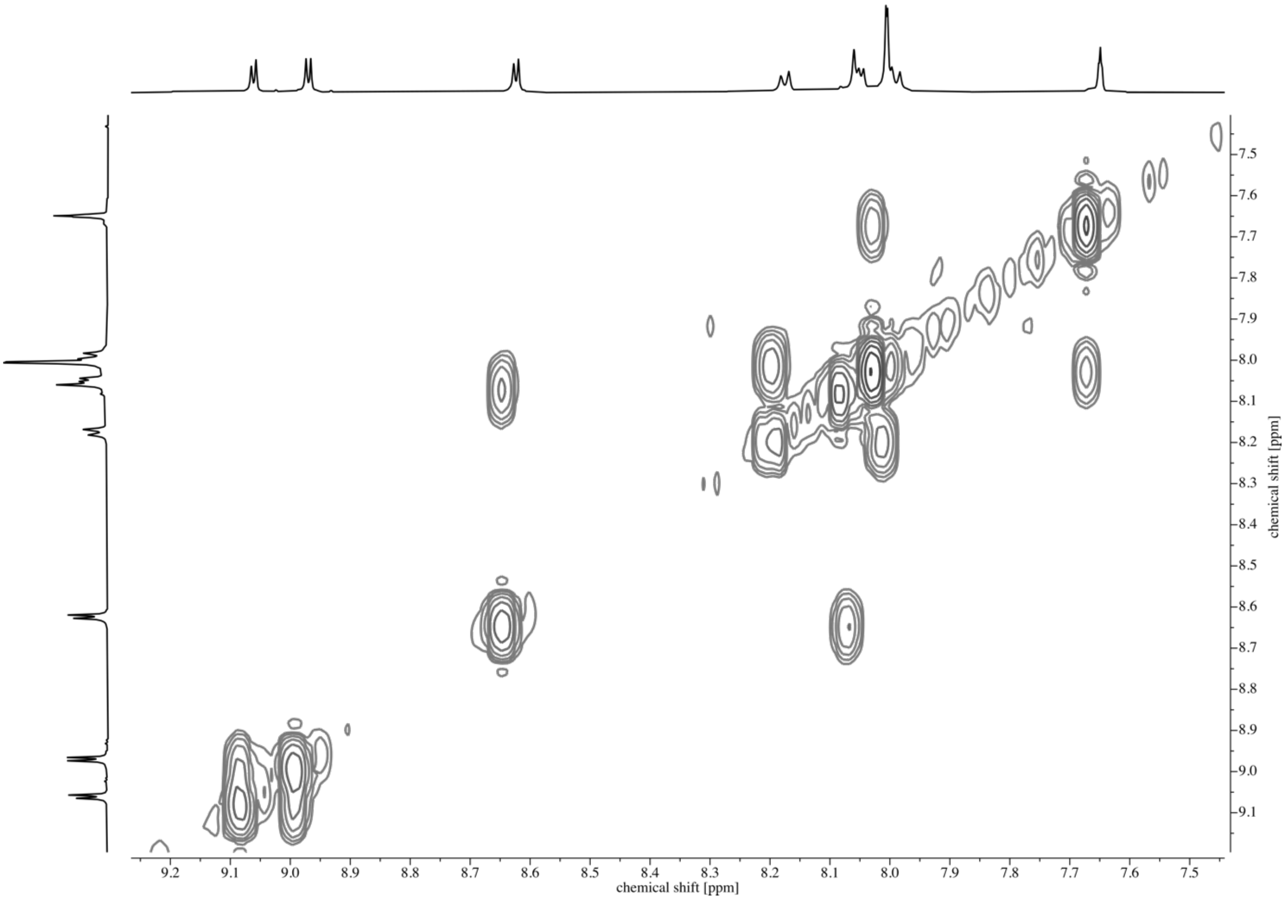

Figure S49. Selected region of a ^{1}H-^{1}H COSY NMR spectrum of **7** in $CDCl_3$ (600 MHz, 300 K).

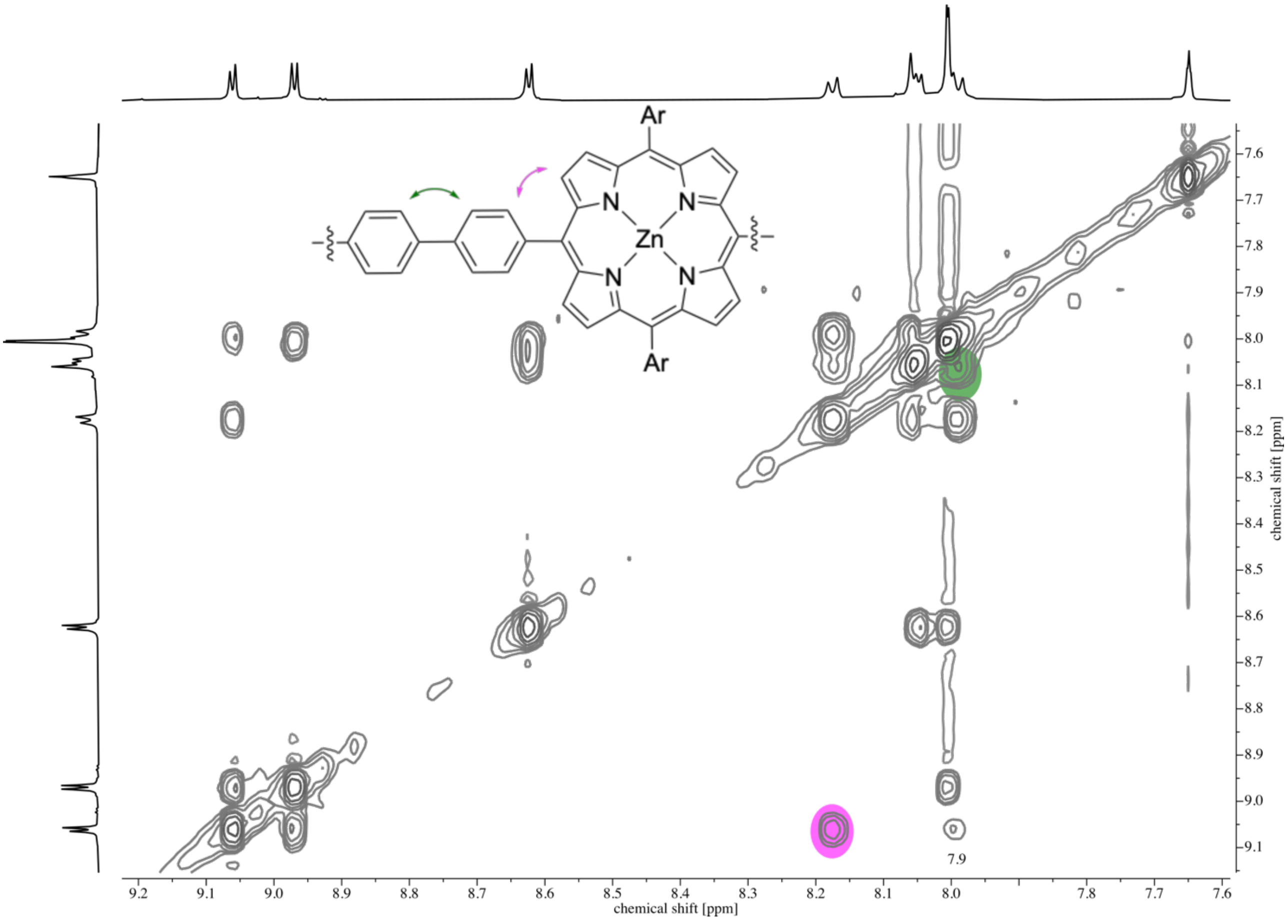

Figure S50. Selected region of a ^{1}H-^{1}H NOESY NMR spectrum of **7** in $CDCl_3$ (600 MHz, 300 K) with marked correlations crucial for signals assignment.

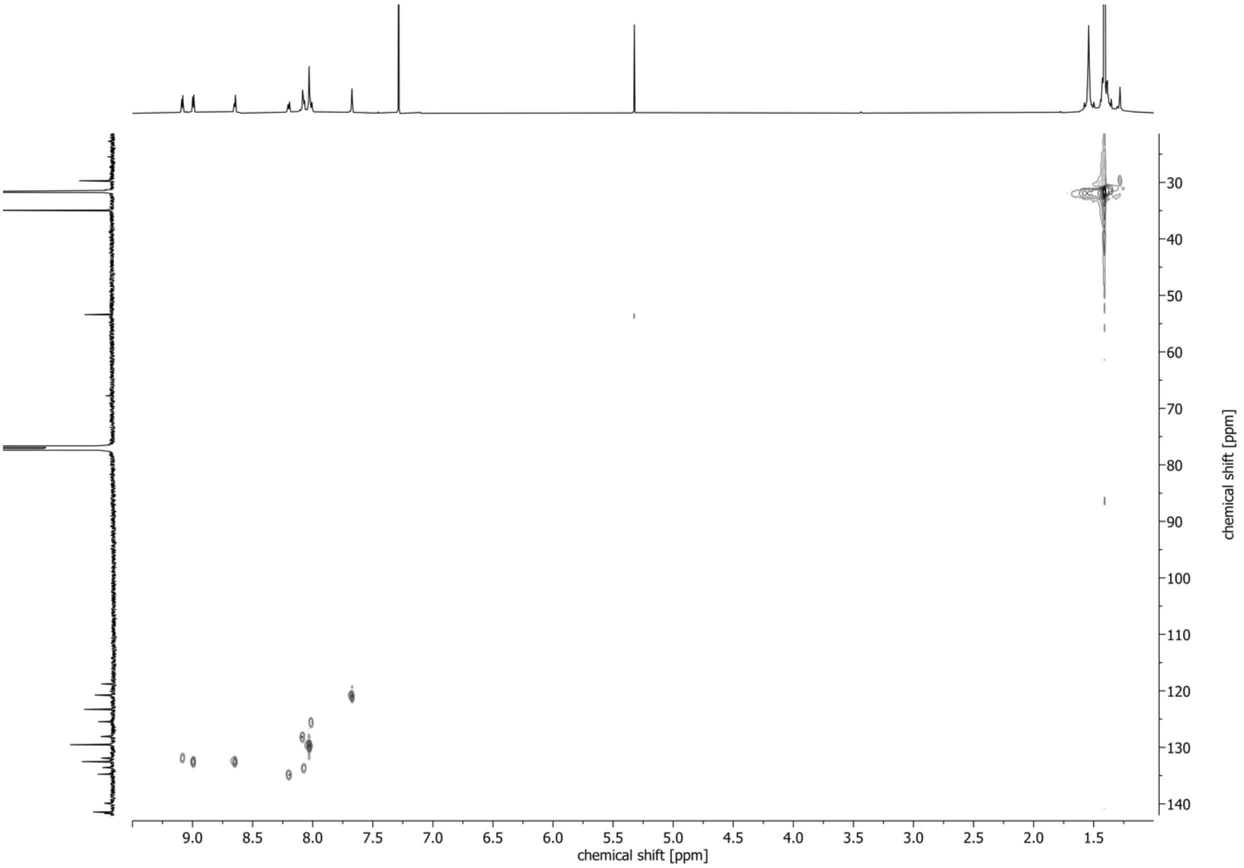

Figure S51. Selected region of a ^{1}H-^{13}C HSQC NMR spectrum of **7** in $CDCl_3$ (600 MHz, 300 K).

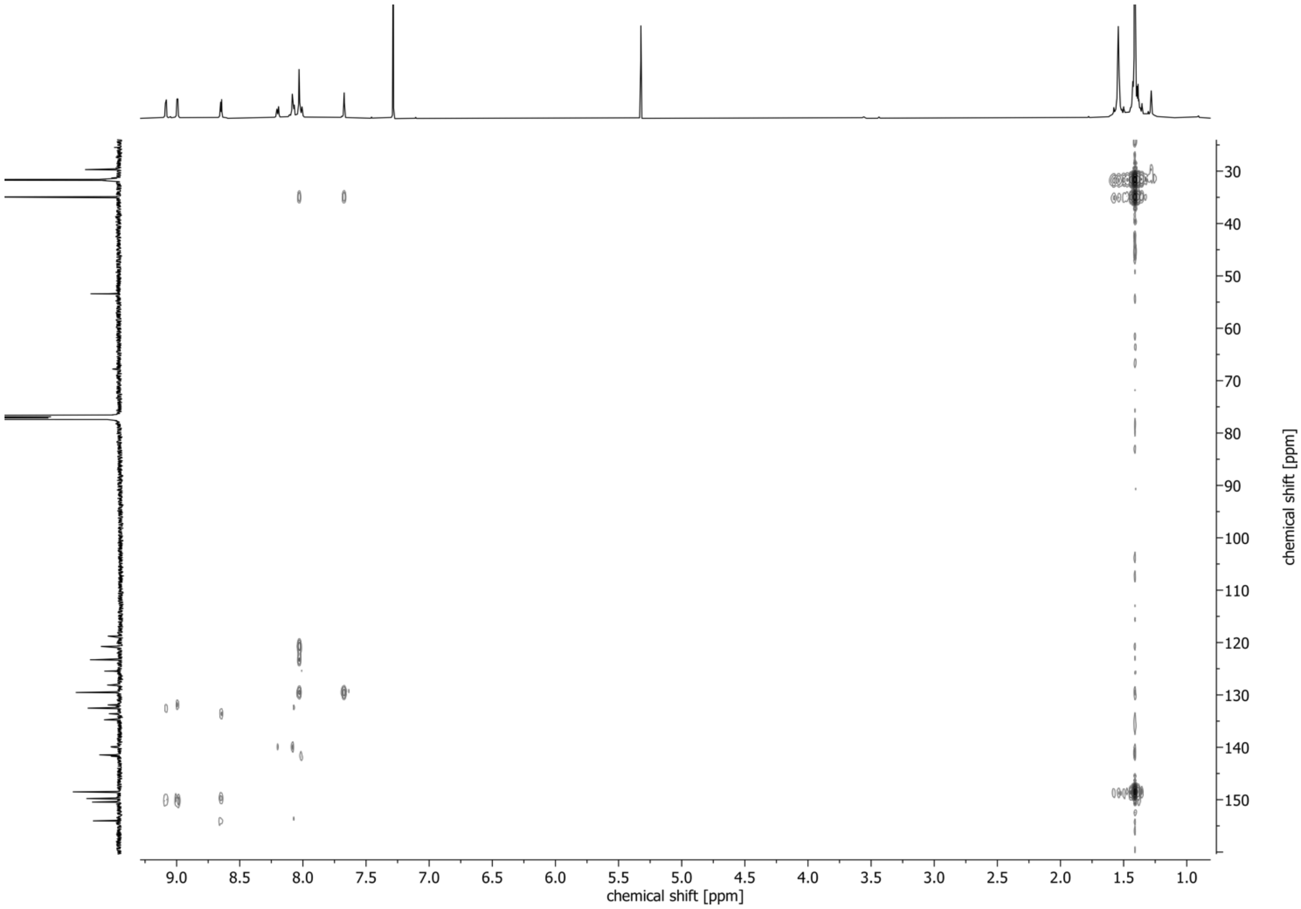

Figure S52. Selected region of a ^{1}H-^{13}C HMBC NMR spectrum of **7** in $CDCl_3$ (600 MHz, 300 K).

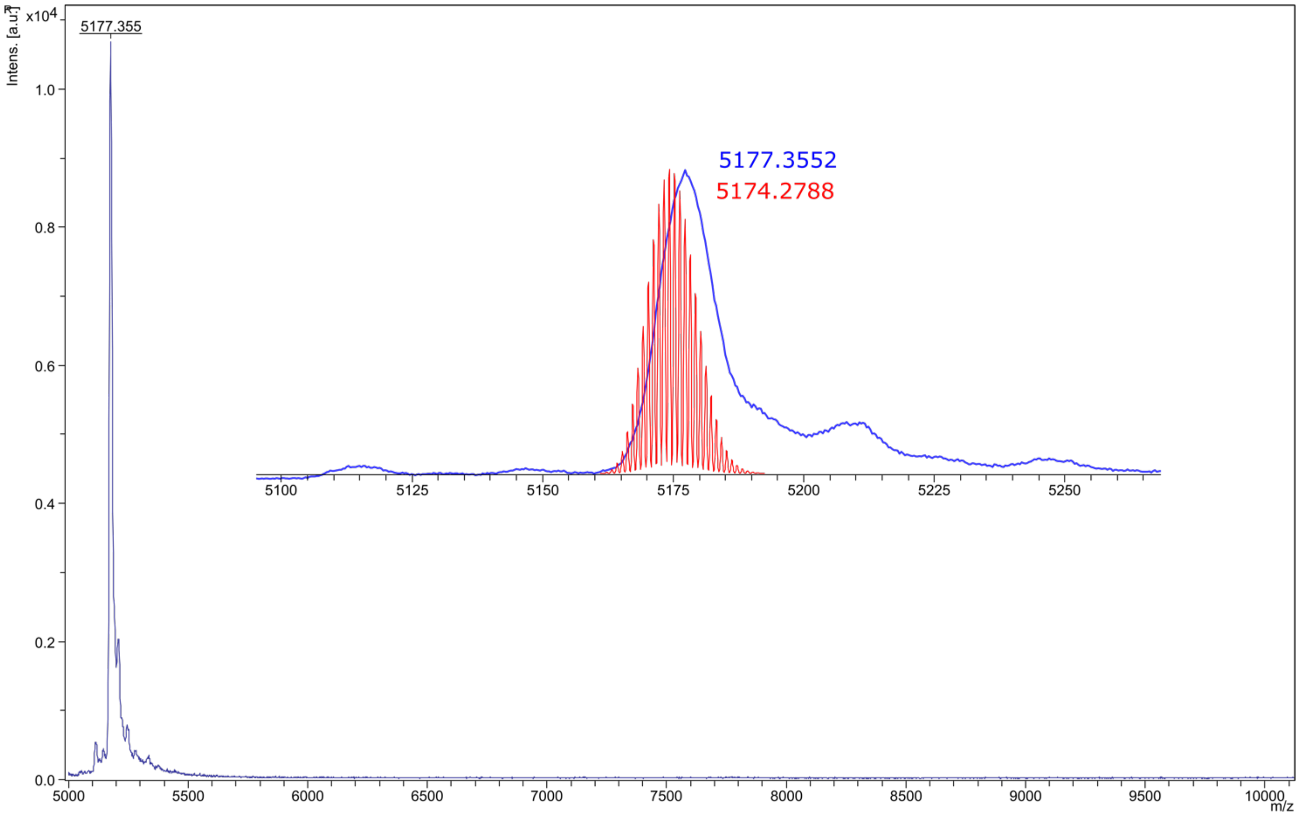

Figure S53. MALDI mass spectrum of **7** together with simulated isotopic pattern (in red), $[M]^{+}$.

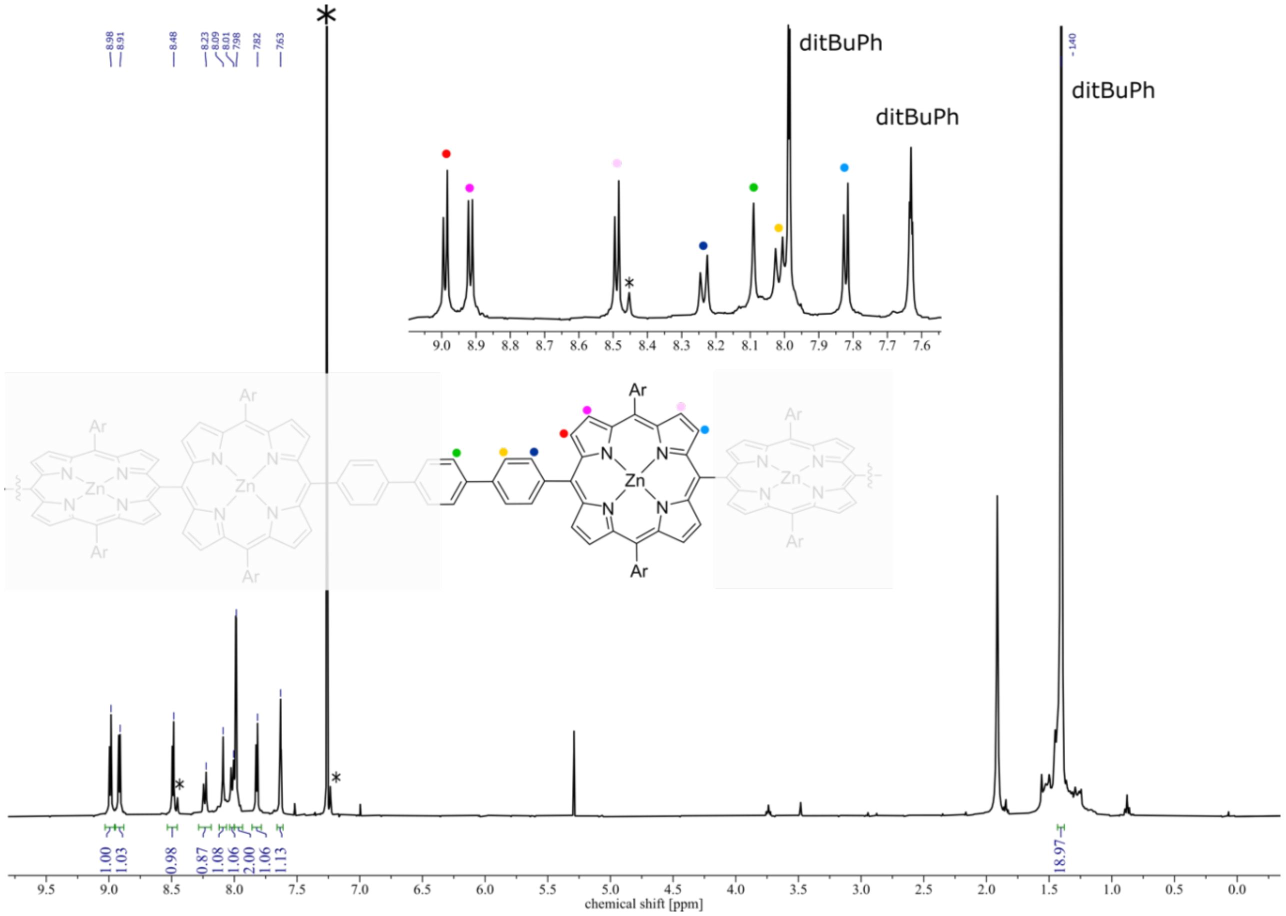

Figure S54. ^{1}H NMR spectrum of **8** in $CDCl_3$ + 1% pyr-d_5, (600 MHz, 300 K).

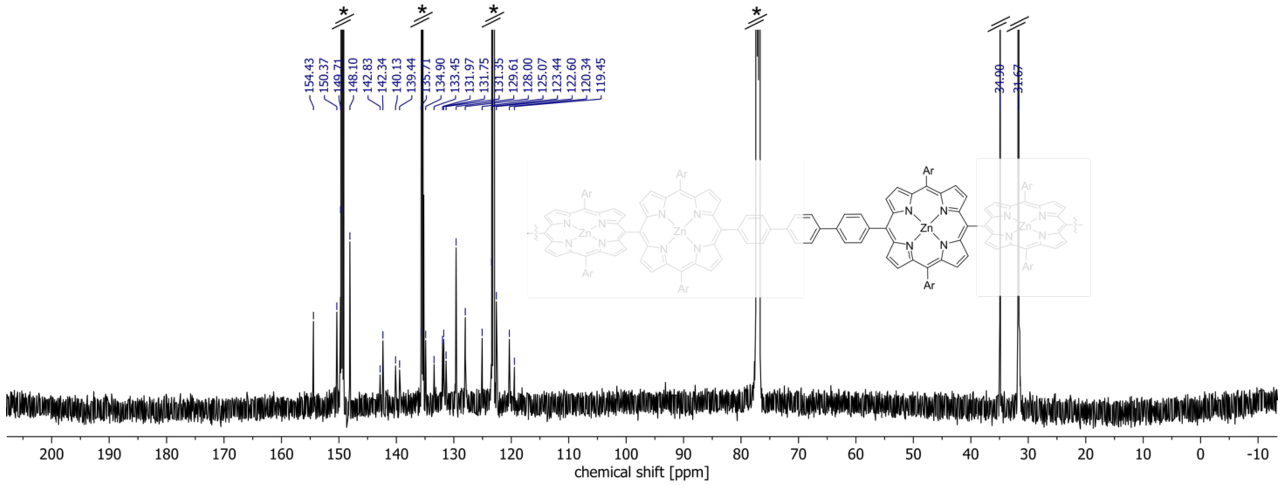

Figure S55. ^{13}C NMR spectrum of **8** in $CDCl_3$ + 1% pyr-d_5, (151 MHz, 300 K).

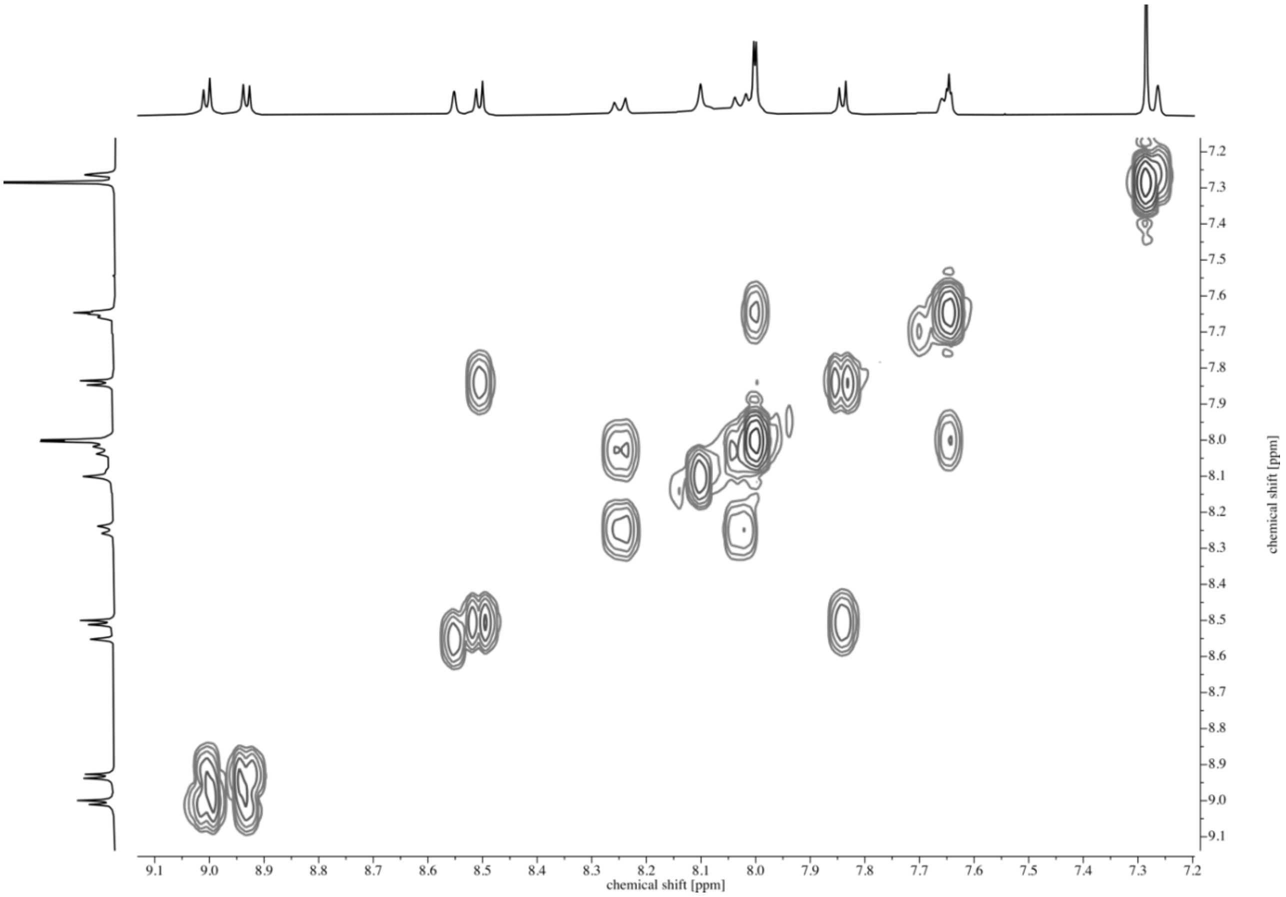

Figure S56. Selected region of a ^{1}H-^{1}H COSY NMR spectrum of **8** in $CDCl_3$ + 1% pyr-d_5 (600 MHz, 300 K).

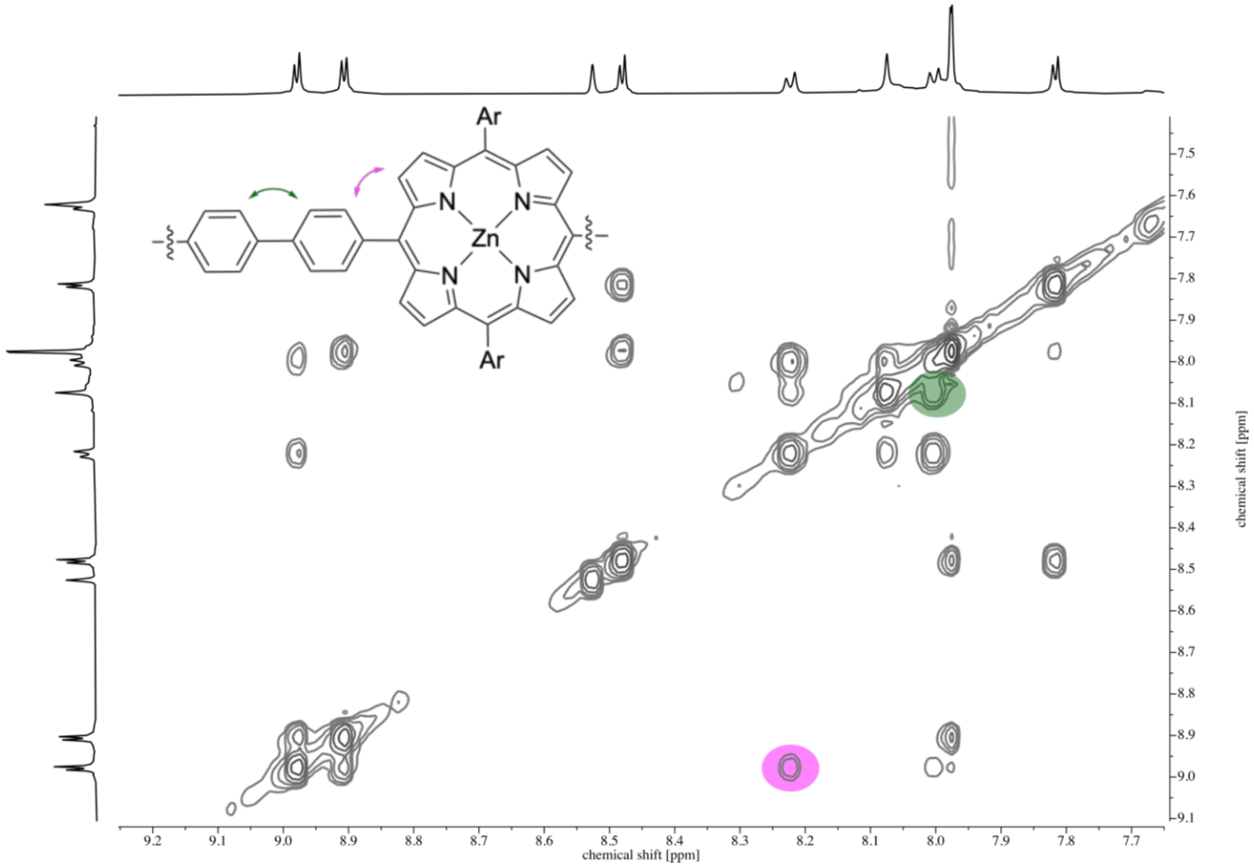

Figure S57. Selected region of a ^{1}H-^{1}H NOESY NMR spectrum of **8** in $CDCl_3$ + 1% pyr-d_5 (600 MHz, 300 K) with marked signals crucial for signal assignment.

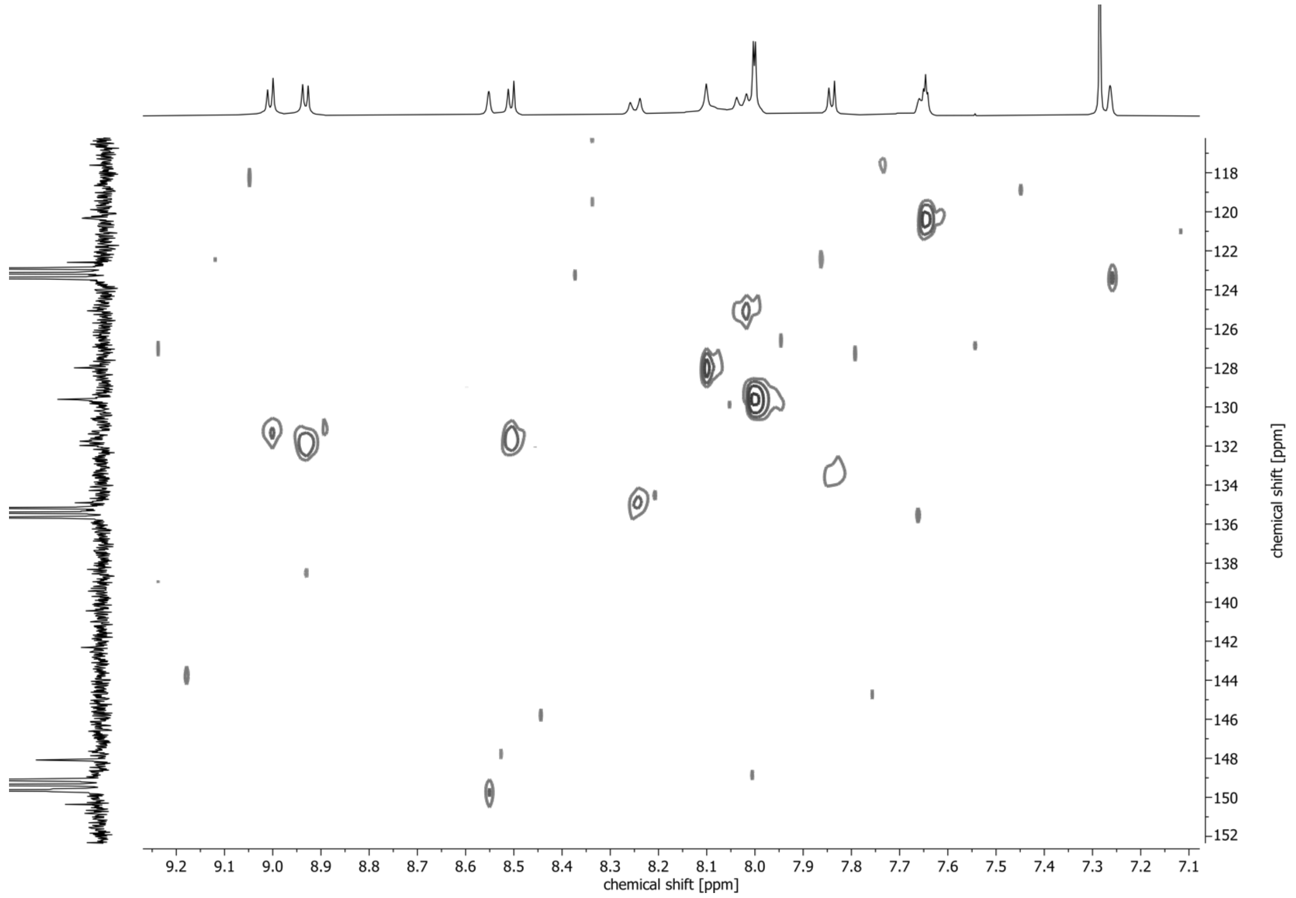

Figure S58. Selected region of a ^{1}H-^{13}C HSQC NMR spectrum of **8** in $CDCl_3$ + 1% pyr-*d*$_5$ (600 MHz, 300 K).

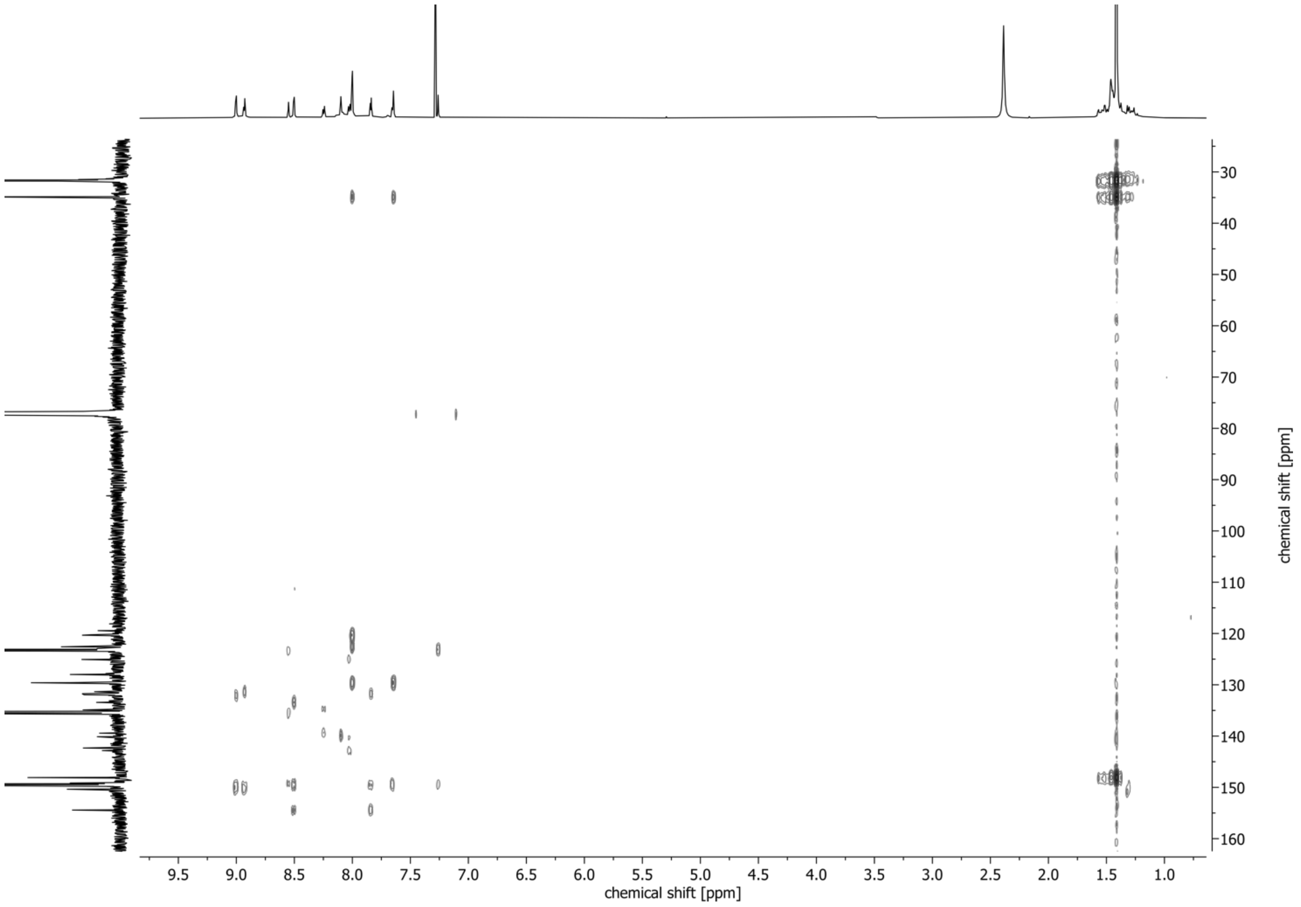

Figure S59. ^{1}H-^{13}C HMBC NMR spectrum of **8** in $CDCl_3$ + 1% pyr-*d*$_5$ (600 MHz, 300 K).

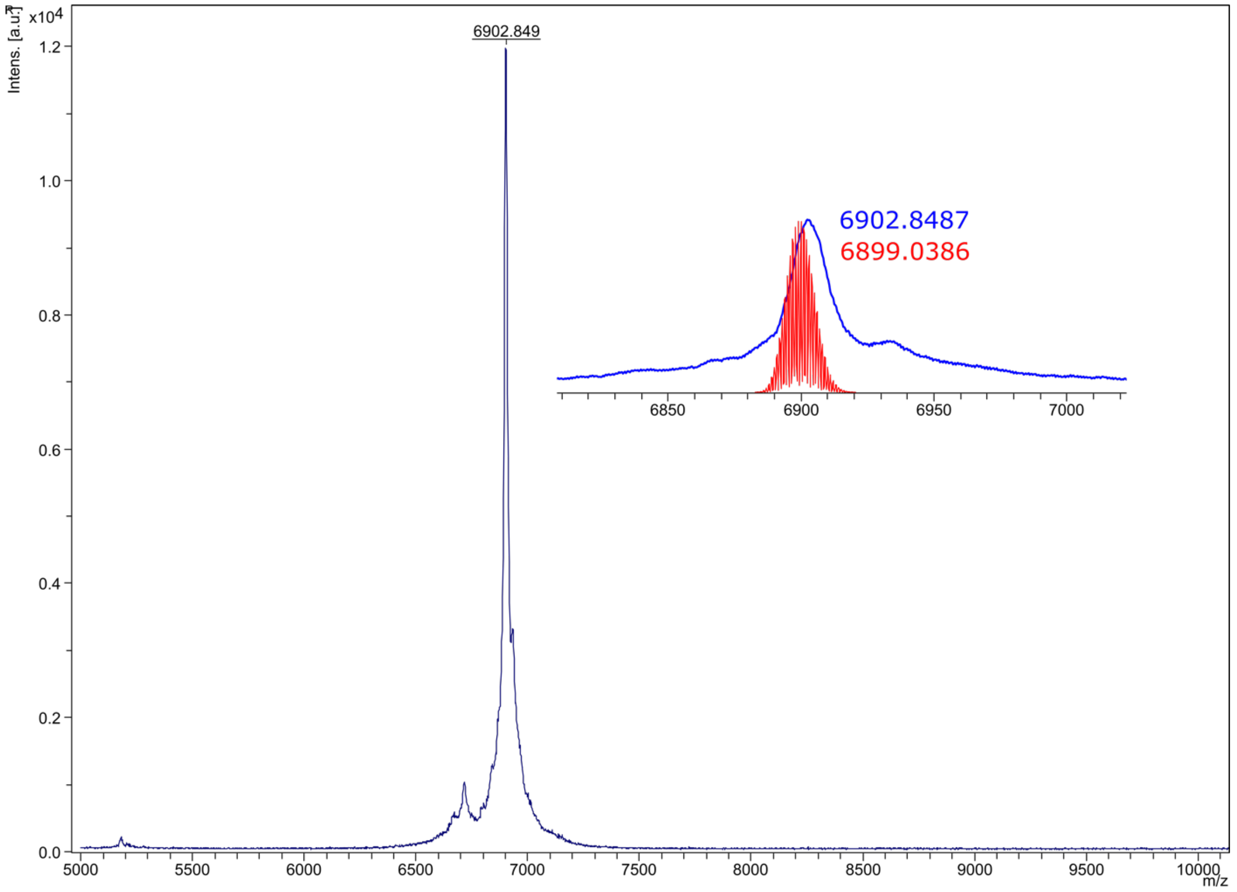

Figure S60. MALDI mass spectrum of **8** together with simulated isotopic pattern (in red), $[M]^+$.

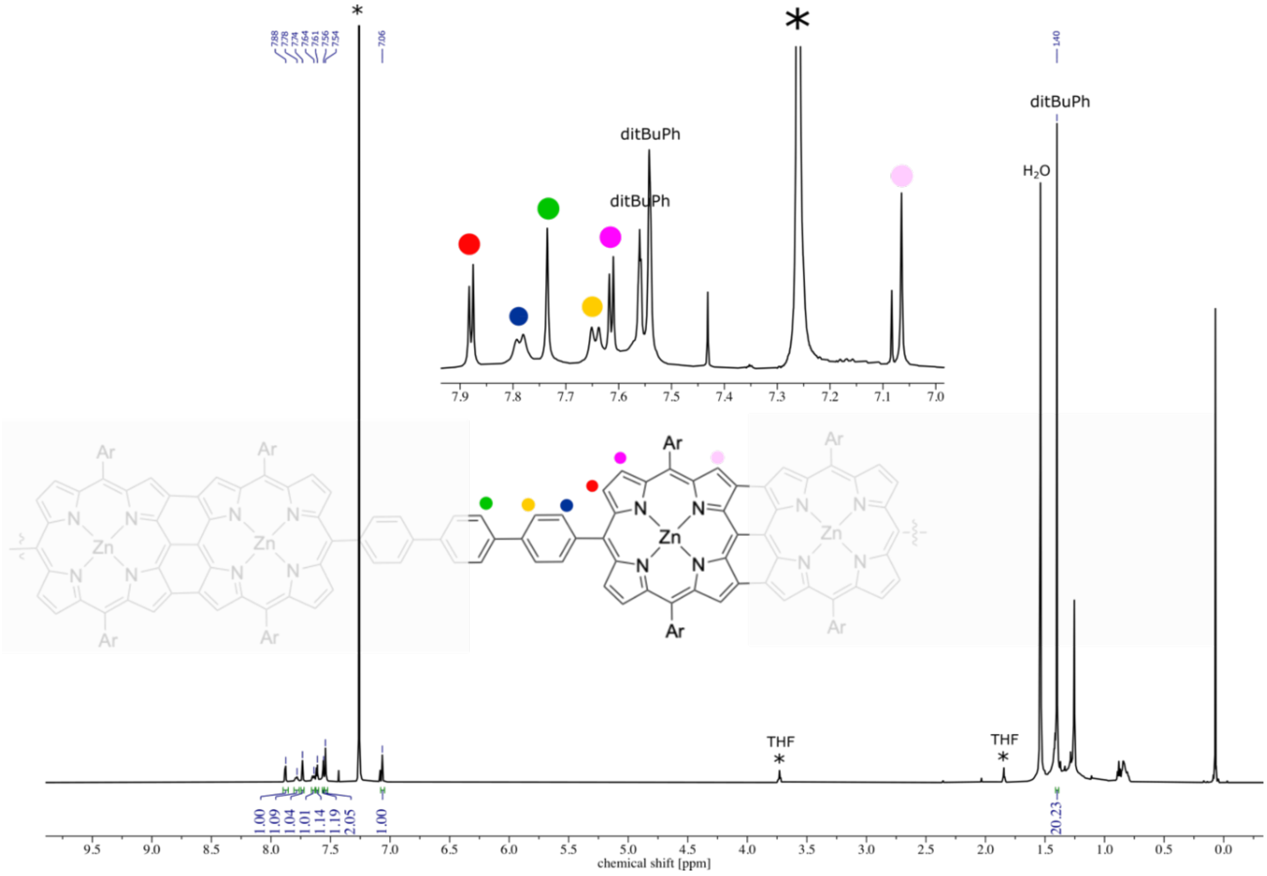

Figure S61. ^1H NMR spectrum of **9** in $CDCl_3$ (600 MHz, 300 K).

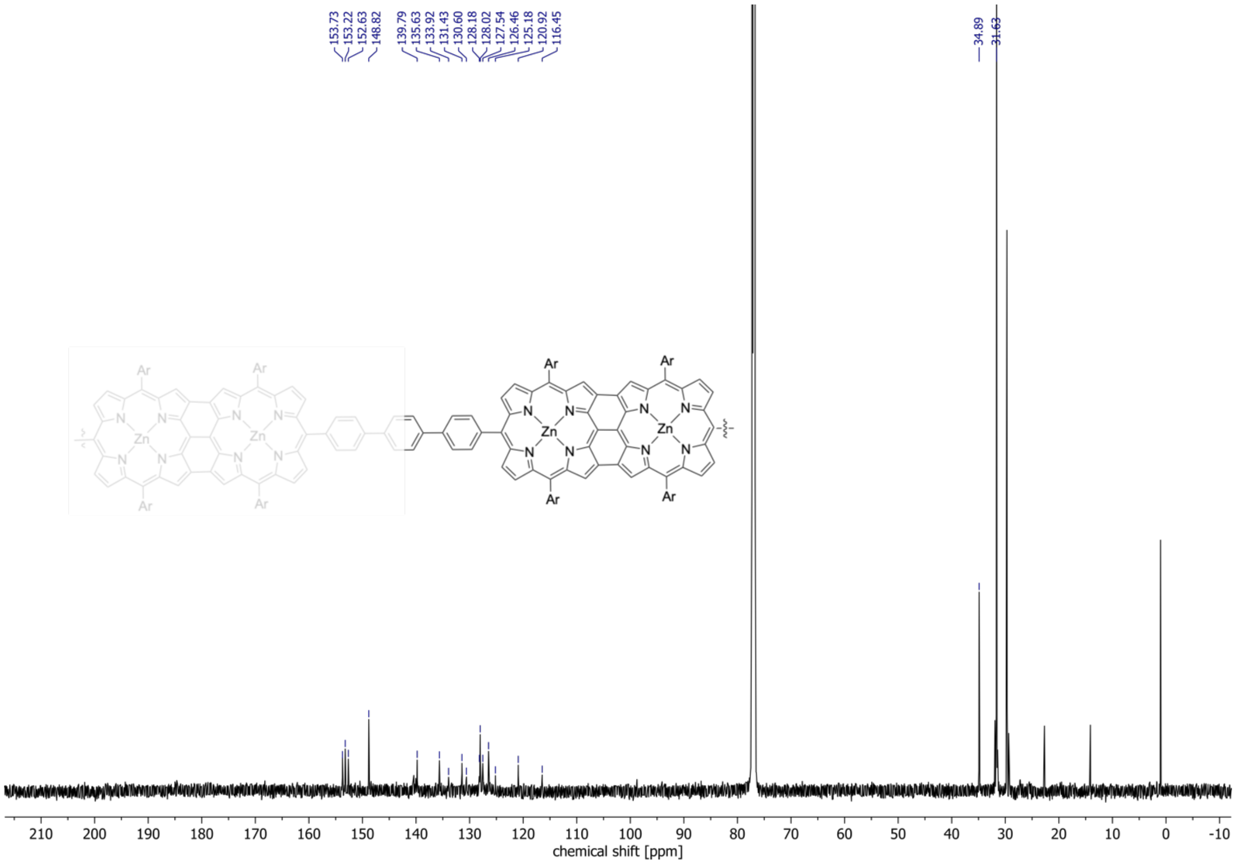

Figure S62. ^{13}C NMR spectrum of **9** in $CDCl_3$ (151 MHz, 300 K). Not all signals are visible (18, should be 23) due to low solubility and aggregation. Signals corresponding to compound are ticked.

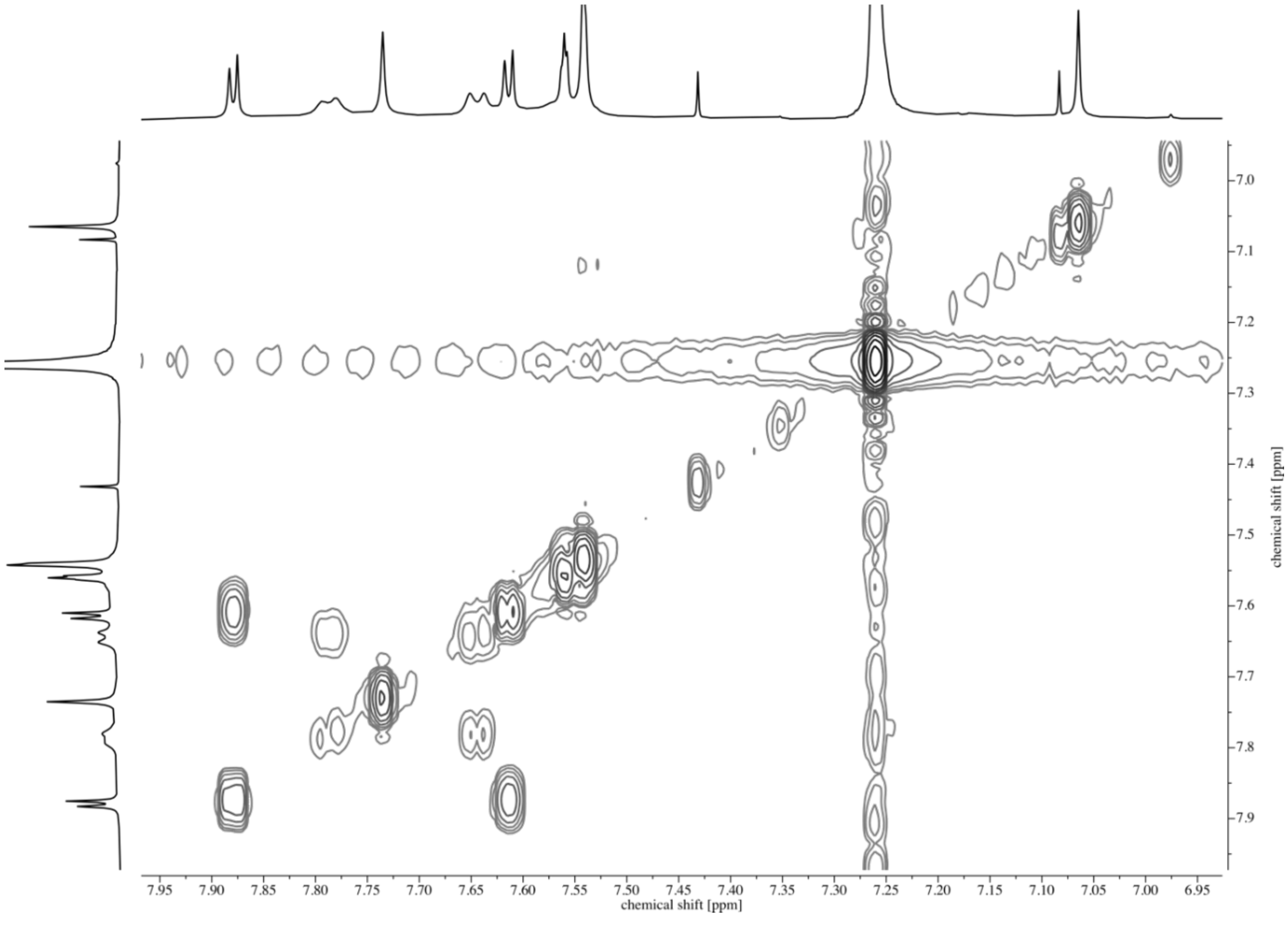

Figure S63. Selected region of a ^{1}H-^{1}H COSY NMR spectrum of **9** in $CDCl_3$ (600 MHz, 300 K).

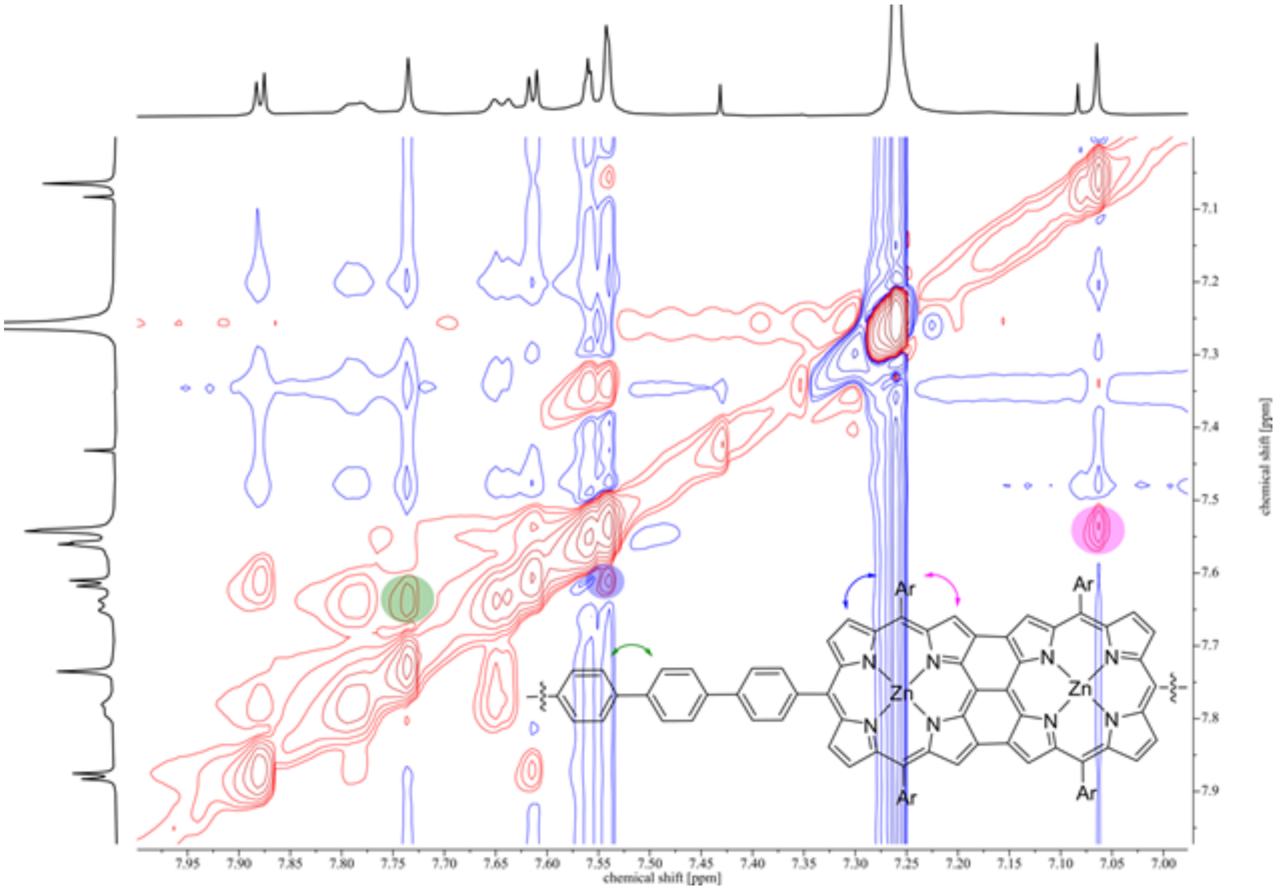

Figure S64. Selected region of a ^{1}H-^{1}H NOESY NMR spectrum of **9** in $CDCl_3$ (600 MHz, 300 K) with marked correlations crucial for signals assignment.

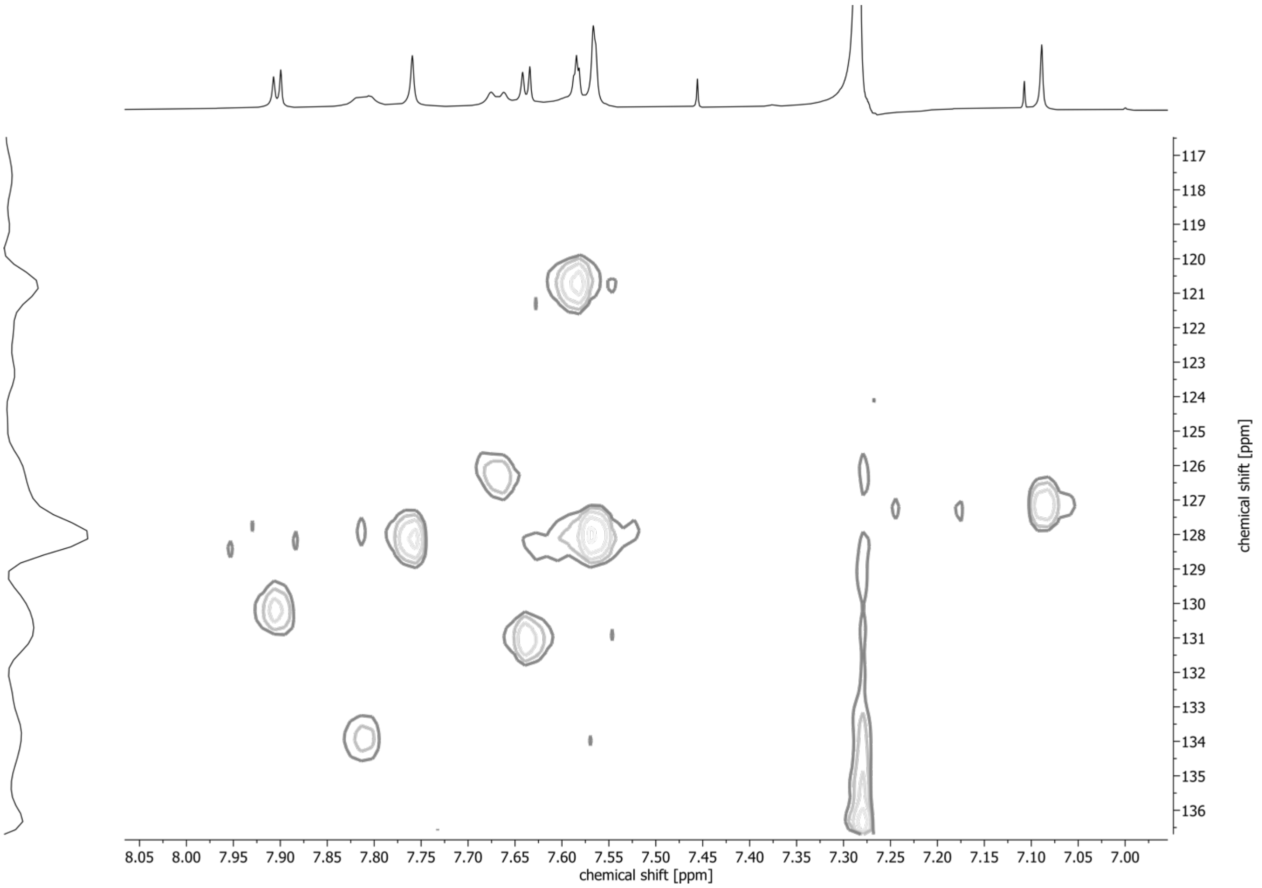

Figure S65. Selected region of a ^{1}H-^{13}C HSQC NMR spectrum of **9** in $CDCl_3$ (600 MHz, 300 K).

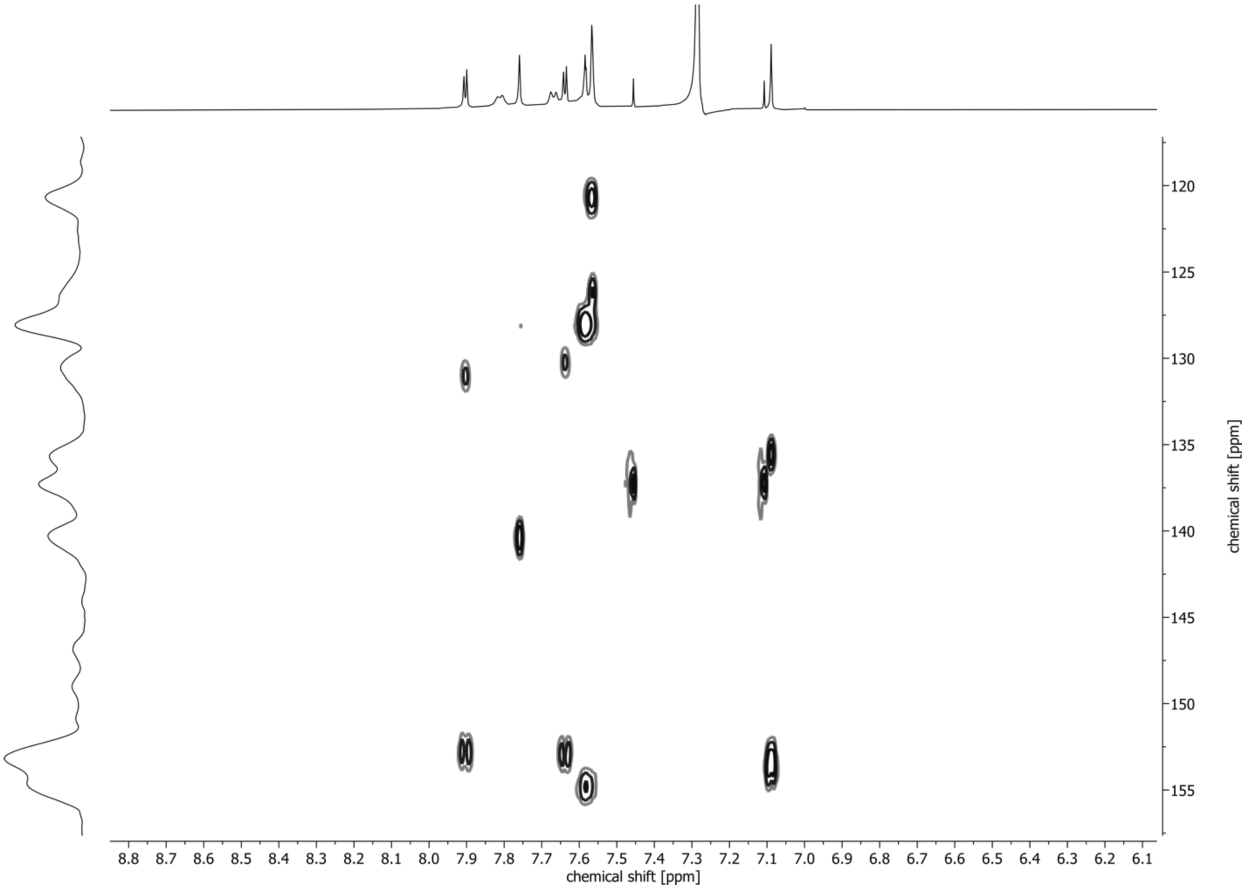

Figure S66. Selected region of a ^{1}H-^{13}C HMBC NMR spectrum of **9** in $CDCl_3$ (600 MHz, 300 K).

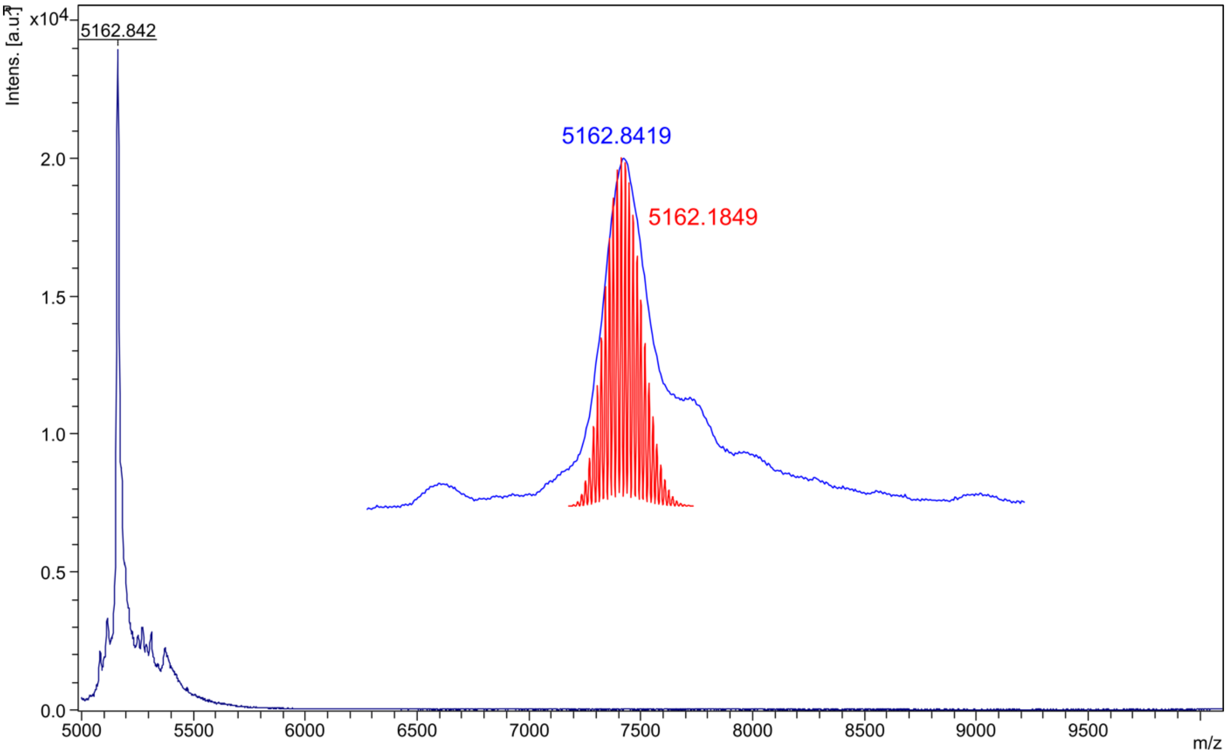

Figure S67. MALDI mass spectrum of **9** together with simulated isotopic pattern (in red), $[M]^+$.

4. Unsuccessful Attempts at Aromatization, Cyclization and Fusion Reactions

In this section, we briefly report our attempts to aromatize compounds **3a**, **3b**, **3c**, **3cNi**, **5a**, **5b**, **5c**, **6a**, **6b** and **6c** that were unsuccessful, in case this may be helpful for Readers working with similar systems.

a) Aromatization of a cyclization substrate

Several methods were tested to aromatize **3c**. First, reaction with $SnCl_2$ dihydrate in the presence of HCl was attempted. Stirring with 1.1 equiv. of $SnCl_2$ and 2.2 equiv. of HCl in THF, followed by quenching with NaOH or triethylamine, extraction and re-insertion of zinc(II) which was demetalated due to presence of acid provided a mixture that was analyzed. ^{1}H NMR spectrum shows a mixture of products. One set of signals, with relatively low intensity, is the desired aromatized product. The other set of signals is asymmetrical (two meso protons with equal intensity) and also shows no signals in the cyclohexadienyl region. The observations, together with a recorded mass spectrum suggesting loss of one oxygen atom, probably indicate an acid-mediated dienol-phenol-like rearrangement.

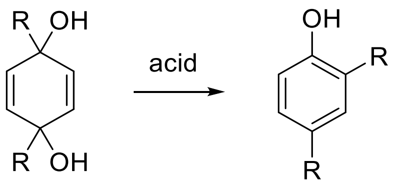

Scheme S2. Postulated side reactivity.

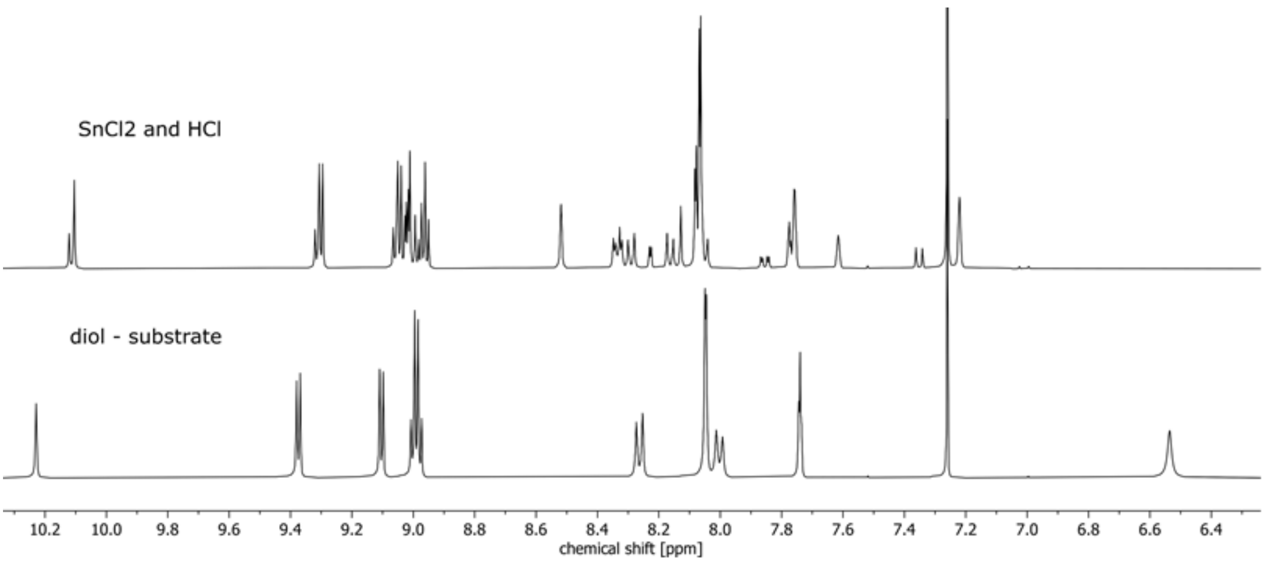

Figure S68. ^{1}H NMR spectra of a reaction mixture after reaction of **3c** with $SnCl_2$ dihydrate after re-insertion of zinc(II) and the substrate for comparison, $CDCl_3$ (upper spectrum with addition of pydirine-d$_5$), 600 MHz, 300 K.

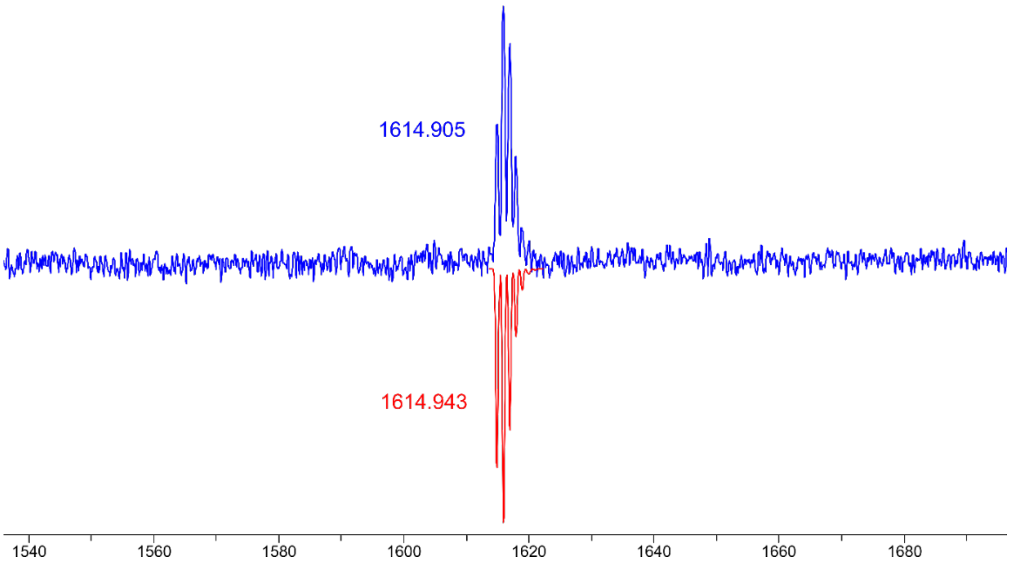

Figure S69. Selected region of a MALDI mass spectrum of a reaction mixture after reaction of **3c** with $SnCl_2$ dihydrate and HCl (before re-metalation) and comparison to the mass corresponding to a loss of one oxygen atom and rearrangement.

Changing the temperature was found to not have an impact on the final result (0 °C, 20 °C, or 50 °C). Changing the amount of $SnCl_2$ and HCl also did not change significantly the observed reactivity.

Other variants of the reaction with $SnCl_2$ were tested. The NMR spectra together with indicated reagents are shown below, on Fig. S70. Similarly, they show mostly reactivity that suggests rearrangement. The highest ratio of the desired aromatized product to the by-products was achieved when $SnCl_2$ (3.5 equiv.) was used without any acid added, by stirring in THF for 2 h at 50 °C.

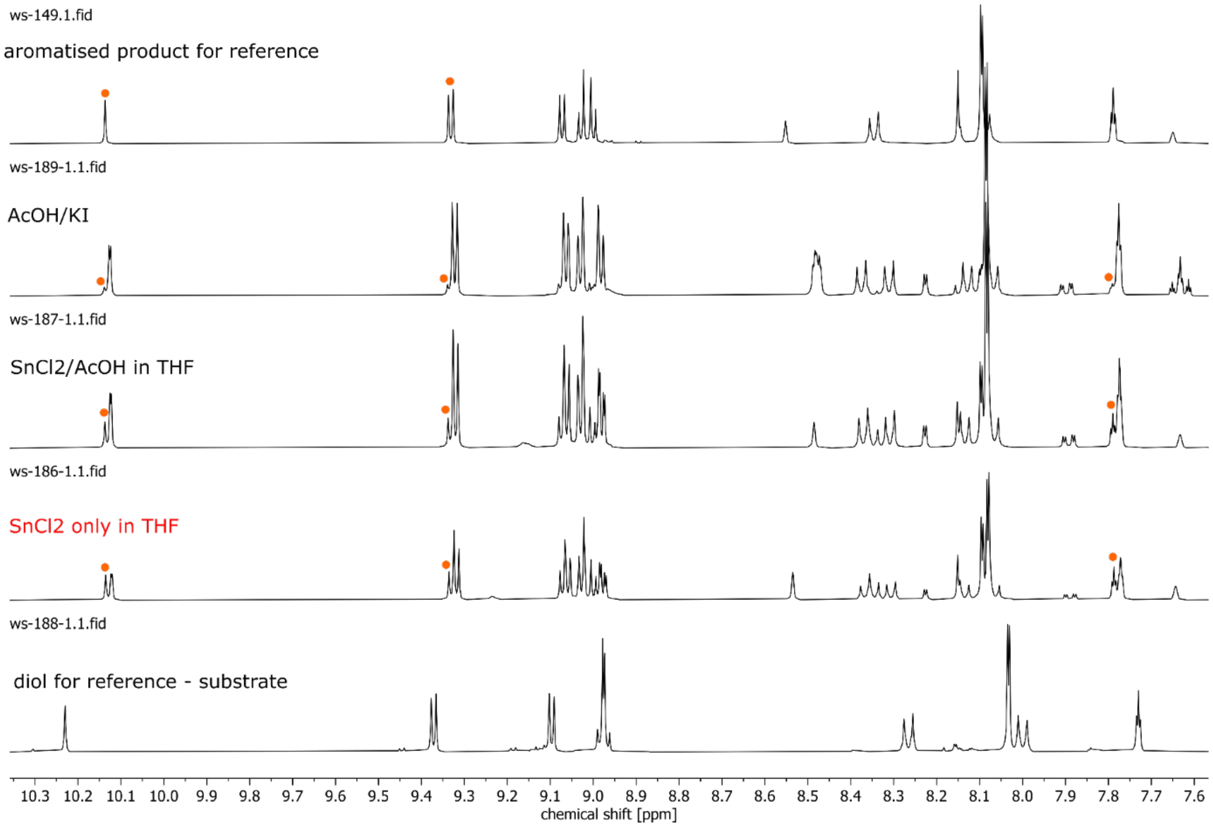

Figure S70. ^1H NMR spectra of reaction mixtures after aromatization attempts with **3c** using different conditions. $CDCl_3$ + 1% pyr-d_5, 600 MHz, 300 K. Orange dots indicate part of signals corresponding to the desired aromatized product.

Reactivity of methylated cyclization substrate **3b** in the reaction with $SnCl_2$ or with $SnCl_2$ in the presence of HCl was found to be similar to **3c**, but the reaction in general tends to occur slower.

When cyclization substrates **3a** or **3b** were subjected to reaction with low-valent titanium generated in-situ by reaction of $TiCl_4$ and $LiAlH_4$, color change to green was observed together with appearance of an insoluble material and turning grey.

b) Aromatization of a nickel(II) complex of a deprotected cyclization substrate **3cNi**

Reaction with $SnCl_2$ dihydrate in the presence of HCl and without it was tested. Quenching by addition of aqueous NaOH or triethylamine followed by extraction with DCM and short silica gel plug (DCM) provided a mixture which was then analyzed. ^{1}H NMR spectrum (Fig. S71) is asymmetric, MALDI mass spectrometry shows mass consistent with a loss of only one oxygen atom, consistently with proposed rearrangement (Fig. S72).

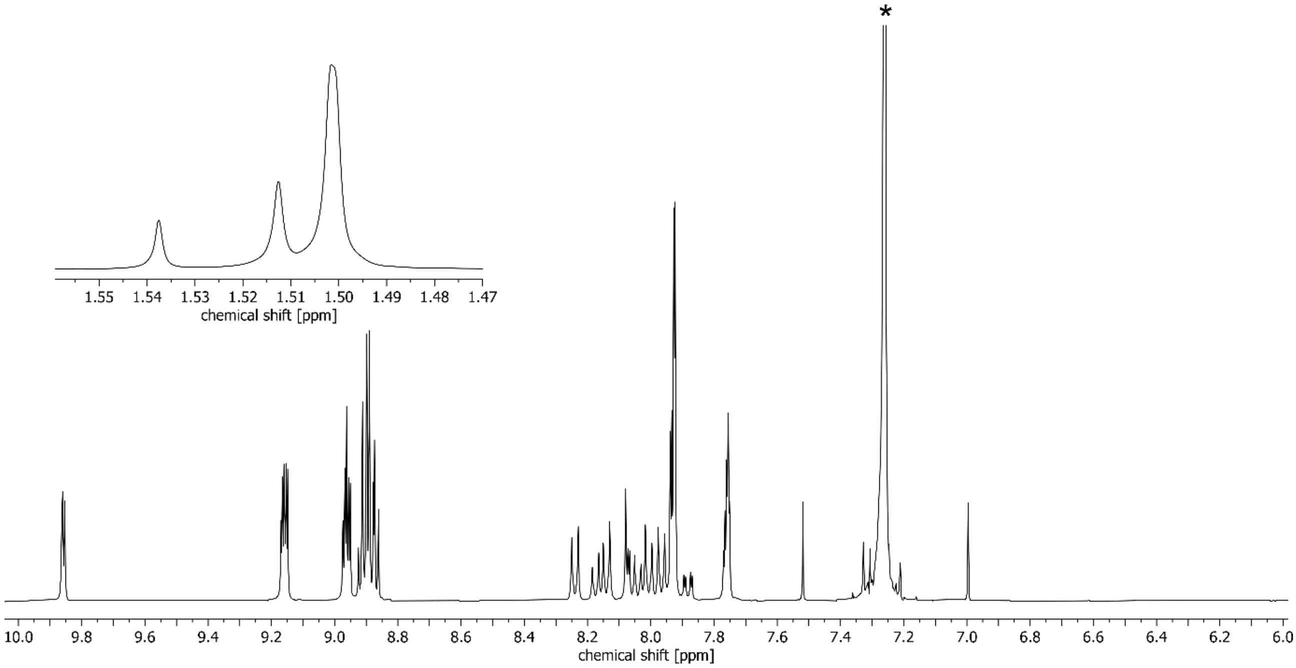

Figure S71. ^{1}H NMR spectrum of a reaction mixture after reaction of **3cNi** with $SnCl_2$ dihydrate, $CDCl_3$ (600 MHz, 300 K).

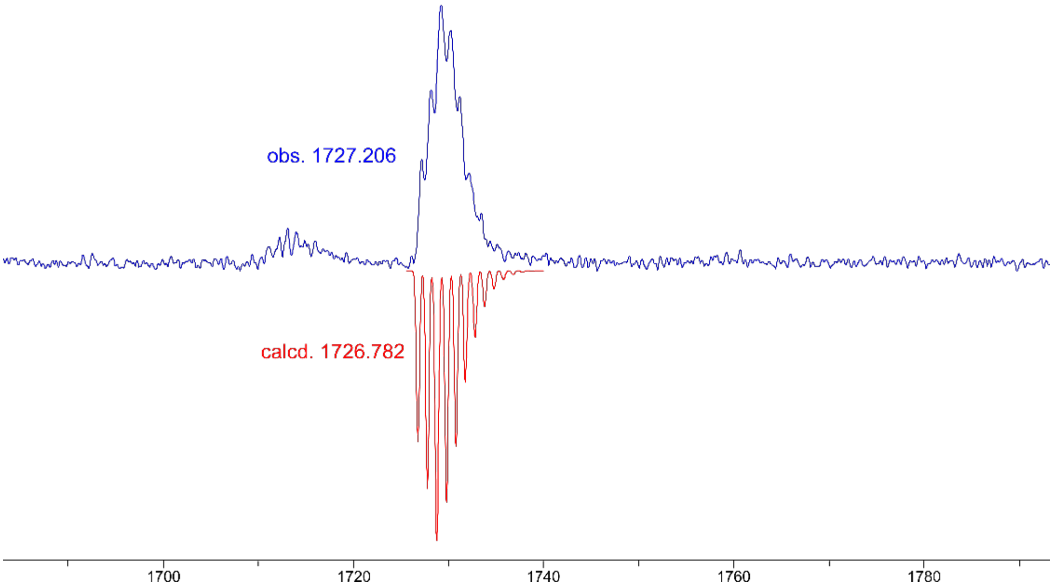

Figure S72. Selected region of a MALDI mass spectrum of a reaction mixture after reaction of **3cNi** with $SnCl_2$ dihydrate and comparison to the mass corresponding to a loss of one oxygen atom and rearrangement.

c) Aromatization of porphyrin hexamer **5c** with $SnCl_2$ in the presence of HCl and without

Asymmetric ^{1}H NMR spectrum is obtained after work-up of the reaction of **5c** with $SnCl_2$ dihydrate (with HCl or without), see Fig. S68. Changing the temperature was found to not have an impact on the final result (0 °C, 20 °C or 50 °C). Work-up involves quenching either with aqueous NaOH and extraction with DCM or quenching with triethylamine without extraction, followed by solvents evaporation, a short silica gel plug (DCM) and re-insertion of zinc(II) by stirring with an excess of zinc(II) acetate in a mixture of DCM and methanol, followed by another short silica gel plug (DCM). Loss of cyclohexadienyl signal was observed and new doublet in the aromatic region appeared at 7.41 ppm with coupling constant of 8.2 Hz, suggesting that it corresponds to phenylene protons (Fig. 73). Seven tert-butyl signals are observed, as opposed to only one for the aromatization substrates **3a-c** and the desired product **7**. The cleanest NMR spectrum is observed when neat $SnCl_2$ dihydrate is used (3.3 equiv.) rather than with HCl.

Analysis of MALDI mass spectrometry results (Fig. S74) suggests loss of three oxygen atoms (observed mass 5224.1, calcd. for the desired aromatized nanoring: 5175.0), consistently with postulated rearrangement suggested for **3c** that in this case, occurs on three rings and that leads to asymmetrical ^{1}H NMR spectrum (Fig. S73 and S75).

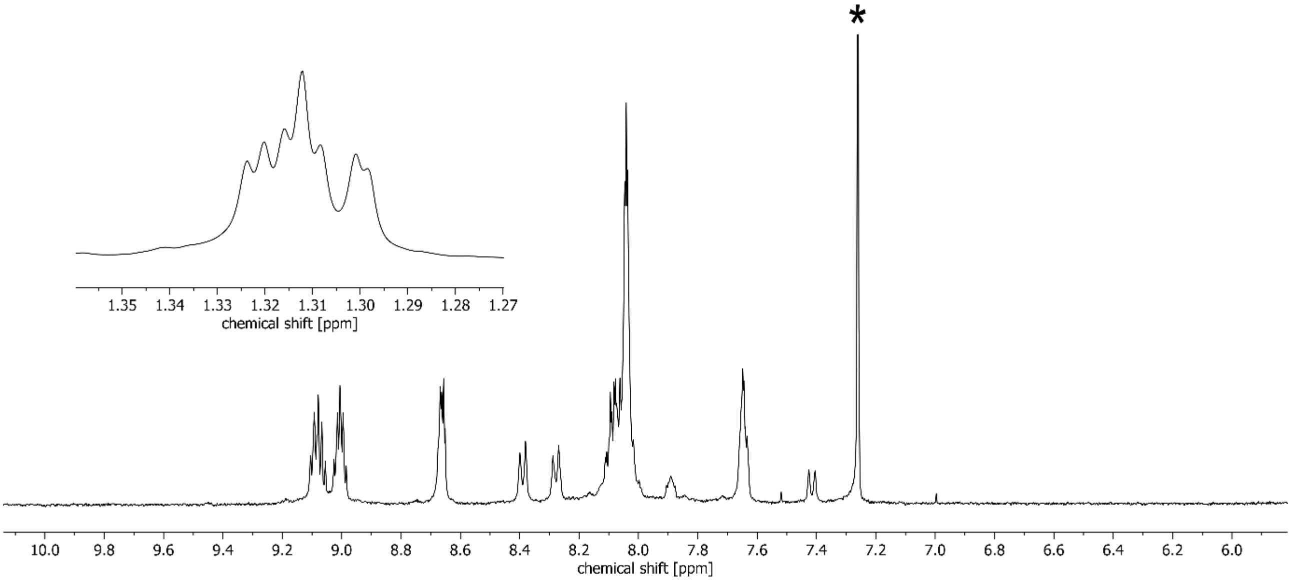

Figure S73. ^{1}H NMR spectrum of a reaction mixture after reaction of **5c** with $SnCl_2$ dihydrate, followed by zinc(II) re-insertion, $CDCl_3$ (600 MHz, 300 K).

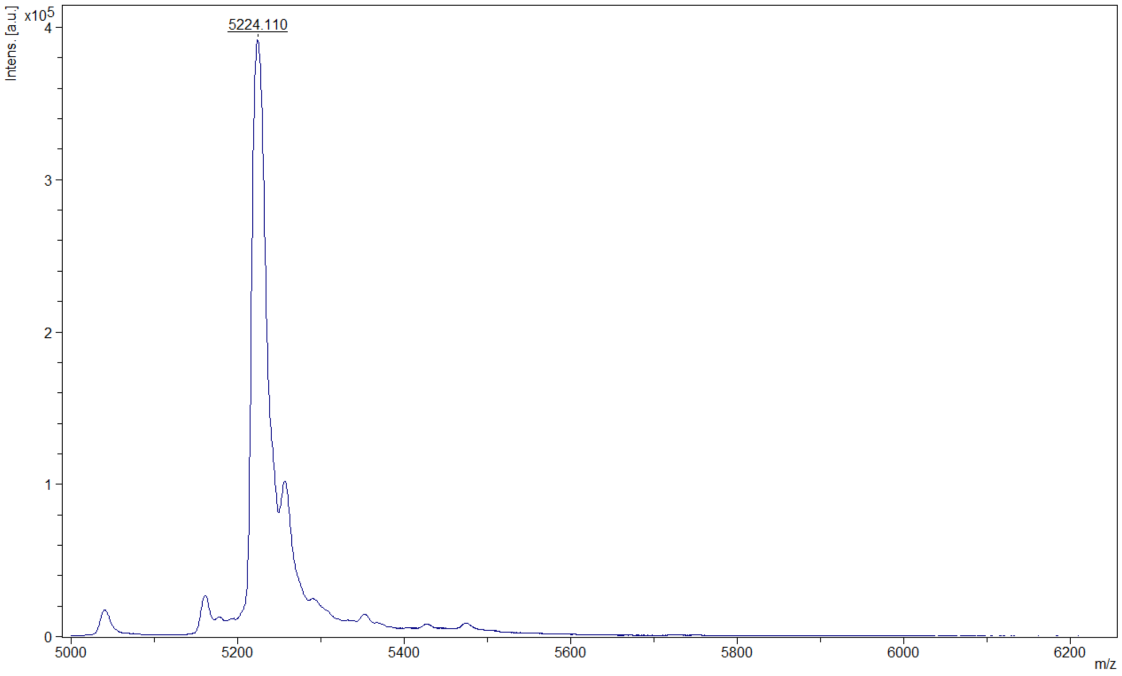

Figure S74. Selected region of a MALDI mass spectrum of a reaction mixture after reaction of **5c** with $SnCl_2$ dihydrate, followed by zinc(II) re-insertion.

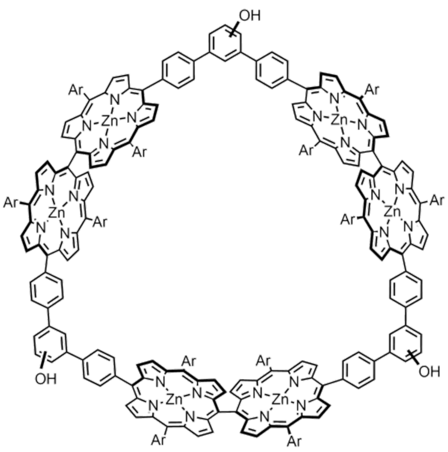

Figure S75. Structure of postulated product(s) from rearrangement during aromatization.

d) Aromatization of porphyrin hexamer **5a**/**5b** with sodium naphthalenide, sodium anthracenide or LiDBB

When reactions with the reducing agents above were performed in THF at –78 °C, followed by addition of I_2, complicated and broad NMR spectra were observed. Change of the reaction time (from 15 min to 2 h), temperature of addition of I_2 (–78 °C, 0 °C or 20 °C) does not lead to formation of the desired products. Quenching with methanol instead of I_2 is similarly unsuccessful.

e) Aromatization of porphyrin octamer **6a**/**6b** with sodium naphthalenide, sodium anthracenide or LiDBB

Similar observations to when **5a**/**5b** were used.

f) Aromatization of porphyrin octamer **6c** with $SnCl_2$ in the presence of HCl and without

6c was tested as a mixture with **5c**. It was unsuccessful similarly to when **5c** was used, we could not see mass of the desired product in MALDI experiments.

g) Aromatization of porphyrin hexamer **5b** with $SnCl_2$ in the presence of HCl and without

Reaction is unsuccessful similarly to the case when hydroxy derivative **5c** was used. NMR spectrum obtained after re-insertion of zinc(II) and silica gel plug is asymmetrical and disappearance of cyclohexadiene motif is observed, indicating rearrangement (Fig. S76).

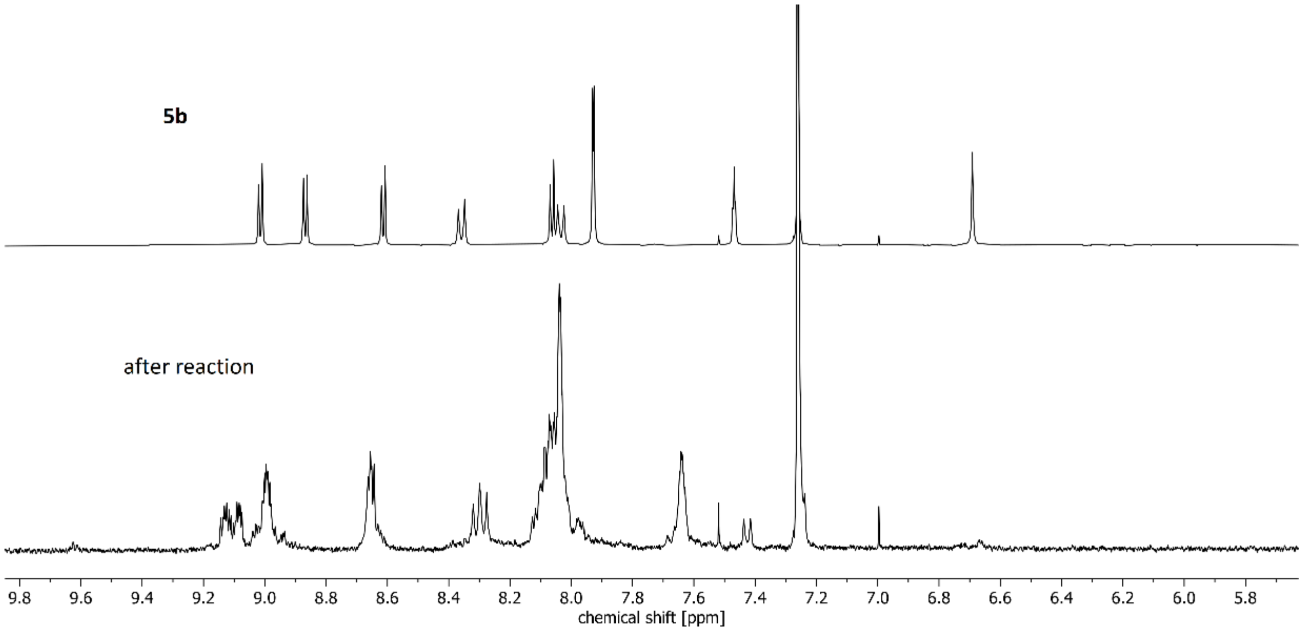

Figure S76. ^1H NMR spectrum of a reaction mixture after reaction of **5b** with $SnCl_2$ dihydrate, followed by zinc(II) re-insertion, $CDCl_3$ (500 MHz, 300 K).

h) Aromatization of porphyrin hexamer **5a/5a** with $LiAlH_4$ and $TiCl_4$

Similarly to the cyclization substrates **3a** and **3b**, when the nanoring is added to a solution of in-situ generated low-valent titanium, color changes to green and decomposition is observed, manifested by precipitation of an insoluble material few minutes after starting the reaction.

i) Cyclization on a nickel(II) complex **3aNi**

When **3aNi** was subjected to the cyclization conditions at –40 °C, no changes were observed even after 6h. Increasing the temperature to 0 °C and room temperature led to formation of some oligomers (monitored with analytical GPC) but disappearance of the cyclohexadienyl signals was observed (see Fig. S77).

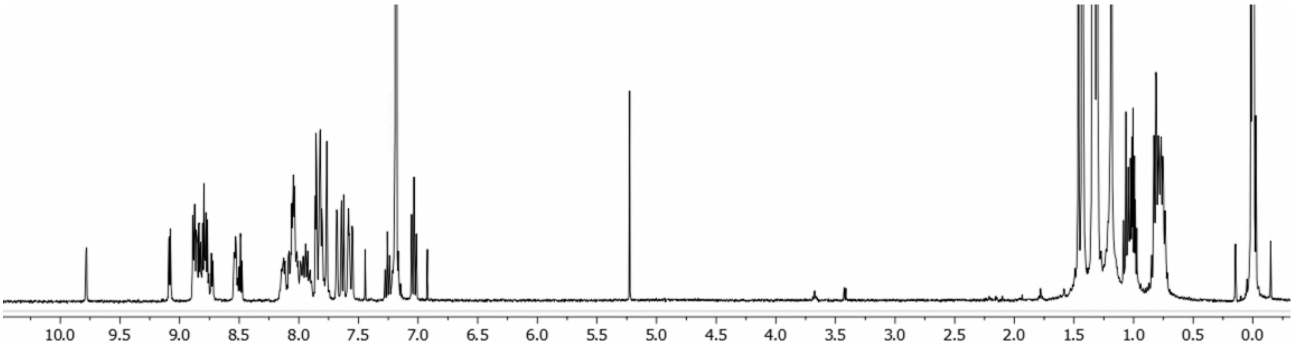

Figure S77. Selected region of a ^{1}H spectrum of a reaction mixture after cyclization attempt of **3aNi** with PIFA at 0 °C.

j) beta-beta Fusion on porphyrin octamer **8**

Asymmetrical ^{1}H NMR spectrum was observed, indicating that fusion of all porphyrins was not achieved (Fig. S78). Solubility has dropped drastically, which is reflected by the signal intensity well below the ^{13}C satellites of $CDCl_3$.

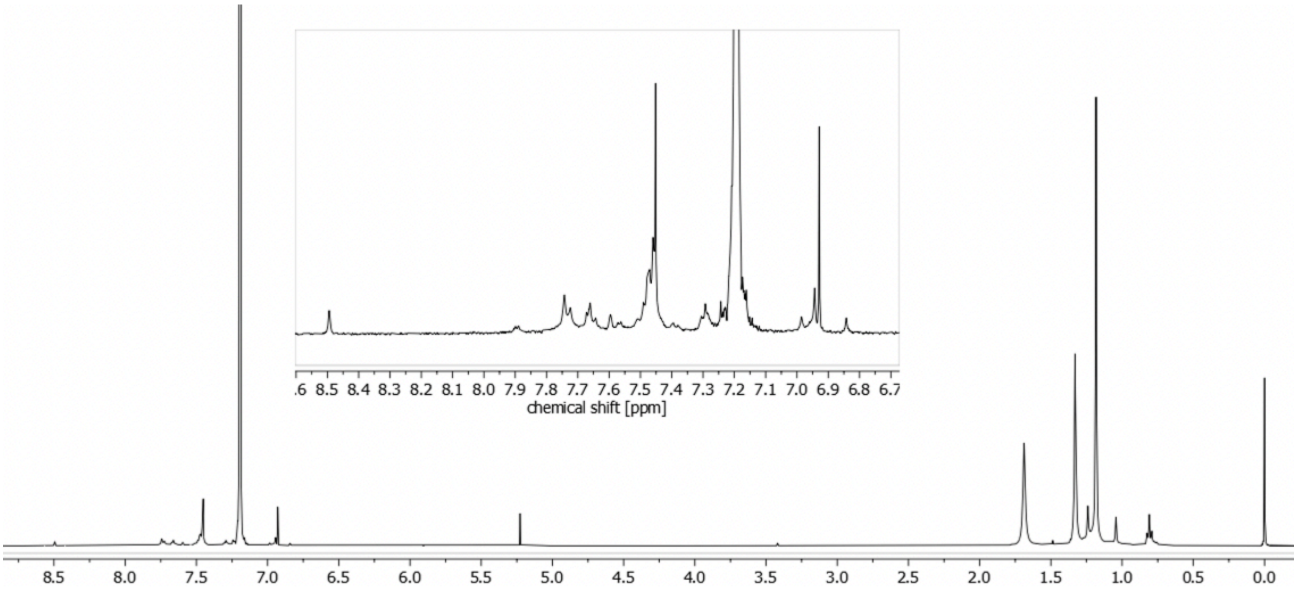

Figure S78. ^{1}H spectrum of a reaction mixture after fusion attempt for **8**, $CDCl_3$ (600 MHz, 300 K).

5. X-ray Crystallography

Single crystals of the compounds **3a**, **5b** and **6b** were successfully prepared. Despite several attempts, crystals of nanorings after aromatization were not obtained in any condition that we tested, similarly after the fusion reaction. **3a** was crystallized by slow evaporation of a solution in a mixture of chloroform and methanol with addition of a drop of pyridine in a vial. Without addition of pyridine, it gave very thin plates that did not diffract. **5b** was crystallized by vapor diffusion of methanol into a solution in toluene. **6b** was crystallized by slow evaporation of a solution in a mixture of chloroform and methanol. During each crystallization, ca. 1 mg of material was used in a volume of ca. 0.6 mL. Crystals were measured at National Crystallography Service, UK.[4]

3a – CCDC 2222978

Single dark red lath-shaped crystals of **3a** were recrystallized from a mixture of chloroform and methanol with addition of a small amount (ca. 0.5 - 1%) of pyridine. A suitable crystal with dimensions 0.22 × 0.06 × 0.01 mm^3 was selected and mounted on a MITEGEN holder in perfluoroether oil on a Rigaku 007HF diffractometer equipped with HF Varimax confocal mirrors and an AFC11 goniometer and HyPix 6000HE detector. The crystal was kept at a steady T = 100(2) K during data collection. The structure was solved with the **ShelXT** 2018/2 (Sheldrick, 2018) solution program using dual methods and by using **Olex2** 1.3 (Dolomanov et al., 2009)[5] as the graphical interface. The model was refined with **ShelXL** 2018/3 (Sheldrick, 2015)[6] using full matrix least squares minimization on F^2.

_refine_special_details: There are areas of highly disordered solvent which required solvent masking to be used. There is disorder of the coordinated pyridine and an ethyl group. As such various restraints (SADI, DFIX, DANG, SIMU and RIGU) have been used

_exptl_absorpt_process_details: CrysAlisPro 1.171.41.105a (Rigaku Oxford Diffraction, 2021) Empirical absorption correction using spherical harmonics, implemented in SCALE3 ABSPACK scaling algorithm.

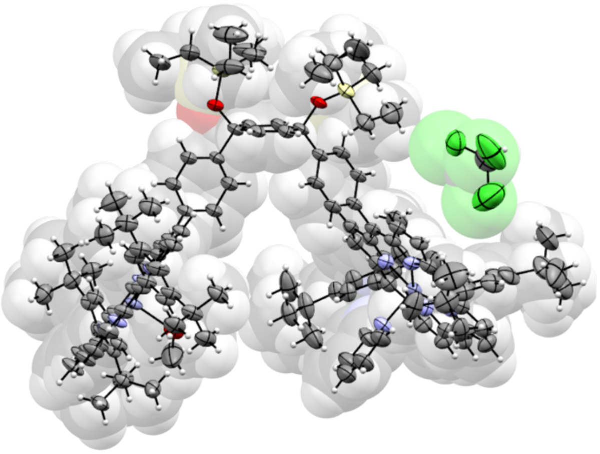

Figure S78. X-ray molecular structure of **3a**. One of the zinc(II) centers has coordinated pyridine, the other one methanol. Thermal ellipsoids are shown at 50% probability level.

Compound	**3a**	**5b**	**6b**
CCDC	2222978	2222979	2222980
Formula	$C_{140}Cl_9H_{176}N_9O_8Si_2Zn_2$	$C_{417}H_{450}N_{24}O_{12}Zn_6$	$C_{472}H_{504}N_{32}O_{16}Zn_8$
$D_{calc.}$/ g cm^{-3}	1.242	0.974	0.886
μ/mm^{-1}	2.609	0.766	0.745
Formula Weight	2618.86	6382.2	7404
Colour	dark red	black	black
Shape	lath-shaped	block-shaped	block-shaped
Size/mm^3	0.22×0.06×0.01	0.210×0.190×0.120	0.360×0.220×0.140
T/K	100(2)	100(2)	100(2)
Crystal System	monoclinic	trigonal	tetragonal
Space Group	$I2/a$	R-3c	$I4_1/a$
a/Å	35.9450(4)	50.0713(3)	49.2172(6)
b/Å	21.0751(3)	50.0713(3)	49.2172(6)
c/Å	37.0027(6)	60.131(3)	22.9225(4)
α/°	90	90	90
β/°	91.4530(10)	90	90
γ/°	90	120	90
V/Å^3	28022.2(7)	130559(6)	55525.9(17)
Z	8	12	4
Z'	1	0.333333	0.25
Wavelength/Å	1.54178	1.54178	1.54178
Radiation type	Cu K$_\alpha$	Cu K$_\alpha$	Cu K$_\alpha$
Θ_{min}/°	2.413	1.765	2.126
Θ_{max}/°	68.245	70.067	70.068
Measured Refl's.	147581	263982	187544
Indep't Refl's	25566	27515	26249
Refl's I≥2 σ(I)	17127	17138	17391
$R_{\rm int}$	0.1236	0.0564	0.091
Parameters	1497	1376	1886
Restraints	1729	24	6493
Largest Peak	1.133	0.829	1.313
Deepest Hole	-0.835	-0.425	-0.445
GooF	1.078	1.247	1.408
wR_2 (all data)	0.269	0.3355	0.3685
wR_2	0.2462	0.3063	0.3501
R_1 (all data)	0.117	0.119	0.1414
R_1	0.0893	0.0951	0.1111

Table S1. Crystallographic data.

5b – CCDC 2222979

Single black block-shaped crystals of **5b** were grown by vapor diffusion of methanol into a solution in toluene. A suitable crystal with dimensions 0.210 × 0.190 × 0.120 mm^3 was selected and mounted on a MITIGEN holder in oil on a Rigaku 007HF diffractometer with HF Varimax confocal mirrors, an UG2 goniometer and HyPix 6000HE detector. The crystal was kept at a steady *T* = 100(2) K during data collection. The structure was solved with the **ShelXT** 2018/2 (Sheldrick, 2018) solution program using dual methods and by using **Olex2** 1.5 (Dolomanov et al., 2009)[5] as the graphical interface. The model was refined with **ShelXL** 2018/3 (Sheldrick, 2015)[6] using full matrix least squares minimization on ***F*²**.

_*refine_special_details*: Solvent masking was employed. The structure shows signs of flexibility for which attempts to model as disorder gave worse results. As such the gross architecture is given with various restraints (DFIX, DANG, FLAT, SADI) employed

_*exptl_absorpt_process_details*: CrysAlisPro 1.171.42.51a (Rigaku Oxford Diffraction, 2022) using spherical harmonics, implemented in SCALE3 ABSPACK scaling algorithm.

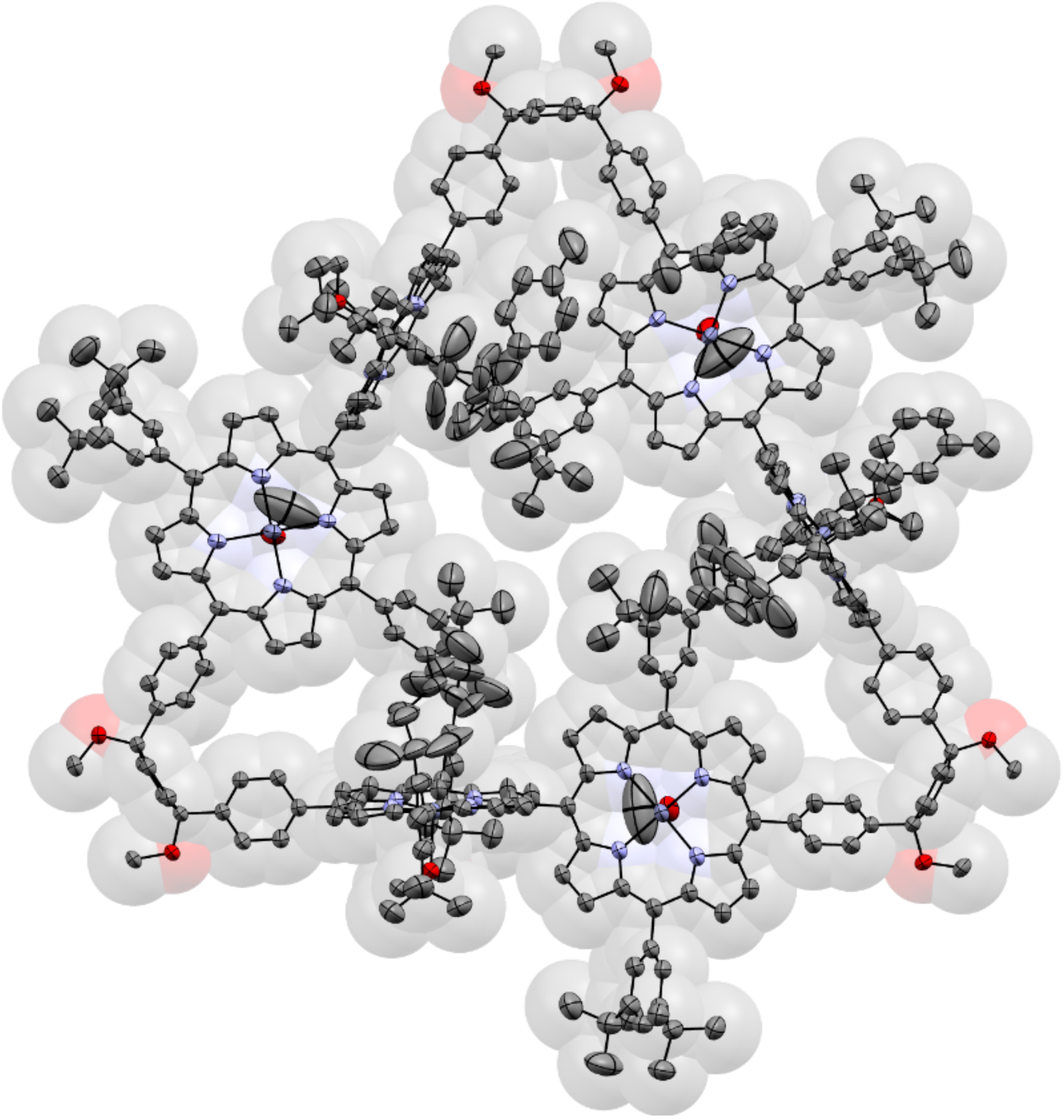

Figure S79. X-ray molecular structure of **5b**. Thermal ellipsoids are shown at 50% probability level. Hydrogen atoms were omitted for clarity.

6b – CCDC 2222980

Single black block-shaped crystals of **6b** were recrystallized from a mixture of chloroform and methanol by slow evaporation. A suitable crystal with dimensions 0.360 × 0.220 × 0.140 mm^3 was selected and mounted on a MITIGEN holder in oil on a Rigaku 007HF diffractometer equipped with Arc)Sec VHF Varimax confocal mirrors and a UG2 goniometer and HyPix 6000HE detector. The crystal was kept at a steady T = 100(2) K during data collection. The structure was solved with the **ShelXT** 2018/2 (Sheldrick, 2018) solution program using dual methods and by using **Olex2** 1.5 (Dolomanov et al., 2009)[5] as the graphical interface. The model was refined with **ShelXL** 2018/3 (Sheldrick, 2015)[6] using full matrix least squares minimization on $\boldsymbol{F^2}$.

_refine_special_details: Solvent masking was used (assumed as chloroform). There is significant disorder of multiple aromatic rings and t-butyl groups, generally split into 3 parts. As such various restraints (SADI, DFIX, DANG, FLAT, RIGU, SIMU, SUMP) and constraints (AFIX) were used. The quality of data is not great meaning GOOF not fully optimized resulting in multiple OMIT commands used.

_exptl_absorpt_process_details: CrysAlisPro 1.171.41.105a (Rigaku Oxford Diffraction, 2021) using spherical harmonics, implemented in SCALE3 ABSPACK scaling algorithm.

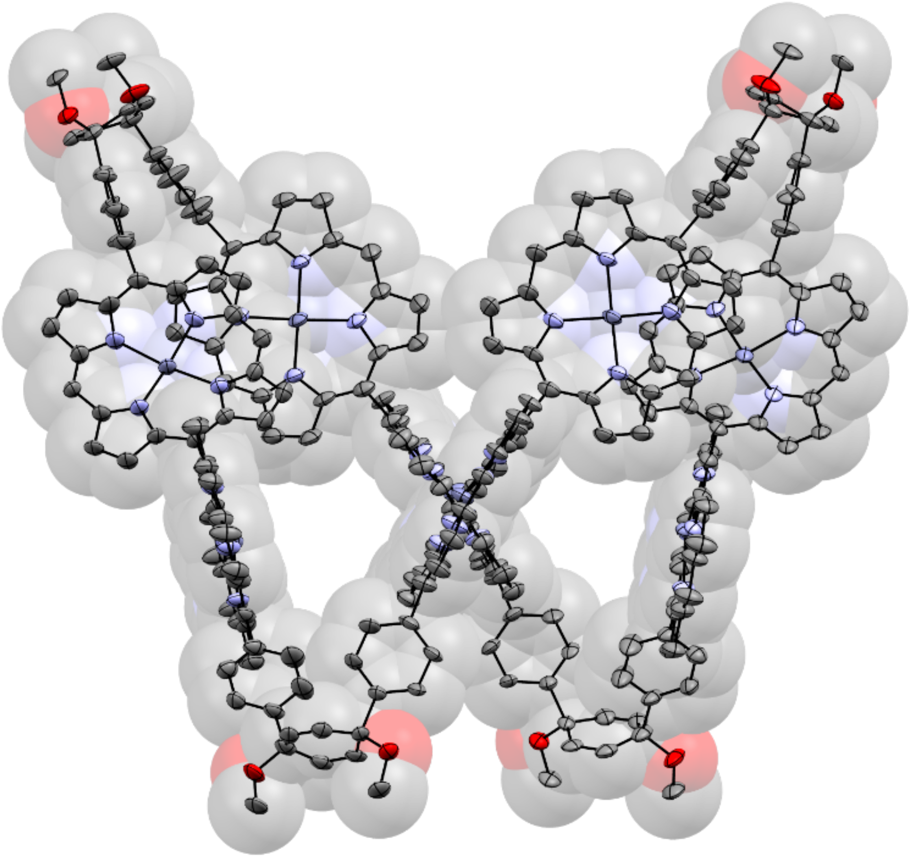

Figure S80. X-ray molecular structure of **6b**. Thermal ellipsoids are shown at 50% probability level. Hydrogen atoms, methanol coordinated to each Zn center and *meso* substituents were omitted for clarity.

6. DFT Calculations

Density functional theory (DFT) calculations were performed using Gaussian 16.[7] DFT geometry optimizations were carried out in unconstrained C1 symmetry, using molecular mechanics or semiempirical models as starting geometries. The calculations were performed using the functional B3LYP and the 6-31G(d,p) basis set.[8] Each structure was optimized to meet standard convergence criteria, and the existence of a local minimum was verified by a normal mode frequency calculation. Negative frequencies were not observed. Kohn-Sham orbitals were visualized using GaussView 6.0.16 software (isovalue 0.02).

6.1. General results

6.1.1. Calculated parameters

reference	SCF E [a.u.]	ZPV E [a.u.]	lowest freq. [cm^{-1}]	H [a.u.]	G [a.u.]	E_{HOMO} [eV]	E_{LUMO} [eV]
**7**	-18677.9037	-18675.658	3.09	-18675.5043	-18675.866	-5.05	-2.32
**8**	-24903.908	-24900.913	1.63	-24900.7076	-24901.188	-5.08	-2.3
**9**	-18670.818	-18668.699	3.14	-18668.5531	-18668.89	-4.73	-3.06
**11**	-24894.4597	-24891.635	1.79	-24891.4388	-24891.887	-4.75	-3.06
**S1**	-6689.31028	-6688.3797	4.27	-6688.31801	-6688.4828		
**S2**	-6686.94842	-6686.0602	4.61	-6686.00102	-6686.1574		

Table S2. From left to right: reference, SCF electronic energy, zero-point vibrational energy, lowest vibrational frequency, sum of electronic and thermal enthalpies, sum of electronic and free energies, energy of the HOMO orbital, energy of the LUMO orbital.

6.1.2. Calculated molecular geometries

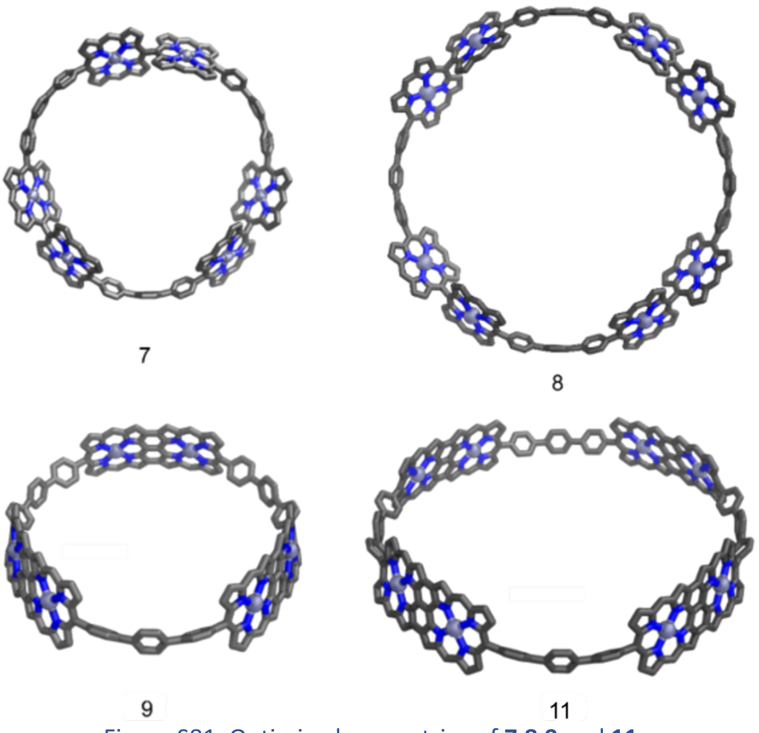

Figure S81. Optimized geometries of **7**,**8**,**9** and **11**.

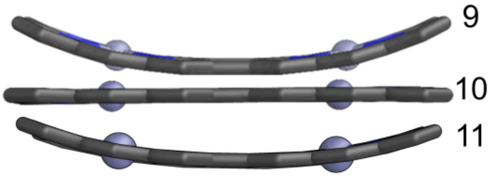

Figure S82. Comparison between planarity of tape fragments in **9**, **10** and **11**.

6.1.3. Cartesian Coordinates

7

C	-0.362000	-14.198000	0.473000
C	0.455000	-14.862000	-0.462000
N	1.822000	-14.736000	-0.561000
C	2.224000	-15.579000	-1.568000
C	3.532000	-15.700000	-2.043000
C	4.639000	-14.950000	-1.646000
C	5.941000	-15.004000	-2.267000
H	6.223000	-15.685000	-3.060000
C	6.700000	-14.036000	-1.682000
H	7.726000	-13.772000	-1.896000
C	5.874000	-13.402000	-0.677000
C	6.284000	-12.343000	0.154000
C	5.517000	-11.836000	1.223000
C	5.955000	-10.813000	2.152000
H	6.909000	-10.309000	2.121000
C	4.949000	-10.635000	3.052000
C	3.875000	-11.513000	2.658000
C	2.619000	-11.544000	3.259000
C	1.490000	-12.225000	2.805000
N	1.450000	-13.060000	1.715000
C	0.164000	-12.047000	3.339000
C	-0.684000	-12.741000	2.529000
H	-1.759000	-12.805000	2.603000
H	-0.084000	-11.439000	4.199000
H	4.920000	-9.957000	3.894000
N	4.245000	-12.240000	1.552000
N	4.629000	-13.986000	-0.666000
C	1.083000	-16.275000	-2.110000
H	1.121000	-17.007000	-2.906000
Zn	3.043000	-13.527000	0.530000
C	-0.014000	-15.820000	-1.440000
H	-1.045000	-16.114000	-1.573000
C	0.127000	-13.388000	1.518000
Zn	10.216000	-9.406000	-0.535000
N	8.507000	-9.806000	-1.566000
C	7.515000	-10.701000	-1.237000
C	6.417000	-10.572000	-2.174000
H	5.502000	-11.143000	-2.146000
C	6.776000	-9.620000	-3.079000
H	6.209000	-9.260000	-3.928000
C	7.562000	-11.611000	-0.161000
C	8.678000	-11.784000	0.677000
C	8.806000	-12.811000	1.689000
H	8.061000	-13.563000	1.903000
C	10.021000	-12.637000	2.280000
H	10.463000	-13.219000	3.079000
C	10.632000	-11.488000	1.656000
C	11.834000	-10.905000	2.057000
C	12.388000	-9.715000	1.580000
C	13.559000	-9.074000	2.126000
H	14.168000	-9.470000	2.928000
C	13.718000	-7.900000	1.452000
C	12.660000	-7.830000	0.467000
C	12.501000	-6.794000	-0.473000
C	11.562000	-6.817000	-1.526000
C	11.415000	-5.796000	-2.543000
C	10.398000	-6.188000	-3.359000
C	9.884000	-7.423000	-2.823000
C	8.733000	-8.062000	-3.283000
H	10.002000	-5.674000	-4.225000
H	12.008000	-4.898000	-2.617000
H	14.489000	-7.154000	1.586000
C	8.071000	-9.129000	-2.680000
N	9.809000	-11.001000	0.669000
N	10.619000	-7.800000	-1.726000
N	11.866000	-8.949000	0.566000
H	3.691000	-16.412000	-2.847000
H	2.482000	-10.906000	4.128000
H	12.365000	-11.396000	2.867000
H	8.256000	-7.629000	-4.157000
C	13.267000	-5.523000	-0.275000
C	14.187000	-5.016000	-1.206000
H	14.486000	-5.632000	-2.049000
C	14.691000	-3.721000	-1.085000
H	15.389000	-3.354000	-1.832000
C	14.290000	-2.879000	-0.034000
C	13.446000	-3.427000	0.948000
H	13.103000	-2.805000	1.768000
C	14.592000	-1.425000	-0.010000
C	14.652000	-0.687000	-1.205000
C	14.659000	0.704000	-1.195000
C	14.592000	1.425000	0.010000
C	14.290000	2.878000	0.034000
C	13.446000	3.426000	-0.948000
C	12.951000	4.720000	-0.833000
H	12.248000	5.092000	-1.572000
C	13.267000	5.522000	0.275000
C	14.187000	5.016000	1.206000
H	14.486000	5.631000	2.049000
H	13.103000	2.804000	-1.768000
C	14.691000	3.721000	1.085000
H	15.390000	3.354000	1.832000
C	14.652000	0.686000	1.205000
H	14.617000	1.205000	2.158000
C	14.658000	-0.704000	1.195000
H	14.669000	-1.239000	2.140000
C	12.951000	-4.720000	0.833000
H	12.248000	-5.092000	1.572000
H	14.669000	1.238000	-2.140000
H	14.617000	-1.206000	-2.158000
C	12.501000	6.793000	0.473000
C	12.660000	7.830000	-0.467000
N	11.867000	8.949000	-0.566000
C	12.389000	9.715000	-1.580000
C	11.834000	10.904000	-2.057000
C	10.633000	11.488000	-1.656000
C	10.022000	12.637000	-2.280000
H	10.464000	13.218000	-3.079000
C	8.806000	12.810000	-1.689000
H	8.061000	13.563000	-1.903000
C	8.679000	11.784000	-0.677000
C	7.562000	11.611000	0.161000
C	7.516000	10.701000	1.237000
C	6.418000	10.571000	2.174000
H	5.503000	11.143000	2.146000
C	6.776000	9.620000	3.079000
C	8.072000	9.129000	2.680000
C	8.733000	8.061000	3.283000
C	9.884000	7.422000	2.823000
N	10.619000	7.799000	1.726000
C	10.398000	6.188000	3.359000
C	11.416000	5.796000	2.543000
H	12.008000	4.897000	2.617000
H	10.002000	5.674000	4.225000
H	6.210000	9.260000	3.928000
N	8.507000	9.806000	1.566000
N	9.810000	11.001000	-0.669000
C	13.559000	9.073000	-2.126000
H	14.168000	9.470000	-2.928000
Zn	10.217000	9.406000	0.535000
C	13.719000	7.899000	-1.452000
H	14.489000	7.154000	-1.586000
C	11.562000	6.817000	1.526000
Zn	3.043000	13.527000	-0.530000
N	4.245000	12.240000	-1.552000
C	5.518000	11.835000	-1.223000
C	5.956000	10.812000	-2.152000
H	6.910000	10.309000	-2.121000

C	4.950000	10.635000	-3.052000
H	4.921000	9.957000	-3.894000
C	6.285000	12.343000	-0.154000
C	5.875000	13.402000	0.677000
C	6.701000	14.036000	1.682000
H	7.726000	13.772000	1.896000
C	5.941000	15.004000	2.267000
H	6.223000	15.685000	3.060000
C	4.639000	14.950000	1.646000
C	3.532000	15.700000	2.043000
C	2.225000	15.579000	1.568000
C	1.083000	16.275000	2.110000
H	1.122000	17.007000	2.906000
C	-0.013000	15.820000	1.440000
C	0.456000	14.862000	0.462000
C	-0.362000	14.198000	-0.473000
C	0.128000	13.388000	-1.518000
C	-0.683000	12.741000	-2.529000
C	0.164000	12.047000	-3.339000
C	1.490000	12.225000	-2.805000
C	2.620000	11.544000	-3.259000
H	-0.083000	11.439000	-4.199000
H	-1.758000	12.805000	-2.603000
H	-1.044000	16.114000	1.573000
C	3.876000	11.513000	-2.658000
N	4.630000	13.986000	0.666000
N	1.450000	13.060000	-1.715000
N	1.822000	14.736000	0.561000
H	12.365000	11.395000	-2.867000
H	8.256000	7.629000	4.157000
H	3.692000	16.412000	2.847000
H	2.483000	10.906000	-4.128000
C	-1.846000	14.229000	-0.276000
C	-2.742000	14.770000	-1.210000
H	-2.356000	15.333000	-2.055000
C	-4.116000	14.561000	-1.092000
H	-4.781000	14.980000	-1.841000
C	-4.648000	13.797000	-0.039000
C	-3.754000	13.342000	0.946000
H	-4.123000	12.738000	1.768000
C	-6.058000	13.333000	-0.016000
C	-6.726000	13.015000	-1.212000
C	-7.935000	12.327000	-1.203000
C	-8.529000	11.912000	0.002000
C	-9.638000	10.925000	0.025000
C	-9.691000	9.920000	-0.956000
C	-10.565000	8.845000	-0.841000
H	-10.537000	8.050000	-1.580000
C	-11.418000	8.719000	0.266000
C	-11.439000	9.769000	1.197000
H	-12.122000	9.721000	2.040000
H	-8.981000	9.933000	-1.776000
C	-10.569000	10.852000	1.076000
H	-10.599000	11.641000	1.823000
C	-7.920000	12.333000	1.197000
H	-8.354000	12.046000	2.150000
C	-6.718000	13.033000	1.188000
H	-6.262000	13.310000	2.133000
C	-2.385000	13.558000	0.833000
H	-1.713000	13.138000	1.575000
H	-8.402000	12.069000	-2.148000
H	-6.258000	13.242000	-2.164000
C	-12.137000	7.421000	0.465000
C	-13.117000	7.042000	-0.473000
N	-13.691000	5.796000	-0.570000
C	-14.618000	5.866000	-1.581000
C	-15.375000	4.792000	-2.056000
C	-15.280000	3.460000	-1.655000
C	-15.974000	2.357000	-2.277000
H	-16.701000	2.451000	-3.073000
C	-15.517000	1.217000	-1.688000

H	-15.799000	0.196000	-1.900000
C	-14.560000	1.618000	-0.679000
C	-13.850000	0.737000	0.157000
C	-13.034000	1.150000	1.229000
C	-12.371000	0.262000	2.164000
H	-12.410000	-0.816000	2.136000
C	-11.721000	1.048000	3.066000
C	-11.943000	2.415000	2.668000
C	-11.346000	3.521000	3.270000
C	-11.368000	4.838000	2.811000
N	-12.065000	5.287000	1.716000
C	-10.554000	5.899000	3.346000
C	-10.725000	6.977000	2.531000
H	-10.243000	7.939000	2.604000
H	-9.908000	5.812000	4.210000
H	-11.123000	0.736000	3.913000
N	-12.751000	2.456000	1.558000
N	-14.445000	2.989000	-0.671000
C	-14.648000	7.201000	-2.127000
H	-15.297000	7.531000	-2.927000
Zn	-13.260000	4.137000	0.528000
C	-13.707000	7.924000	-1.456000
H	-13.446000	8.964000	-1.591000
C	-11.686000	6.595000	1.516000
Zn	-13.261000	-4.137000	-0.528000
N	-12.751000	-2.455000	-1.558000
C	-13.034000	-1.150000	-1.229000
C	-12.371000	-0.262000	-2.164000
H	-12.410000	0.816000	-2.136000
C	-11.721000	-1.047000	-3.066000
H	-11.123000	-0.736000	-3.913000
C	-13.850000	-0.736000	-0.157000
C	-14.560000	-1.618000	0.679000
C	-15.517000	-1.217000	1.688000
H	-15.799000	-0.195000	1.900000
C	-15.974000	-2.357000	2.277000
H	-16.701000	-2.450000	3.073000
C	-15.280000	-3.459000	1.655000
C	-15.375000	-4.792000	2.056000
C	-14.619000	-5.865000	1.581000
C	-14.648000	-7.200000	2.127000
H	-15.298000	-7.530000	2.927000
C	-13.708000	-7.924000	1.456000
C	-13.117000	-7.041000	0.473000
C	-12.138000	-7.420000	-0.465000
C	-11.686000	-6.594000	-1.516000
C	-10.725000	-6.976000	-2.531000
C	-10.554000	-5.898000	-3.346000
C	-11.369000	-4.837000	-2.811000
C	-11.346000	-3.520000	-3.270000
H	-9.908000	-5.812000	-4.210000
H	-10.243000	-7.939000	-2.604000
H	-13.446000	-8.963000	1.591000
C	-11.943000	-2.415000	-2.668000
N	-14.445000	-2.988000	0.671000
N	-12.066000	-5.286000	-1.716000
N	-13.692000	-5.795000	0.570000
H	-16.067000	5.008000	-2.864000
H	-10.730000	3.323000	4.142000
H	-16.068000	-5.007000	2.864000
H	-10.730000	-3.323000	-4.142000
C	-11.419000	-8.718000	-0.266000
C	-11.440000	-9.769000	-1.197000
H	-12.122000	-9.720000	-2.040000
C	-10.569000	-10.852000	-1.076000
H	-10.600000	-11.640000	-1.823000
C	-9.638000	-10.925000	-0.025000
C	-9.691000	-9.919000	0.956000
H	-8.981000	-9.932000	1.776000
C	-8.529000	-11.911000	-0.002000
C	-7.921000	-12.333000	-1.197000

C -6.719000 -13.033000 -1.188000
C -6.059000 -13.333000 0.016000
C -4.649000 -13.797000 0.039000
C -3.754000 -13.342000 -0.946000
C -2.386000 -13.558000 -0.833000
H -1.714000 -13.138000 -1.575000
C -1.846000 -14.228000 0.276000
C -2.743000 -14.770000 1.210000
H -2.357000 -15.332000 2.055000
H -4.124000 -12.738000 -1.768000
C -4.117000 -14.561000 1.092000
H -4.782000 -14.980000 1.841000
C -6.727000 -13.015000 1.212000
H -6.258000 -13.241000 2.164000
C -7.936000 -12.327000 1.203000
H -8.402000 -12.068000 2.148000
C -10.565000 -8.845000 0.841000
H -10.537000 -8.050000 1.580000
H -6.263000 -13.309000 -2.133000
H -8.354000 -12.045000 -2.150000

8

C -15.525000 11.438000 0.606000
C -15.481000 10.560000 -0.490000
C -16.140000 9.335000 -0.458000
H -16.039000 8.650000 -1.295000
C -16.867000 8.932000 0.673000
C -16.982000 9.842000 1.735000
H -17.574000 9.573000 2.605000
H -14.862000 10.805000 -1.347000
C -16.325000 11.071000 1.702000
H -16.425000 11.750000 2.543000
C -17.384000 7.530000 0.750000
C -18.364000 7.122000 -0.175000
N -18.811000 5.833000 -0.353000
C -19.780000 5.882000 -1.326000
C -20.455000 4.778000 -1.849000
C -20.249000 3.438000 -1.524000
C -20.893000 2.322000 -2.175000
H -21.643000 2.404000 -2.950000
C -20.361000 1.189000 -1.637000
H -20.592000 0.162000 -1.881000
C -19.403000 1.607000 -0.635000
C -18.636000 0.736000 0.159000
C -17.787000 1.156000 1.200000
C -17.047000 0.268000 2.075000
H -17.058000 -0.810000 2.021000
C -16.367000 1.054000 2.955000
C -16.660000 2.422000 2.608000
C -16.083000 3.534000 3.216000
C -16.223000 4.867000 2.832000
N -17.026000 5.323000 1.815000
C -15.465000 5.958000 3.389000
C -15.790000 7.071000 2.674000
H -15.392000 8.067000 2.790000
H -14.755000 5.871000 4.201000
H -15.711000 0.743000 3.758000
N -17.532000 2.462000 1.547000
N -19.359000 2.981000 -0.581000
C -19.966000 7.243000 -1.764000
H -20.677000 7.562000 -2.515000
Zn -18.198000 4.152000 0.622000
C -19.085000 8.010000 -1.062000
H -18.942000 9.079000 -1.120000
C -16.782000 6.673000 1.696000
H -21.190000 4.982000 -2.622000
H -15.403000 3.337000 4.040000
C -11.438000 -15.525000 -0.606000
C -11.071000 -16.325000 -1.702000
C -9.842000 -16.982000 -1.735000
C -8.932000 -16.867000 -0.673000
C -9.335000 -16.140000 0.458000
H -8.650000 -16.039000 1.295000
C -10.560000 -15.481000 0.490000
H -11.750000 -16.425000 -2.543000
H -10.805000 -14.862000 1.347000
H -14.862000 -10.805000 1.347000
C -13.007000 -13.990000 -1.817000
C -13.990000 -13.007000 -1.817000
H -16.039000 -8.650000 1.295000
C -15.481000 -10.560000 0.490000
C -12.635000 -14.649000 -0.632000
C -14.649000 -12.635000 -0.632000
C -16.140000 -9.335000 0.458000
C -15.525000 -11.438000 -0.606000
C -13.356000 -14.339000 0.534000
C -14.339000 -13.356000 0.534000
C -16.867000 -8.932000 -0.673000
C -16.325000 -11.071000 -1.702000
C -16.982000 -9.842000 -1.735000
H -16.425000 -11.750000 -2.543000
H -17.574000 -9.573000 -2.605000
H -9.573000 -17.574000 -2.605000
H -13.135000 -14.869000 1.456000
H -14.869000 -13.135000 1.456000
N -5.833000 -18.811000 0.353000
C -5.882000 -19.780000 1.326000
C -4.778000 -20.455000 1.849000
C -3.438000 -20.249000 1.524000
C -2.322000 -20.893000 2.175000
H -2.404000 -21.643000 2.950000
C -1.189000 -20.361000 1.637000
H -0.162000 -20.592000 1.881000
C -1.607000 -19.403000 0.635000
C -0.736000 -18.636000 -0.159000
C -1.156000 -17.787000 -1.200000
C -0.268000 -17.047000 -2.075000
H 0.810000 -17.058000 -2.021000
C -1.054000 -16.367000 -2.955000
H -0.743000 -15.711000 -3.758000
C -2.422000 -16.660000 -2.608000
C -3.534000 -16.083000 -3.216000
C -4.867000 -16.223000 -2.832000
N -5.323000 -17.026000 -1.815000
C -6.673000 -16.782000 -1.696000
C -7.530000 -17.384000 -0.750000
C -7.071000 -15.790000 -2.674000
H -8.067000 -15.392000 -2.790000
C -5.958000 -15.465000 -3.389000
H -5.871000 -14.755000 -4.201000
N -2.462000 -17.532000 -1.547000
N -2.981000 -19.359000 0.581000
C -7.243000 -19.966000 1.764000
H -7.562000 -20.677000 2.515000
C -8.010000 -19.085000 1.062000
H -9.079000 -18.942000 1.120000
Zn -4.152000 -18.198000 -0.622000
C -7.122000 -18.364000 0.175000
N -18.811000 -5.833000 0.353000
C -19.780000 -5.882000 1.326000
C -20.455000 -4.778000 1.849000
C -20.249000 -3.438000 1.524000
C -20.893000 -2.322000 2.174000
H -21.643000 -2.404000 2.950000
C -20.361000 -1.189000 1.637000
H -20.592000 -0.162000 1.880000
C -19.403000 -1.607000 0.635000
C -18.636000 -0.736000 -0.159000

C	-17.787000	-1.156000	-1.200000
C	-17.047000	-0.268000	-2.075000
H	-17.058000	0.810000	-2.021000
C	-16.367000	-1.054000	-2.955000
H	-15.711000	-0.743000	-3.758000
C	-16.660000	-2.422000	-2.608000
C	-16.083000	-3.534000	-3.216000
C	-16.223000	-4.867000	-2.832000
N	-17.026000	-5.323000	-1.815000
C	-16.782000	-6.673000	-1.696000
C	-17.384000	-7.530000	-0.750000
C	-15.790000	-7.071000	-2.674000
H	-15.392000	-8.067000	-2.791000
C	-15.465000	-5.958000	-3.389000
H	-14.755000	-5.871000	-4.201000
N	-17.532000	-2.462000	-1.547000
N	-19.359000	-2.981000	0.581000
C	-19.967000	-7.243000	1.764000
H	-20.677000	-7.562000	2.515000
C	-19.085000	-8.010000	1.062000
H	-18.942000	-9.079000	1.120000
Zn	-18.198000	-4.152000	-0.623000
C	-18.364000	-7.122000	0.175000
H	-14.192000	-12.462000	-2.734000
H	-12.462000	-14.192000	-2.734000
H	-4.982000	-21.190000	2.622000
H	-3.337000	-15.403000	-4.040000
H	-21.190000	-4.982000	2.622000
H	-15.403000	-3.337000	-4.040000
Zn	4.152000	-18.198000	0.623000
N	2.981000	-19.359000	-0.581000
C	1.607000	-19.403000	-0.635000
C	1.189000	-20.361000	-1.637000
H	0.162000	-20.592000	-1.880000
C	2.322000	-20.893000	-2.174000
H	2.404000	-21.643000	-2.950000
C	0.736000	-18.636000	0.159000
C	1.156000	-17.787000	1.200000
C	0.268000	-17.047000	2.075000
H	-0.810000	-17.058000	2.021000
C	1.054000	-16.367000	2.955000
H	0.743000	-15.711000	3.758000
C	2.422000	-16.660000	2.608000
C	3.534000	-16.083000	3.216000
C	4.867000	-16.223000	2.832000
C	5.958000	-15.465000	3.389000
C	7.071000	-15.790000	2.674000
C	6.673000	-16.782000	1.696000
C	7.530000	-17.384000	0.750000
C	7.122000	-18.364000	-0.175000
C	8.010000	-19.085000	-1.062000
C	7.243000	-19.967000	-1.764000
C	5.882000	-19.780000	-1.326000
C	4.778000	-20.455000	-1.849000
H	7.562000	-20.677000	-2.515000
H	9.079000	-18.942000	-1.120000
C	8.932000	-16.867000	0.673000
C	9.842000	-16.982000	1.735000
H	9.573000	-17.574000	2.605000
C	11.071000	-16.325000	1.702000
H	11.750000	-16.425000	2.543000
C	11.438000	-15.525000	0.606000
C	10.560000	-15.481000	-0.490000
H	10.805000	-14.862000	-1.347000
C	9.335000	-16.140000	-0.458000
H	8.650000	-16.039000	-1.295000
H	8.067000	-15.392000	2.791000
C	3.438000	-20.249000	-1.524000
N	2.462000	-17.532000	1.547000
N	5.833000	-18.811000	-0.353000
N	5.323000	-17.026000	1.815000
H	3.337000	-15.403000	4.040000
H	4.982000	-21.190000	-2.622000
H	5.871000	-14.755000	4.201000
C	12.635000	-14.649000	0.632000
C	13.356000	-14.339000	-0.534000
C	14.339000	-13.356000	-0.534000
C	14.649000	-12.635000	0.632000
C	13.990000	-13.007000	1.817000
H	14.192000	-12.462000	2.734000
C	13.007000	-13.990000	1.817000
H	12.462000	-14.192000	2.734000
H	14.869000	-13.135000	-1.456000
H	13.135000	-14.869000	-1.456000
C	-12.635000	14.649000	0.632000
C	-13.356000	14.339000	-0.534000
H	-13.135000	14.869000	-1.456000
C	-14.339000	13.356000	-0.534000
H	-14.869000	13.135000	-1.456000
C	-14.649000	12.635000	0.632000
C	-13.990000	13.007000	1.817000
C	-13.007000	13.990000	1.817000
H	-14.192000	12.462000	2.734000
H	-12.462000	14.192000	2.734000
C	15.525000	-11.438000	0.606000
C	15.481000	-10.560000	-0.490000
C	16.140000	-9.335000	-0.458000
H	16.039000	-8.650000	-1.295000
C	16.867000	-8.932000	0.673000
C	16.982000	-9.842000	1.735000
H	17.574000	-9.573000	2.605000
H	14.862000	-10.805000	-1.347000
C	16.325000	-11.071000	1.702000
H	16.425000	-11.750000	2.543000
C	17.384000	-7.530000	0.750000
C	18.364000	-7.122000	-0.175000
N	18.811000	-5.833000	-0.353000
C	19.780000	-5.882000	-1.326000
C	20.455000	-4.778000	-1.849000
C	20.249000	-3.438000	-1.524000
C	20.893000	-2.322000	-2.175000
H	21.643000	-2.404000	-2.950000
C	20.361000	-1.189000	-1.637000
H	20.592000	-0.162000	-1.881000
C	19.403000	-1.607000	-0.635000
C	18.636000	-0.736000	0.159000
C	17.787000	-1.156000	1.200000
C	17.047000	-0.268000	2.075000
H	17.058000	0.810000	2.021000
C	16.367000	-1.054000	2.955000
C	16.660000	-2.422000	2.608000
C	16.083000	-3.534000	3.216000
C	16.223000	-4.867000	2.832000
N	17.026000	-5.323000	1.815000
C	15.465000	-5.958000	3.389000
C	15.790000	-7.071000	2.674000
H	15.392000	-8.067000	2.790000
H	14.755000	-5.871000	4.201000
H	15.711000	-0.743000	3.758000
N	17.532000	-2.462000	1.547000
N	19.359000	-2.981000	-0.581000
C	19.966000	-7.243000	-1.764000
H	20.677000	-7.562000	-2.515000
Zn	18.198000	-4.152000	0.622000
C	19.085000	-8.010000	-1.062000
H	18.942000	-9.079000	-1.120000
C	16.782000	-6.673000	1.696000
H	21.190000	-4.982000	-2.622000
H	15.403000	-3.337000	4.040000
C	11.438000	15.525000	-0.606000
C	11.071000	16.325000	-1.702000
C	9.842000	16.982000	-1.735000

C	8.932000	16.867000	-0.673000
C	9.335000	16.140000	0.458000
H	8.650000	16.039000	1.295000
C	10.560000	15.481000	0.490000
H	11.750000	16.425000	-2.543000
H	10.805000	14.862000	1.347000
H	14.862000	10.805000	1.347000
C	13.007000	13.990000	-1.817000
C	13.990000	13.007000	-1.817000
H	16.039000	8.650000	1.295000
C	15.481000	10.560000	0.490000
C	12.635000	14.649000	-0.632000
C	14.649000	12.635000	-0.632000
C	16.140000	9.335000	0.458000
C	15.525000	11.438000	-0.606000
C	13.356000	14.339000	0.534000
C	14.339000	13.356000	0.534000
C	16.867000	8.932000	-0.673000
C	16.325000	11.071000	-1.702000
C	16.982000	9.842000	-1.735000
H	16.425000	11.750000	-2.543000
H	17.574000	9.573000	-2.605000
H	9.573000	17.574000	-2.605000
H	13.135000	14.869000	1.456000
H	14.869000	13.135000	1.456000
N	5.833000	18.811000	0.353000
C	5.882000	19.780000	1.326000
C	4.778000	20.455000	1.849000
C	3.438000	20.249000	1.524000
C	2.322000	20.893000	2.175000
H	2.404000	21.643000	2.950000
C	1.189000	20.361000	1.637000
H	0.162000	20.592000	1.881000
C	1.607000	19.403000	0.635000
C	0.736000	18.636000	-0.159000
C	1.156000	17.787000	-1.200000
C	0.268000	17.047000	-2.075000
H	-0.810000	17.058000	-2.021000
C	1.054000	16.367000	-2.955000
H	0.743000	15.711000	-3.758000
C	2.422000	16.660000	-2.608000
C	3.534000	16.083000	-3.216000
C	4.867000	16.223000	-2.832000
N	5.323000	17.026000	-1.815000
C	6.673000	16.782000	-1.696000
C	7.530000	17.384000	-0.750000
C	7.071000	15.790000	-2.674000
H	8.067000	15.392000	-2.790000
C	5.958000	15.465000	-3.389000
H	5.871000	14.755000	-4.201000
N	2.462000	17.532000	-1.547000
N	2.981000	19.359000	0.581000
C	7.243000	19.966000	1.764000
H	7.562000	20.677000	2.515000
C	8.010000	19.085000	1.062000
H	9.079000	18.942000	1.120000
Zn	4.152000	18.198000	-0.622000
C	7.122000	18.364000	0.175000
N	18.811000	5.833000	0.353000
C	19.780000	5.882000	1.326000
C	20.455000	4.778000	1.849000
C	20.249000	3.438000	1.524000
C	20.893000	2.322000	2.174000
H	21.643000	2.404000	2.950000
C	20.361000	1.189000	1.637000
H	20.592000	0.162000	1.880000
C	19.403000	1.607000	0.635000
C	18.636000	0.736000	-0.159000
C	17.787000	1.156000	-1.200000
C	17.047000	0.268000	-2.075000
H	17.058000	-0.810000	-2.021000

C	16.367000	1.054000	-2.955000
H	15.711000	0.743000	-3.758000
C	16.660000	2.422000	-2.608000
C	16.083000	3.534000	-3.216000
C	16.223000	4.867000	-2.832000
N	17.026000	5.323000	-1.815000
C	16.782000	6.673000	-1.696000
C	17.384000	7.530000	-0.750000
C	15.790000	7.071000	-2.674000
H	15.392000	8.067000	-2.791000
C	15.465000	5.958000	-3.389000
H	14.755000	5.871000	-4.201000
N	17.532000	2.462000	-1.547000
N	19.359000	2.981000	0.581000
C	19.967000	7.243000	1.764000
H	20.677000	7.562000	2.515000
C	19.085000	8.010000	1.062000
H	18.942000	9.079000	1.120000
Zn	18.198000	4.152000	-0.623000
C	18.364000	7.122000	0.175000
H	14.192000	12.462000	-2.734000
H	12.462000	14.192000	-2.734000
H	4.982000	21.190000	2.622000
H	3.337000	15.403000	-4.040000
H	21.190000	4.982000	2.622000
H	15.403000	3.337000	-4.040000
Zn	-4.152000	18.198000	0.623000
N	-2.981000	19.359000	-0.581000
C	-1.607000	19.403000	-0.635000
C	-1.189000	20.361000	-1.637000
H	-0.162000	20.592000	-1.880000
C	-2.322000	20.893000	-2.174000
H	-2.404000	21.643000	-2.950000
C	-0.736000	18.636000	0.159000
C	-1.156000	17.787000	1.200000
C	-0.268000	17.047000	2.075000
H	0.810000	17.058000	2.021000
C	-1.054000	16.367000	2.955000
H	-0.743000	15.711000	3.758000
C	-2.422000	16.660000	2.608000
C	-3.534000	16.083000	3.216000
C	-4.867000	16.223000	2.832000
C	-5.958000	15.465000	3.389000
C	-7.071000	15.790000	2.674000
C	-6.673000	16.782000	1.696000
C	-7.530000	17.384000	0.750000
C	-7.122000	18.364000	-0.175000
C	-8.010000	19.085000	-1.062000
C	-7.243000	19.967000	-1.764000
C	-5.882000	19.780000	-1.326000
C	-4.778000	20.455000	-1.849000
H	-7.562000	20.677000	-2.515000
H	-9.079000	18.942000	-1.120000
C	-8.932000	16.867000	0.673000
C	-9.842000	16.982000	1.735000
H	-9.573000	17.574000	2.605000
C	-11.071000	16.325000	1.702000
H	-11.750000	16.425000	2.543000
C	-11.438000	15.525000	0.606000
C	-10.560000	15.481000	-0.490000
H	-10.805000	14.862000	-1.347000
C	-9.335000	16.140000	-0.458000
H	-8.650000	16.039000	-1.295000
H	-8.067000	15.392000	2.791000
C	-3.438000	20.249000	-1.524000
N	-2.462000	17.532000	1.547000
N	-5.833000	18.811000	-0.353000
N	-5.323000	17.026000	1.815000
H	-3.337000	15.403000	4.040000
H	-4.982000	21.190000	-2.622000
H	-5.871000	14.755000	4.201000

9

C	14.210000	2.876000	-0.701000
C	13.163000	3.376000	-1.493000
C	12.696000	4.674000	-1.335000
H	11.842000	5.010000	-1.914000
C	13.253000	5.538000	-0.375000
C	14.360000	5.071000	0.353000
H	14.847000	5.731000	1.064000
H	12.653000	2.713000	-2.185000
C	14.826000	3.765000	0.197000
H	15.674000	3.429000	0.787000
C	14.515000	-1.420000	-0.747000
C	14.416000	-0.693000	-1.946000
C	14.416000	0.697000	-1.946000
C	14.515000	1.425000	-0.747000
C	14.739000	0.699000	0.435000
H	14.816000	1.228000	1.380000
C	14.739000	-0.694000	0.435000
H	14.816000	-1.223000	1.380000
H	14.311000	1.229000	-2.887000
H	14.311000	-1.225000	-2.887000
C	13.255000	-5.533000	-0.374000
C	12.698000	-4.671000	-1.334000
H	11.843000	-5.006000	-1.913000
C	13.164000	-3.372000	-1.492000
H	12.654000	-2.709000	-2.184000
C	14.211000	-2.872000	-0.700000
C	14.827000	-3.760000	0.197000
H	15.675000	-3.424000	0.788000
C	14.362000	-5.066000	0.354000
H	14.849000	-5.726000	1.066000
Zn	10.275000	-9.408000	0.240000
N	9.379000	-10.715000	-1.045000
C	8.224000	-11.410000	-0.836000
C	7.854000	-12.153000	-2.024000
C	8.842000	-11.911000	-2.949000
C	7.458000	-11.434000	0.345000
C	7.850000	-10.759000	1.517000
C	7.091000	-10.830000	2.749000
C	7.777000	-10.071000	3.667000
H	7.518000	-9.872000	4.698000
C	8.914000	-9.515000	2.978000
C	9.792000	-8.573000	3.508000
C	10.770000	-7.854000	2.813000
C	11.465000	-6.711000	3.346000
H	11.374000	-6.345000	4.360000
C	12.187000	-6.174000	2.322000
C	11.984000	-7.021000	1.166000
C	12.559000	-6.830000	-0.111000
C	12.386000	-7.713000	-1.200000
C	13.107000	-7.638000	-2.451000
C	12.665000	-8.667000	-3.231000
C	11.654000	-9.360000	-2.475000
C	10.859000	-10.399000	-2.972000
H	12.981000	-8.927000	-4.232000
H	13.864000	-6.904000	-2.689000
H	12.793000	-5.282000	2.347000
C	9.772000	-10.996000	-2.335000
N	8.944000	-9.959000	1.674000
N	11.521000	-8.781000	-1.235000
N	11.114000	-8.038000	1.496000
H	9.644000	-8.300000	4.549000
H	11.068000	-10.723000	-3.987000
C	-0.357000	-14.285000	-0.106000
C	0.494000	-14.578000	-1.196000
N	1.851000	-14.363000	-1.232000
C	2.286000	-14.768000	-2.471000
C	3.583000	-14.600000	-2.969000
C	4.644000	-13.957000	-2.334000
C	5.900000	-13.609000	-2.948000
H	6.185000	-13.858000	-3.962000
C	6.605000	-12.875000	-2.024000
C	5.777000	-12.823000	-0.836000
C	6.182000	-12.172000	0.346000
C	5.402000	-12.173000	1.518000
C	5.843000	-11.551000	2.749000
C	4.843000	-11.766000	3.668000
C	3.793000	-12.473000	2.980000
C	2.538000	-12.762000	3.511000
C	1.426000	-13.249000	2.816000
N	1.413000	-13.639000	1.499000
C	0.090000	-13.279000	3.350000
C	-0.737000	-13.636000	2.327000
H	-1.813000	-13.715000	2.353000
H	-0.181000	-13.018000	4.364000
H	4.802000	-11.441000	4.699000
N	4.162000	-12.721000	1.676000
N	4.597000	-13.476000	-1.043000
C	1.179000	-15.296000	-3.227000
H	1.246000	-15.700000	-4.229000
Zn	3.018000	-13.598000	0.243000
C	0.067000	-15.165000	-2.447000
H	-0.946000	-15.453000	-2.684000
C	0.097000	-13.884000	1.170000
H	3.758000	-14.943000	-3.985000
H	2.377000	-12.497000	4.552000
C	-4.611000	-13.737000	-0.694000
C	-3.654000	-13.080000	-1.486000
C	-2.297000	-13.325000	-1.328000
H	-1.579000	-12.753000	-1.907000
C	-1.828000	-14.240000	-0.369000
C	-2.785000	-14.965000	0.360000
H	-2.457000	-15.717000	1.070000
H	-3.974000	-12.307000	-2.178000
C	-4.149000	-14.716000	0.203000
H	-4.864000	-15.282000	0.793000
C	-8.484000	-11.854000	-0.742000
C	-7.805000	-12.132000	-1.941000
C	-6.601000	-12.827000	-1.940000
C	-6.021000	-13.275000	-0.741000
C	-6.761000	-13.106000	0.441000
H	-6.342000	-13.438000	1.386000
C	-7.968000	-12.410000	0.440000
H	-8.465000	-12.213000	1.385000
H	-6.087000	-13.001000	-2.881000
H	-8.213000	-11.775000	-2.882000
C	-11.419000	-8.708000	-0.373000
C	-10.392000	-8.656000	-1.331000
H	-10.255000	-7.748000	-1.909000
C	-9.499000	-9.708000	-1.488000
H	-8.670000	-9.598000	-2.178000
C	-9.590000	-10.865000	-0.696000
C	-10.669000	-10.955000	0.200000
H	-10.801000	-11.858000	0.790000
C	-11.568000	-9.900000	0.356000
H	-12.384000	-9.993000	1.066000
Zn	-13.291000	-4.192000	0.236000
N	-13.973000	-2.763000	-1.050000
C	-13.998000	-1.416000	-0.843000
C	-14.454000	-0.724000	-2.032000
C	-14.735000	-1.701000	-2.957000
C	-13.639000	-0.739000	0.340000
C	-13.253000	-1.416000	1.513000
C	-12.939000	-0.722000	2.745000
C	-12.626000	-1.696000	3.664000
H	-12.327000	-1.570000	4.696000
C	-12.711000	-2.959000	2.976000

C	-12.334000	-4.190000	3.507000
C	-12.198000	-5.396000	2.812000
C	-11.555000	-6.568000	3.347000
H	-11.195000	-6.672000	4.362000
C	-11.448000	-7.462000	2.324000
C	-12.078000	-6.864000	1.166000
C	-12.196000	-7.457000	-0.111000
C	-12.873000	-6.867000	-1.202000
C	-13.165000	-7.530000	-2.454000
C	-13.833000	-6.633000	-3.235000
C	-13.931000	-5.411000	-2.479000
C	-14.433000	-4.204000	-2.978000
H	-14.214000	-6.777000	-4.237000
H	-12.906000	-8.552000	-2.690000
H	-10.977000	-8.433000	2.350000
C	-14.409000	-2.964000	-2.342000
N	-13.108000	-2.763000	1.670000
N	-13.366000	-5.584000	-1.239000
N	-12.526000	-5.603000	1.494000
H	-12.027000	-4.197000	4.548000
H	-14.816000	-4.224000	-3.994000
C	-12.198000	7.453000	-0.113000
C	-12.875000	6.862000	-1.203000
N	-13.368000	5.580000	-1.240000
C	-13.933000	5.406000	-2.480000
C	-14.435000	4.198000	-2.979000
C	-14.410000	2.958000	-2.343000
C	-14.735000	1.696000	-2.958000
H	-15.091000	1.573000	-3.972000
C	-14.454000	0.719000	-2.032000
C	-13.998000	1.411000	-0.843000
C	-13.639000	0.735000	0.339000
C	-13.254000	1.412000	1.512000
C	-12.939000	0.719000	2.745000
C	-12.627000	1.692000	3.664000
C	-12.712000	2.955000	2.975000
C	-12.336000	4.186000	3.506000
C	-12.199000	5.392000	2.811000
N	-12.527000	5.599000	1.493000
C	-11.557000	6.565000	3.345000
C	-11.450000	7.459000	2.322000
H	-10.980000	8.430000	2.348000
H	-11.197000	6.669000	4.360000
H	-12.327000	1.567000	4.696000
N	-13.109000	2.759000	1.670000
N	-13.973000	2.758000	-1.051000
C	-13.835000	6.628000	-3.236000
H	-14.217000	6.772000	-4.239000
Zn	-13.292000	4.187000	0.236000
C	-13.167000	7.525000	-2.455000
H	-12.909000	8.547000	-2.692000
C	-12.080000	6.860000	1.165000
H	-14.817000	4.218000	-3.995000
H	-12.028000	4.194000	4.548000
C	-9.594000	10.862000	-0.698000
C	-9.502000	9.704000	-1.489000
C	-10.395000	8.652000	-1.332000
H	-10.258000	7.744000	-1.911000
C	-11.422000	8.704000	-0.374000
C	-11.571000	9.896000	0.354000
H	-12.387000	9.989000	1.064000
H	-8.673000	9.594000	-2.180000
C	-10.672000	10.952000	0.198000
H	-10.805000	11.854000	0.788000
C	-6.025000	13.273000	-0.743000
C	-6.605000	12.824000	-1.943000
C	-7.809000	12.129000	-1.943000
C	-8.488000	11.851000	-0.744000
C	-7.972000	12.408000	0.438000
H	-8.469000	12.210000	1.383000
C	-6.766000	13.104000	0.438000
H	-6.346000	13.436000	1.383000
H	-8.217000	11.772000	-2.884000
H	-6.091000	12.999000	-2.883000
C	-1.832000	14.239000	-0.372000
C	-2.301000	13.324000	-1.331000
H	-1.583000	12.752000	-1.909000
C	-3.659000	13.078000	-1.488000
H	-3.978000	12.305000	-2.180000
C	-4.616000	13.736000	-0.697000
C	-4.154000	14.714000	0.200000
H	-4.869000	15.280000	0.790000
C	-2.790000	14.964000	0.357000
H	-2.463000	15.717000	1.067000
Zn	3.014000	13.599000	0.240000
N	4.593000	13.477000	-1.046000
C	5.772000	12.825000	-0.838000
C	6.601000	12.876000	-2.026000
C	5.896000	13.610000	-2.951000
C	6.178000	12.174000	0.343000
C	5.397000	12.175000	1.515000
C	5.840000	11.554000	2.747000
C	4.840000	11.768000	3.665000
H	4.798000	11.443000	4.697000
C	3.789000	12.475000	2.978000
C	2.534000	12.763000	3.508000
C	1.422000	13.250000	2.814000
C	0.086000	13.280000	3.347000
H	-0.185000	13.019000	4.362000
C	-0.742000	13.637000	2.325000
C	0.093000	13.884000	1.168000
C	-0.361000	14.285000	-0.109000
C	0.489000	14.578000	-1.199000
C	0.062000	15.164000	-2.450000
C	1.174000	15.296000	-3.230000
C	2.281000	14.768000	-2.474000
C	3.578000	14.600000	-2.972000
H	1.241000	15.700000	-4.232000
H	-0.952000	15.452000	-2.687000
H	-1.817000	13.715000	2.350000
C	4.639000	13.958000	-2.336000
N	4.158000	12.723000	1.673000
N	1.847000	14.363000	-1.235000
N	1.408000	13.640000	1.496000
H	2.373000	12.499000	4.549000
H	3.753000	14.943000	-3.988000
C	12.556000	6.834000	-0.112000
C	12.383000	7.717000	-1.202000
N	11.518000	8.785000	-1.237000
C	11.651000	9.364000	-2.476000
C	10.856000	10.402000	-2.974000
C	9.769000	10.999000	-2.338000
C	8.838000	11.913000	-2.952000
H	8.910000	12.285000	-3.965000
C	7.850000	12.156000	-2.026000
C	8.221000	11.412000	-0.839000
C	7.454000	11.437000	0.343000
C	7.847000	10.762000	1.515000
C	7.088000	10.833000	2.747000
C	7.774000	10.075000	3.665000
C	8.911000	9.519000	2.977000
C	9.789000	8.577000	3.506000
C	10.767000	7.858000	2.811000
N	11.112000	8.042000	1.494000
C	11.462000	6.716000	3.344000
C	12.185000	6.178000	2.321000
H	12.791000	5.287000	2.346000
H	11.372000	6.350000	4.359000
H	7.514000	9.876000	4.696000
N	8.941000	9.963000	1.672000
N	9.376000	10.718000	-1.047000
C	12.662000	8.670000	-3.232000

H	12.978000	8.931000	-4.234000
Zn	10.272000	9.412000	0.238000
C	13.105000	7.642000	-2.453000
H	13.861000	6.908000	-2.690000
C	11.981000	7.026000	1.165000
H	11.065000	10.726000	-3.989000
H	9.641000	8.304000	4.547000
H	8.915000	-12.283000	-3.963000
H	-15.091000	-1.578000	-3.972000
H	6.181000	13.859000	-3.965000

11

C	15.152000	-11.745000	-0.536000
C	14.864000	-10.624000	-1.332000
C	15.593000	-9.447000	-1.212000
H	15.315000	-8.581000	-1.802000
C	16.644000	-9.335000	-0.285000
C	16.977000	-10.474000	0.465000
H	17.811000	-10.430000	1.159000
H	14.014000	-10.652000	-2.007000
C	16.244000	-11.655000	0.344000
H	16.524000	-12.518000	0.940000
C	12.183000	-14.880000	-0.574000
C	12.617000	-14.279000	-1.769000
C	13.624000	-13.322000	-1.768000
C	14.246000	-12.919000	-0.574000
C	13.878000	-13.587000	0.606000
H	14.330000	-13.294000	1.549000
C	12.869000	-14.546000	0.606000
H	12.553000	-14.983000	1.549000
H	13.928000	-12.869000	-2.708000
H	12.149000	-14.559000	-2.708000
C	8.481000	-17.091000	-0.287000
C	8.647000	-16.048000	-1.212000
H	7.795000	-15.725000	-1.803000
C	9.859000	-15.379000	-1.333000
H	9.930000	-14.531000	-2.007000
C	10.964000	-15.724000	-0.536000
C	10.818000	-16.810000	0.343000
H	11.665000	-17.134000	0.939000
C	9.601000	-17.482000	0.464000
H	9.514000	-18.313000	1.157000
Zn	18.408000	3.739000	0.182000
N	18.999000	2.283000	-1.119000
C	18.969000	0.935000	-0.917000
C	19.398000	0.230000	-2.108000
C	19.713000	1.198000	-3.032000
C	18.584000	0.267000	0.262000
C	18.213000	0.954000	1.434000
C	17.861000	0.268000	2.661000
C	17.576000	1.249000	3.581000
H	17.265000	1.130000	4.610000
C	17.725000	2.510000	2.902000
C	17.425000	3.756000	3.447000
C	17.400000	4.983000	2.776000
C	16.882000	6.203000	3.337000
H	16.524000	6.319000	4.351000
C	16.893000	7.133000	2.341000
C	17.463000	6.498000	1.172000
C	17.659000	7.112000	-0.085000
C	18.250000	6.477000	-1.199000
C	18.598000	7.133000	-2.440000
C	19.157000	6.187000	-3.249000
C	19.140000	4.947000	-2.517000
C	19.541000	3.711000	-3.036000
H	19.541000	6.312000	-4.253000
H	18.447000	8.181000	-2.652000
H	16.539000	8.152000	2.389000
C	19.443000	2.470000	-2.411000
N	18.123000	2.306000	1.598000
N	18.603000	5.149000	-1.268000
N	17.762000	5.186000	1.467000
H	17.108000	3.770000	4.486000
H	19.919000	3.716000	-4.054000
C	17.280000	-7.997000	-0.084000
C	17.903000	-7.392000	-1.198000
N	18.322000	-6.084000	-1.267000
C	18.868000	-5.909000	-2.516000
C	19.331000	-4.695000	-3.035000
C	19.295000	-3.451000	-2.410000
C	19.628000	-2.194000	-3.032000
H	19.986000	-2.079000	-4.046000
C	19.362000	-1.211000	-2.108000
C	18.899000	-1.893000	-0.916000
C	18.547000	-1.207000	0.262000
C	18.143000	-1.875000	1.435000
C	17.825000	-1.172000	2.661000
C	17.492000	-2.137000	3.581000
C	17.577000	-3.404000	2.903000
C	17.216000	-4.634000	3.448000
C	17.130000	-5.858000	2.777000
N	17.481000	-6.079000	1.468000
C	16.551000	-7.050000	3.338000
C	16.514000	-7.980000	2.342000
H	16.110000	-8.979000	2.390000
H	16.187000	-7.147000	4.352000
H	17.187000	-2.003000	4.611000
N	17.985000	-3.220000	1.599000
N	18.861000	-3.242000	-1.118000
C	18.824000	-7.149000	-3.248000
H	19.201000	-7.293000	-4.252000
Zn	18.198000	-4.666000	0.183000
C	18.217000	-8.065000	-2.439000
H	18.014000	-9.104000	-2.651000
C	17.116000	-7.374000	1.173000
H	19.709000	-4.719000	-4.053000
H	16.898000	-4.631000	4.487000
H	20.064000	1.065000	-4.046000
C	11.745000	15.152000	-0.535000
C	10.624000	14.864000	-1.332000
C	9.447000	15.593000	-1.211000
H	8.581000	15.315000	-1.802000
C	9.335000	16.644000	-0.285000
C	10.474000	16.977000	0.466000
H	10.430000	17.811000	1.159000
H	10.652000	14.014000	-2.006000
C	11.655000	16.244000	0.344000
H	12.518000	16.524000	0.941000
C	14.880000	12.183000	-0.573000
C	14.279000	12.617000	-1.768000
C	13.322000	13.624000	-1.768000
C	12.919000	14.246000	-0.573000
C	13.587000	13.878000	0.607000
H	13.294000	14.330000	1.549000
C	14.546000	12.869000	0.607000
H	14.983000	12.553000	1.549000
H	12.869000	13.928000	-2.707000
H	14.559000	12.149000	-2.707000
C	17.091000	8.481000	-0.286000
C	16.048000	8.647000	-1.212000
H	15.725000	7.795000	-1.803000
C	15.379000	9.859000	-1.333000
H	14.532000	9.930000	-2.007000
C	15.724000	10.964000	-0.536000
C	16.810000	10.818000	0.343000
H	17.134000	11.665000	0.940000
C	17.482000	9.601000	0.465000
H	18.313000	9.514000	1.158000

Zn	-3.739000	18.408000	0.183000
N	-2.283000	18.999000	-1.118000
C	-0.935000	18.969000	-0.916000
C	-0.230000	19.398000	-2.107000
C	-1.198000	19.713000	-3.031000
C	-0.267000	18.583000	0.263000
C	-0.954000	18.213000	1.435000
C	-0.268000	17.861000	2.661000
C	-1.249000	17.576000	3.581000
H	-1.130000	17.265000	4.611000
C	-2.511000	17.724000	2.902000
C	-3.756000	17.424000	3.448000
C	-4.983000	17.400000	2.776000
C	-6.203000	16.882000	3.337000
H	-6.319000	16.524000	4.352000
C	-7.134000	16.892000	2.341000
C	-6.498000	17.463000	1.172000
C	-7.112000	17.659000	-0.085000
C	-6.477000	18.250000	-1.199000
C	-7.133000	18.598000	-2.440000
C	-6.187000	19.158000	-3.248000
C	-4.947000	19.140000	-2.517000
C	-3.711000	19.541000	-3.036000
H	-6.312000	19.542000	-4.252000
H	-8.181000	18.447000	-2.652000
H	-8.152000	16.539000	2.390000
C	-2.470000	19.443000	-2.410000
N	-2.306000	18.122000	1.598000
N	-5.149000	18.603000	-1.268000
N	-5.186000	17.762000	1.467000
H	-3.770000	17.108000	4.486000
H	-3.716000	19.920000	-4.054000
C	7.997000	17.280000	-0.084000
C	7.392000	17.903000	-1.197000
N	6.084000	18.322000	-1.267000
C	5.909000	18.868000	-2.516000
C	4.695000	19.331000	-3.035000
C	3.451000	19.295000	-2.409000
C	2.194000	19.628000	-3.031000
H	2.079000	19.986000	-4.046000
C	1.211000	19.362000	-2.107000
C	1.893000	18.899000	-0.916000
C	1.207000	18.547000	0.263000
C	1.875000	18.142000	1.435000
C	1.172000	17.825000	2.661000
C	2.137000	17.491000	3.582000
C	3.404000	17.577000	2.903000
C	4.634000	17.215000	3.449000
C	5.857000	17.130000	2.778000
N	6.079000	17.481000	1.468000
C	7.050000	16.550000	3.338000
C	7.980000	16.514000	2.342000
H	8.979000	16.110000	2.391000
H	7.147000	16.187000	4.353000
H	2.003000	17.187000	4.611000
N	3.220000	17.985000	1.599000
N	3.242000	18.861000	-1.118000
C	7.149000	18.824000	-3.247000
H	7.293000	19.201000	-4.251000
Zn	4.666000	18.198000	0.184000
C	8.065000	18.217000	-2.438000
H	9.105000	18.014000	-2.651000
C	7.374000	17.116000	1.173000
H	4.719000	19.709000	-4.053000
H	4.631000	16.898000	4.487000
H	-1.065000	20.065000	-4.046000
C	-15.152000	11.745000	-0.535000
C	-14.864000	10.624000	-1.332000
C	-15.593000	9.447000	-1.212000
H	-15.315000	8.581000	-1.802000
C	-16.644000	9.335000	-0.285000
C	-16.977000	10.474000	0.465000
H	-17.811000	10.430000	1.159000
H	-14.014000	10.652000	-2.007000
C	-16.244000	11.655000	0.344000
H	-16.524000	12.518000	0.940000
C	-12.183000	14.880000	-0.574000
C	-12.617000	14.279000	-1.768000
C	-13.624000	13.322000	-1.768000
C	-14.246000	12.919000	-0.573000
C	-13.878000	13.587000	0.606000
H	-14.330000	13.294000	1.549000
C	-12.869000	14.546000	0.606000
H	-12.553000	14.983000	1.549000
H	-13.928000	12.869000	-2.707000
H	-12.149000	14.559000	-2.708000
C	-8.481000	17.091000	-0.286000
C	-8.647000	16.048000	-1.212000
H	-7.795000	15.725000	-1.803000
C	-9.859000	15.379000	-1.333000
H	-9.930000	14.532000	-2.007000
C	-10.964000	15.724000	-0.536000
C	-10.818000	16.810000	0.343000
H	-11.665000	17.134000	0.940000
C	-9.601000	17.482000	0.465000
H	-9.514000	18.313000	1.158000
Zn	-18.408000	-3.739000	0.182000
N	-18.999000	-2.283000	-1.119000
C	-18.969000	-0.935000	-0.917000
C	-19.398000	-0.230000	-2.108000
C	-19.713000	-1.198000	-3.032000
C	-18.584000	-0.267000	0.262000
C	-18.213000	-0.954000	1.434000
C	-17.861000	-0.268000	2.661000
C	-17.576000	-1.249000	3.580000
H	-17.265000	-1.130000	4.610000
C	-17.725000	-2.511000	2.902000
C	-17.425000	-3.756000	3.447000
C	-17.400000	-4.983000	2.776000
C	-16.882000	-6.203000	3.336000
H	-16.524000	-6.319000	4.351000
C	-16.893000	-7.134000	2.340000
C	-17.463000	-6.498000	1.171000
C	-17.659000	-7.112000	-0.086000
C	-18.250000	-6.477000	-1.199000
C	-18.598000	-7.133000	-2.441000
C	-19.157000	-6.187000	-3.249000
C	-19.140000	-4.947000	-2.518000
C	-19.541000	-3.711000	-3.037000
H	-19.541000	-6.312000	-4.253000
H	-18.447000	-8.181000	-2.653000
H	-16.539000	-8.152000	2.389000
C	-19.443000	-2.470000	-2.411000
N	-18.123000	-2.306000	1.598000
N	-18.603000	-5.149000	-1.269000
N	-17.762000	-5.186000	1.466000
H	-17.108000	-3.770000	4.485000
H	-19.919000	-3.716000	-4.054000
C	-17.280000	7.997000	-0.084000
C	-17.903000	7.392000	-1.198000
N	-18.322000	6.084000	-1.267000
C	-18.868000	5.909000	-2.517000
C	-19.331000	4.695000	-3.036000
C	-19.295000	3.451000	-2.410000
C	-19.628000	2.194000	-3.032000
H	-19.986000	2.079000	-4.046000
C	-19.362000	1.211000	-2.108000
C	-18.898000	1.893000	-0.916000
C	-18.547000	1.207000	0.262000
C	-18.143000	1.875000	1.435000
C	-17.825000	1.172000	2.661000
C	-17.492000	2.137000	3.581000

C	-17.577000	3.404000	2.903000
C	-17.216000	4.634000	3.448000
C	-17.130000	5.857000	2.777000
N	-17.481000	6.079000	1.468000
C	-16.551000	7.050000	3.338000
C	-16.514000	7.980000	2.342000
H	-16.110000	8.979000	2.390000
H	-16.187000	7.147000	4.352000
H	-17.187000	2.003000	4.610000
N	-17.985000	3.220000	1.598000
N	-18.861000	3.242000	-1.118000
C	-18.824000	7.149000	-3.248000
H	-19.201000	7.293000	-4.252000
Zn	-18.198000	4.666000	0.183000
C	-18.217000	8.065000	-2.439000
H	-18.014000	9.105000	-2.651000
C	-17.116000	7.374000	1.172000
H	-19.709000	4.719000	-4.053000
H	-16.898000	4.631000	4.487000
H	-20.064000	-1.065000	-4.047000
C	-11.745000	-15.152000	-0.536000
C	-10.624000	-14.864000	-1.332000
C	-9.447000	-15.593000	-1.212000
H	-8.581000	-15.315000	-1.802000
C	-9.335000	-16.644000	-0.286000
C	-10.474000	-16.977000	0.465000
H	-10.430000	-17.811000	1.159000
H	-10.652000	-14.014000	-2.007000
C	-11.655000	-16.244000	0.344000
H	-12.518000	-16.524000	0.940000
C	-14.880000	-12.183000	-0.574000
C	-14.279000	-12.617000	-1.769000
C	-13.322000	-13.624000	-1.769000
C	-12.919000	-14.246000	-0.574000
C	-13.587000	-13.878000	0.606000
H	-13.294000	-14.330000	1.549000
C	-14.546000	-12.869000	0.606000
H	-14.983000	-12.553000	1.549000
H	-12.869000	-13.928000	-2.708000
H	-14.559000	-12.149000	-2.708000
C	-17.091000	-8.481000	-0.287000
C	-16.048000	-8.647000	-1.213000
H	-15.725000	-7.795000	-1.803000
C	-15.379000	-9.859000	-1.333000
H	-14.531000	-9.930000	-2.007000
C	-15.724000	-10.964000	-0.536000
C	-16.810000	-10.818000	0.343000
H	-17.134000	-11.665000	0.939000
C	-17.482000	-9.601000	0.464000
H	-18.313000	-9.514000	1.157000
Zn	3.739000	-18.408000	0.182000
N	2.283000	-18.999000	-1.119000
C	0.935000	-18.969000	-0.917000
C	0.230000	-19.398000	-2.108000
C	1.198000	-19.713000	-3.032000
C	0.267000	-18.583000	0.262000
C	0.954000	-18.213000	1.434000
C	0.268000	-17.861000	2.661000
C	1.249000	-17.576000	3.580000
H	1.130000	-17.265000	4.610000
C	2.511000	-17.724000	2.902000
C	3.756000	-17.425000	3.447000
C	4.983000	-17.400000	2.776000
C	6.203000	-16.882000	3.337000
H	6.319000	-16.524000	4.351000
C	7.133000	-16.892000	2.340000
C	6.498000	-17.463000	1.171000
C	7.112000	-17.659000	-0.086000
C	6.477000	-18.250000	-1.199000
C	7.133000	-18.598000	-2.440000
C	6.187000	-19.157000	-3.249000
C	4.947000	-19.140000	-2.518000
C	3.711000	-19.541000	-3.036000
H	6.312000	-19.541000	-4.253000
H	8.181000	-18.447000	-2.653000
H	8.152000	-16.539000	2.389000
C	2.470000	-19.443000	-2.411000
N	2.306000	-18.122000	1.598000
N	5.149000	-18.603000	-1.269000
N	5.186000	-17.762000	1.466000
H	3.770000	-17.108000	4.486000
H	3.716000	-19.919000	-4.054000
C	-7.997000	-17.280000	-0.084000
C	-7.392000	-17.903000	-1.198000
N	-6.084000	-18.322000	-1.268000
C	-5.909000	-18.868000	-2.517000
C	-4.695000	-19.331000	-3.036000
C	-3.451000	-19.295000	-2.410000
C	-2.194000	-19.628000	-3.032000
H	-2.079000	-19.986000	-4.046000
C	-1.211000	-19.362000	-2.108000
C	-1.893000	-18.898000	-0.916000
C	-1.207000	-18.547000	0.262000
C	-1.875000	-18.142000	1.435000
C	-1.172000	-17.825000	2.661000
C	-2.137000	-17.492000	3.581000
C	-3.404000	-17.577000	2.903000
C	-4.634000	-17.215000	3.448000
C	-5.858000	-17.130000	2.777000
N	-6.079000	-17.481000	1.467000
C	-7.050000	-16.551000	3.338000
C	-7.980000	-16.514000	2.341000
H	-8.979000	-16.110000	2.390000
H	-7.147000	-16.187000	4.352000
H	-2.003000	-17.187000	4.610000
N	-3.220000	-17.985000	1.598000
N	-3.242000	-18.861000	-1.118000
C	-7.149000	-18.824000	-3.248000
H	-7.293000	-19.201000	-4.252000
Zn	-4.666000	-18.198000	0.183000
C	-8.065000	-18.217000	-2.439000
H	-9.104000	-18.014000	-2.651000
C	-7.374000	-17.116000	1.172000
H	-4.719000	-19.709000	-4.053000
H	-4.631000	-16.898000	4.486000
H	1.065000	-20.064000	-4.047000

6.2. Strain energy calculations

Strain energy for aromatized nanorings (**7** and **8**) and fused nanorings (**9** and **11**) was estimated using appropriate homodesmotic reactions shown below (Scheme S3).

Geometry of the substrates for the homodesmotic reactions were first optimized by finding the lowest-energy conformer with CREST program implemented into the xTB package (xTB-GFN2).[9,10] Then, DFT calculations on a B3LYP 6-31G(d,p) level of theory were performed.

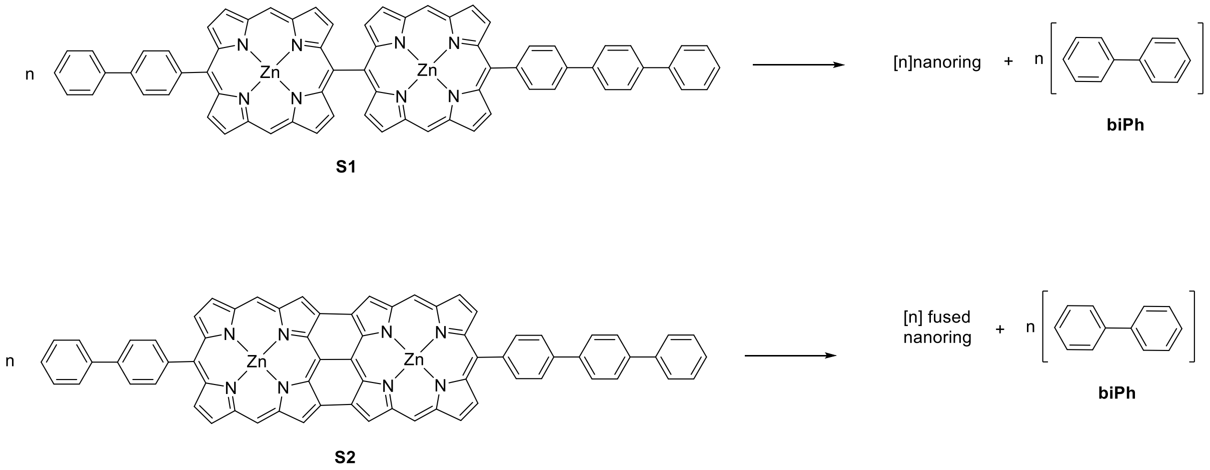

Scheme S3. Homodesmotic reactions used for estimation of strain energies.

Reference	H [Hartree]	SE [Hartree]	SE [kJ/mol]
**7**	-18675.50426	0.058592	154
**8**	-24900.70761	0.042858	112
**9**	-18668.55315	0.058754	154
**11**	-24891.43884	0.043692	115
**biPh**	-463.13039		
**S1**	-6688.318006		
**S2**	-6686.001023		

Table S3. Parameters used for DFT calculations and calculated strain energies.

6.3. MO contours for **7** and **9**.

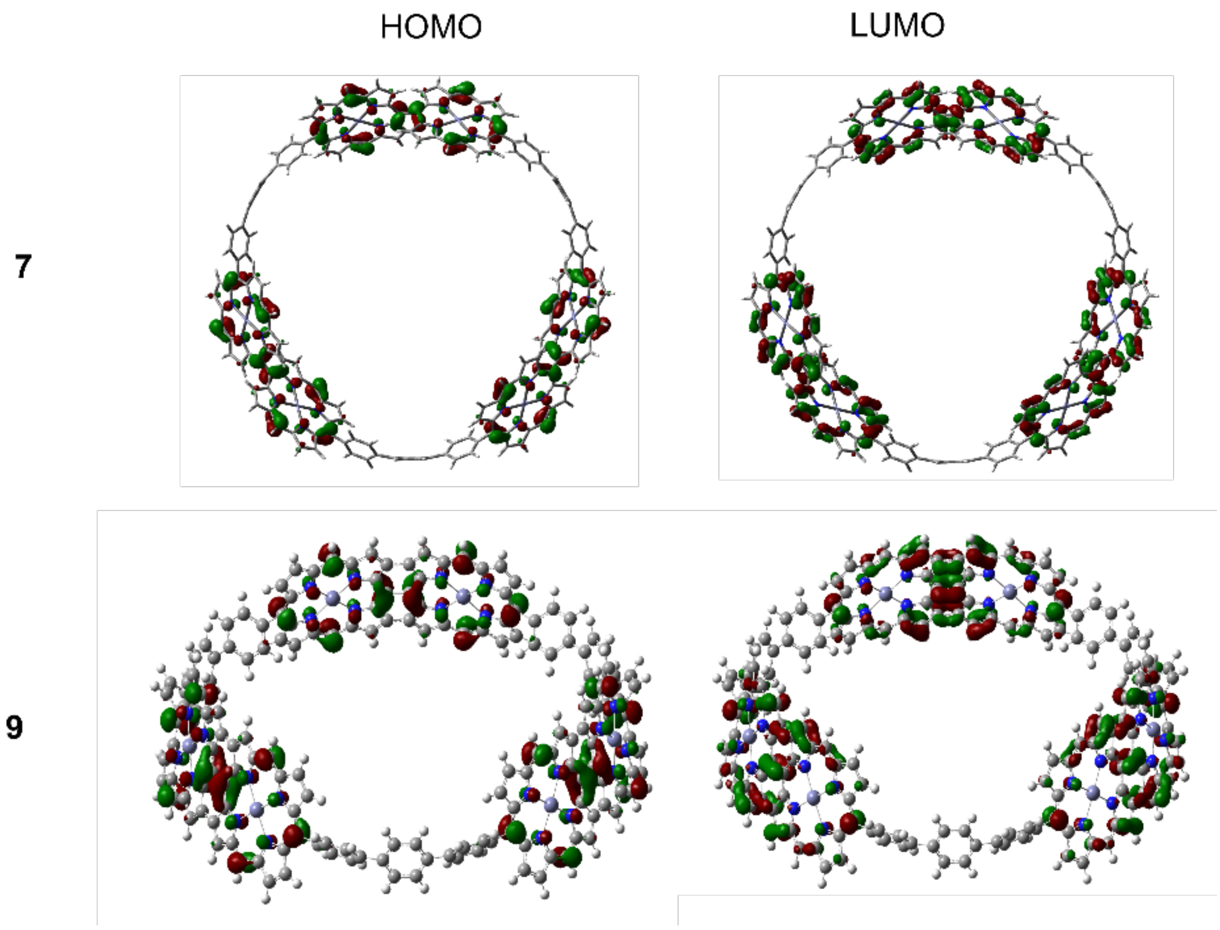

Figure S83. Calculated Kohn-Sham HOMO and LUMO orbitals for **7** and **9**.

6.4. Radius of curvature for **7** and **9**

The radius of curvature *R* was calculated from the DFT coordinates of **7** and **9**, by considering both the porphyrin units (coordinates of *meso* carbons, four in total) and the *p*-phenylene bridges (coordinates of carbon atoms at *para* positions, six in total). Cartesian coordinates in the plane of the bridge (Table S4) were fitted to the equation of a circle to get value of a bending radius using Origin software. For **7** *R* = 17.1 Å and 11.7 Å for porphyrins and *p*-phenylenes respectively, and upon fusion of porphyrins to compound **9** *R* = 19.0 Å (value similar to a hypothetical fully fused [14]porphyrin nanobelt) and 11.4 Å (value similar to the [16]cycloparaphenylene).

7, porphyrins

x (Å)	*y* (Å)
0.0000	0.0000
3.4530	5.9808
4.5577	7.0048
10.4000	10.6310

7, phenylenes

x (Å)	*y* (Å)
0.0000	0.0000
1.4230	2.4647
2.3810	3.5986
4.6116	5.3709
5.9307	6.0457
8.6199	6.9281

9, porphyrins

x (Å)	*y* (Å)
0.0000	0.0000
3.4435	5.9643
4.4431	7.0473
10.1060	10.9670

9, phenylenes

x (Å)	*y* (Å)
0.0000	0.0000
1.4240	2.4664
2.3619	3.6148
4.5620	5.4156
5.8781	6.0997
8.5782	6.9987

Table S4. Coordinates used to calculate radii of curvature.

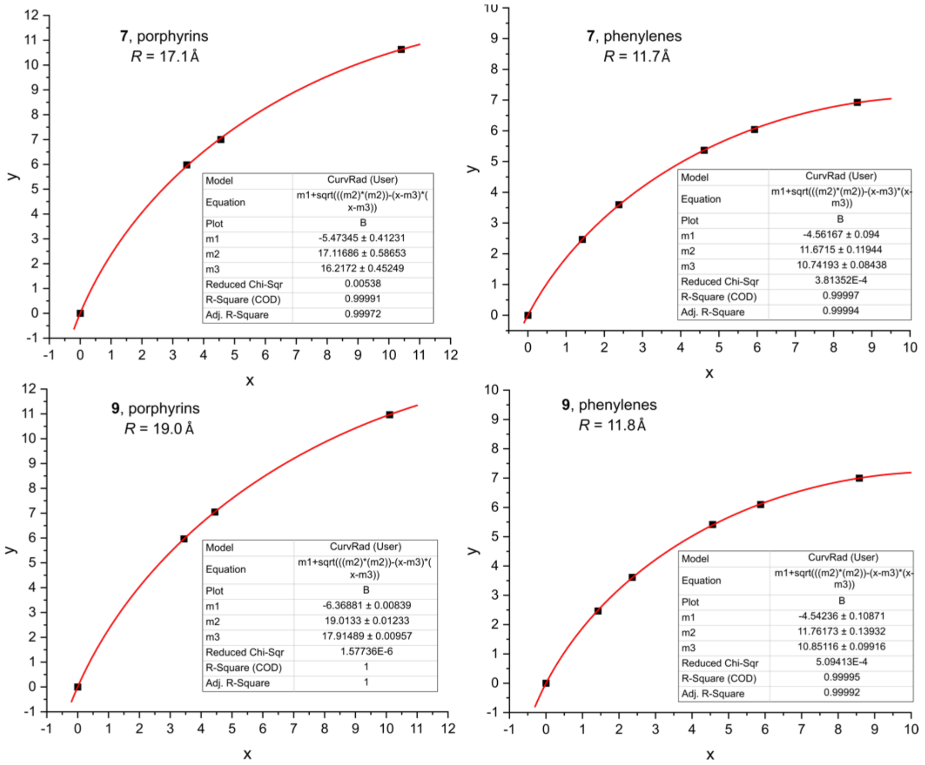

Fig S84. Fitting curves for bending radii. The radius of curvature *R* is the parameter m2.

7. Electrochemistry

Voltammetry measurements were made using an Autolab PGSTAT 12 with a 3 mm glassy carbon working electrode, platinum wire counter electrode and $Ag/AgNO_3$ (0.01 M in acetonitrile) reference electrode. Voltammograms were referenced to the Fc/Fc^+ couple (0.0 V) as an internal reference. Square-wave (SW) voltammograms were acquired with a 5 mV step potential, 50 mV modulation amplitude and 2 Hz frequency. The supporting electrolyte was tetra-*n*-butylammonium hexafluorophosphate (Bu_4NPF_6, TBAP) Inhibitor-free CH_2Cl_2 (from the SPS system) was added to the dry electrolyte to achieve an electrolyte concentration of 0.10 M. Analyte solutions were prepared by addition of this electrolyte solution to a particular compound (2–5 mg).

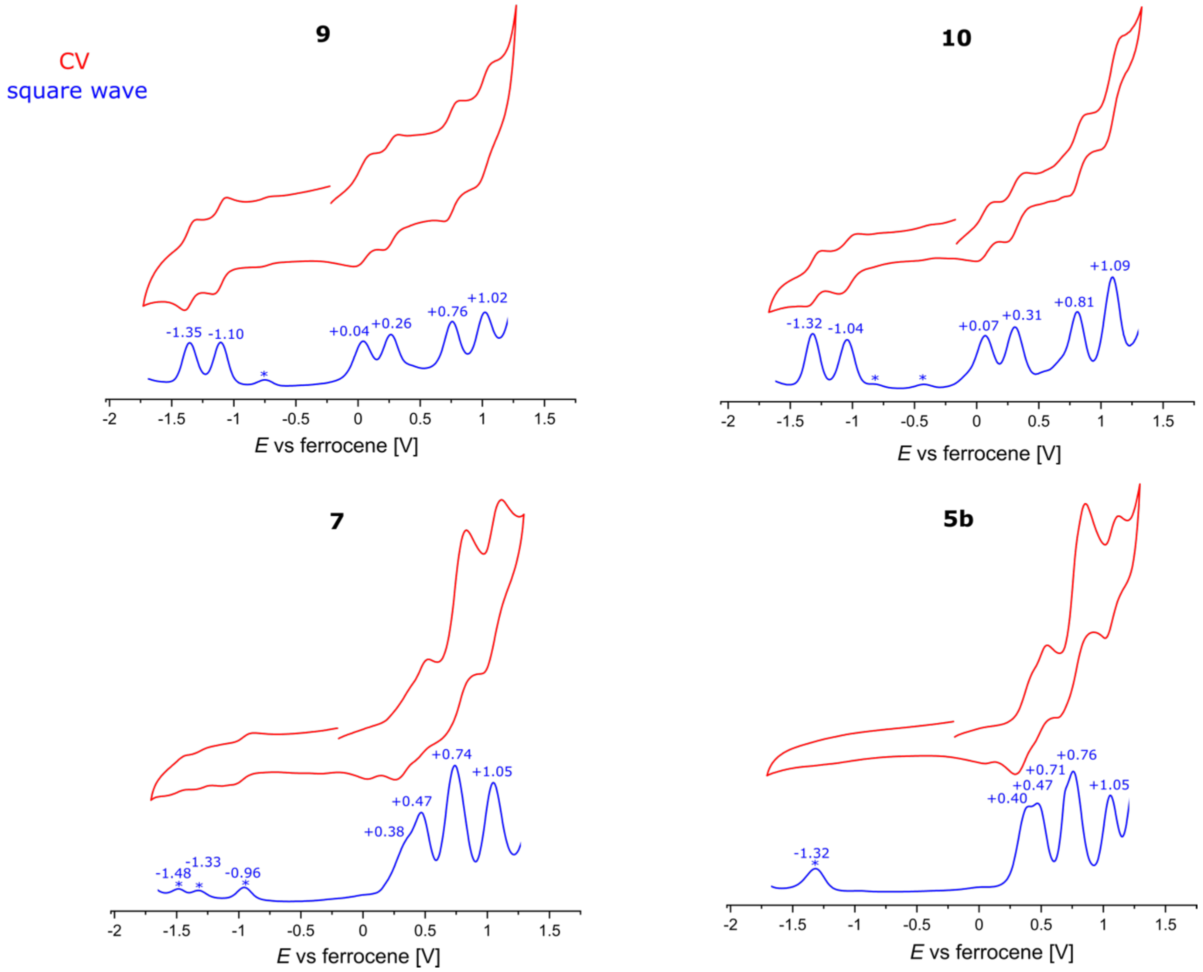

Figure S77. Cyclic (red) and square wave (blue) voltammograms measured for compounds **5b**, **7**, **9** and a reference compound **10**. Asterisks indicate waves that probably correspond to impurities.

8. References

[1] (a) Patel, V. K.; Kayahara, E.; Yamago, S. *Chem. – Eur. J.* **2015**, *21*, 5742–5749. (b) Mynos, D. M.; Ono, R. J.; Bielawski, C. W.; Sessler, J. L. *J. Mater. Chem*. **2012**, *22*, 18956–18960. (c) Yamamoto, Y.; Kato, K.; Shimizu, D.; Tanaka, T.; Osuka, A. *Chem. Asian J.* **2019**, *14*, 4021–4034.

[2] Chen, J.; Aratani, N.; Shinokubo, H.; Osuka, A., *Chem. – Asian J.* **2009**, *4*, 1126–1133.

[3] Abdulkarim, A.; Hinkel F.; Jänsch, D.; Freudenberg J.; Golling, F. E.; Müllen, K. *J. Am. Chem. Soc.* **2016**, *138*, 16208–16211.

[4] Coles, S. J.; Allan, D. R.; Beavers, C. M.; Teat, S. J.; Holgate, S. J. W.; Tovee, C. A. Leading edge chemical crystallography service provision and its impact on crystallographic data science in the twenty-first century. In *21st Century Challenges in Chemical Crystallography I: History and Technical Developments*; Mingos, D. M. P., Raithby, P. R., Eds.; Structure and Bonding; Springer International.

[5] Dolomanov, O. V.; Bourhis, L. J.; Gildea, R.J, Howard, J. A. K.; Puschmann, H. *J. Appl. Cryst.* **2009**, *42*, 339–341.

[6] Sheldrick, G. M. *Acta Cryst.* **2015**, *C71*, 3–8.

[7] Gaussian 16, Revision C.01, M. J. Frisch, G. W. Trucks, H. B. Schlegel, G. E. Scuseria, M. A. Robb, J. R. Cheeseman, G. Scalmani, V. Barone, G. A. Petersson, H. Nakatsuji, X. Li, M. Caricato, A. V. Marenich, J. Bloino, B. G. Janesko, R. Gomperts, B. Mennucci, H. P. Hratchian, J. V. Ortiz, A. F. Izmaylov, J. L. Sonnenberg, D. Williams-Young, F. Ding, F. Lipparini, F. Egidi, J. Goings, B. Peng, A. Petrone, T. Henderson, D. Ranasinghe, V. G. Zakrzewski, J. Gao, N. Rega, G. Zheng, W. Liang, M. Hada, M. Ehara, K. Toyota, R. Fukuda, J. Hasegawa, M. Ishida, T. Nakajima, Y. Honda, O. Kitao, H. Nakai, T. Vreven, K. Throssell, J. A. Montgomery, Jr., J. E. Peralta, F. Ogliaro, M. J. Bearpark, J. J. Heyd, E. N. Brothers, K. N. Kudin, V. N. Staroverov, T. A. Keith, R. Kobayashi, J. Normand, K. Raghavachari, A. P. Rendell, J. C. Burant, S. S. Iyengar, J. Tomasi, M. Cossi, J. M. Millam, M. Klene, C. Adamo, R. Cammi, J. W. Ochterski, R. L. Martin, K. Morokuma, O. Farkas, J. B. Foresman, and D. J. Fox, Gaussian, Inc., Wallingford CT, 2016.

[8] a) Lee, C. T.; Yang, W. T.; Parr, R. G. *Phys. Rev. B*, **1988**, 37, 785–789. b) Becke, A. D. *Phys. Rev. A* **1988**, *38*, 3098–3100.

[9] Pracht, P.; Bohle, F.; Grimme, S. *Phys. Chem. Chem. Phys*. **2020**, *22*, 7169–7192.

[10] Bannwarth, C.; Ehlert, S.; Grimme, S. *J. Chem. Theory Comput.* **2019**, *15*, 1652–1671.
